# Supplementary material for: A Generative Neuro‐Symbolic AI for Protein Sequence Design
Source: Adv Sci (Weinh). 2026 Jul 30:e76464. Online ahead of print. doi: 10.1002/advs.76464 (PMC13422582; doi:10.1002/advs.76464)
Supplement: Supplementary file 1 — Supporting File: advs76464‐sup‐0001‐SuppMat.pdf. [file ADVS-9999-e76464-s001.pdf]

Supporting Information
A Generative Neuro-Symbolic AI for Protein Sequence Design

Marianne Defresne[†,1,2], Delphine Dessaux[†,1], Samuel Buchet[2], Lucie Barthe[1], Liza Ammar-Khodja[3], Bessam Azizi[1], Valentin Durante[2], Gianluca Cioci[1], Simon de Givry[2], Alain Roussel[3], Luis F. Garcia-Alles[1], Thomas Schiex[2,*], and Sophie Barbe[1,*]

[1]TBI, Université de Toulouse, CNRS, INRAE, INSA, ANITI, 31077 Toulouse, France
[2]MIAT, Université de Toulouse, ANITI, INRAE, UR 875, 31326 Toulouse, France
[3]LISM, CNRS, Université d'Aix-Marseille, Marseille, France

[†]M.D. and D.D. contributed equally to this work.
[*]Corresponding authors: thomas.schiex@inrae.fr; sophie.barbe@insa-toulouse.fr

Contents

1 A Steerable Deep-learned Score Function for Protein Sequence Design . . . 4
1.1 Ablation study . . . 4
1.2 Hyperparameters . . . 4
1.3 Exact optimization vs LR-BCD . . . 5
1.4 Exact optimization vs bounded-optimality proofs . . . 5

2 Design of BMC-H proteins . . . 5
2.1 Implementation of multi-state design approaches . . . 5
2.1.1 Multi-state design with ProteinMPNN . . . 5
2.1.2 Multi-state design with Effie and bi-objective optimization . . . 6
2.2 Details and discussion on the in silico comparative analysis of EffieDes and ProteinMPNN on BMC-H designs . . . 12
2.3 Supplementary material and methods for experimental characterization of BMC-H proteins . . . 13
2.3.1 Cloning . . . 13
2.3.2 Protein expression, solubility and purification . . . 13
2.3.3 Western blot analysis . . . 13
2.3.4 Size-exclusion chromatography . . . 13
2.4 Discussion on experimental characterization of BMC-H designs . . . 14
2.4.1 Analysis of protein contents by SDS-PAGE . . . 14
2.4.2 Copurification using Flag-peptide . . . 14
2.4.3 Size-exclusion chromatography . . . 14
2.5 Figures and Tables . . . 15

3 De novo design of nanobodies . . . 41
3.1 Supplementary Material and Methods . . . 41
3.1.1 Western blot analyses of nanobodies expression . . . 41
3.1.2 Expression and purification of SARS-CoV-2 RBDs, and MR17 and NbRM-E1 nanobodies . . . 41
3.1.3 Molecular Dynamics simulations of RBD-nanobody complexes . . . 41
3.2 Figures and Tables . . . 43

Supplementary Figures

S1 Similarity to the native sequence (NSR) of sequences designed with exact optimization or LR-BCD. 5
S2 Example of a Pareto front for our (min, max) negative design problem. 7
S3 Illustration of the selection of a candidate solution (function *GetCandidate*) to form new pairs of weights in our ApproximationDichotomy algorithm. 8
S4 Solutions and lower-bound curve obtained with ApproximationDichotomy algorithm on the two designable regions. 9
S5 Comparison of the solutions by EffieDes in the two designable regions. 11
S6 Representation of BMC-H homohexamer in a bacterial microcompartment (BMC) shell. 15
S7 Visualization of the residues defined in the small and large regions. 16
S8 Comparison of pre-minimization scores for mutant sequences generated on the large region. . . . 17
S9 Comparison of post-minimization scores for mutant sequences generated on the small region. . . 18
S10 Comparison of post-minimization scores for mutant sequences generated on the large region. . . . 19
S11 Comparison of averaged score differences $\overline{\Delta A}$ and $\overline{\Delta B}$ for the small and large designable regions. 20
S12 Comparison of Effie inter-chain scores for sequences predicted on the small region. 21
S13 Comparison of Effie inter-chain scores for sequences predicted on the large region. 22
S14 RMSD of the backbone of the hexamer as a function of their score Effie for sequences predicted on the small region. 23
S15 RMSD of the backbone of the hexamer as a function of their score ProteinMPNN for sequences predicted on the small region. 24
S16 Expression and solubility of the Duo designs. 25
S17 Experimental screening of the Duo designs using the tripartite GFP technology. 26
S18 Coprecipitation experiments of monomers A and B from selected Duo designs. 27
S19 Characterization of the oligomeric state of Duo4 by chromatography. 28
S20 Expression of designed nanobodies verified by western blot on total cell extracts. 43
S21 Interaction of designed nanobodies with SARS-CoV-2 RBD XBB.1.16 assessed by BLI experiments on bacterial extracts. 43
S22 Purification by Nickel-affinity chromatography of the NbRM-E1 nanobody. 44
S23 Purification by gel filtration of the NbRM-E1 nanobody. 45
S24 BLI-based determination of the dissociation constant K_D for the NbRM-E1/XBB.1.16 RBD binding. 46
S25 BLI specificity assay of NbRM-E1 binding to SARS-CoV-2 RBDs. 46
S26 BLI specificity assay of MR17 nanobody binding to SARS-CoV-2 RBD variants. 47
S27 SARS-CoV-2 mutations affecting MR17 interaction with the RBD. 47
S28 3D structural model of MR17 in complex with the XBB.1.16 RBD. 48
S29 3D models of the competitive binding of NbRM-E1 with ACE2 and VHH-72 on the XBB.1.16 RBD. 48
S30 Selection of designed nanobodies based on interaction with RBD during MD simulations. 49

Supplementary Tables

S1 Ablation study performed on the single-chain test set. 4
S2 Evaluation of EffieDes or ProteinMPNN designs for each designable region. 29
S3 Evaluation of inter-chain Effie scores for EffieDes or ProteinMPNN designs. 29
S4 Evaluation of the sequences selected for experimental characterization by Effie scores. 30
S5 Evaluation of the sequences selected for experimental characterization by ProteinMPNN scores. . 31
S6 Evaluation of the sequences selected for experimental characterization by Effie inter-chain scores and RMSD. 32
S7 Amino acid sequences of recombinant BMC-h proteins and number of incorporated mutations compared with WT RMM. 33
8 BMC-H Duo DNA sequences for tripartite GFP assays. 35
9 BMC-H Duo DNA sequences for FLAG/His6 assays. 39
S10 Rosetta InterfaceAnalyzer metrics of RBD/nanobody complexes for nanobodies selected for experimental characterization. 50
S11 Interaction between nanobodies and RBDs calculated on 50 ns MD simulation. 51

S12 Interaction between nanobodies and RBDs calculated on the last 10 ns of MD simulation. 51
S13 Amino acid sequences of designed nanobodies and number of mutations relative to MR17. 52

1 A Steerable Deep-learned Score Function for Protein Sequence Design

1.1 Ablation study

We made sure that all the major components of our architecture are useful through an ablation study displayed in Table S1. Results were obtained with the anterior version of Effie[9] on the single-chain test set and by optimizing with LR-BCD. This version can be considered as an ablation of the current version without iterations over the ResMLP+gMLP bloc to extract features environment.

Table S1. Ablation study performed on the single-chain test set.

Model	NSR
Effie	48.4%
Effie from [9] (no iterations over ResMLP+gMLP bloc)	42.9%
Regular PLL ($p = 0$)	40.6%
No environment information (*i.e.*, no ResMLP+gMLP bloc)	33.5%
No sequence information (feature $i - j$)	41.2%
No distance cut-off at 15 Å between residue pairs	40.3%
No L1 regularization on learned costs	41.9%

Interestingly, even though the E-PLL was developed to deal with logical information, it also proved beneficial in the case of protein data as it lead to a 2.3% increase in NSR. We hypothesize this improvement comes from the existence of high energies in CPD problems, that cannot be estimated with the regular PLL.

Removing the dedicated neural net to extract the neighbour information of each residue is the ablation that lead to the largest drop in performances, which is coherent with physics-based intuition. The distance cut-off on pairs of residue is beneficial in terms of NSR, but also in terms of memory usage and optimization time. It can be seen as an inductive bias, as far apart residues do not interact. Similarly, the L1 regularization, by encouraging the predicted costs to be sparse, speeds up optimization while improving NSR.

1.2 Hyperparameters

The values of each hyperparameters have been chosen by fixing all parameters saved one and training a model whose performances was assessed by its NSR on the validation set. In the case where two models had similar NSR, we applied parsimony and chose the simplest model (*e.g.,* with fewer parameters or more regularization).

The features of each pair of amino acids within 15 Å are 25 distances between backbones atoms (encoded with 16 Gaussian radial basis functions with centers evenly spaced between 0 and 20 Å); positional encoding of $|j - i|$ (size 16). For each amino acids, 128 nearest neighbours are considered (with 0-padding with the protein is shorter than 128.

The neural architecture is composed of a ResMLP with 3 blocks of 2 layers with residual connections which embeds the input to dimension 256. Then a second ResMLP with the same shape and a gMLP[23] with 3 layers, a width of 2×256 and a sequence length of 48 are iteratively applied (6 times). Finally, the resulting environment embedding is processed by a third ResMLP (3 blocks of size 2 and width 256) to produce cost functions. The complete architectures contains 3.4M parameters.

For the loss, the Emmental-NPLL[9] is used with a parameter $p = 0.3$, meaning that 30% of residues are randomly masked to compute the NPLL. Only binary terms (*i.e.,* cost functions between pairs of residues) are used. The learned score functions are regularized using an L1-norm with a regularization weight of 10^{-4}.

For training, the Adam optimizer was used with a weight decay of 10^{-3} and an initial learning rate of 5.10^{-4}. Other values take default parameters. A scheduler divides the LR by 10 each time the validation loss decreases, with patience 0. In the case of multichain proteins, we found a lower initial learning rate ($5\ 10^{-5}$) was beneficial. As proteins are larger (up to $10,000$ amino acids vs fewer than 500 for single-chain proteins), we designed an adaptive learning rate. We changed the learning rate for each protein such that it was proportional to the square root of the number of variables (similar to batch size). Only multichain proteins shorter than $1,000$ amino acids were considered.

1.3 Exact optimization vs LR-BCD[11]

EffieDes offers two optimization algorithm from toulbar2:[18] exact or the efficient approximation LR-BCD. To ensure the quality of LR-BCD solutions, we compared them with the solutions, guaranteed to be optimal, obtained by the exact solver. We fully redesigned with both methods all the proteins from the single-chain dataset, which are shorter than 150 residues. Solutions found by LR-BCD are in average at 0.56% of the optimal Effie score, so LR-BCD provide protein sequences close to the optimum. We also compared the sequences designed by both solver with native sequences in terms of similarity. Results are plotted in Figure S1. Sequences by LR-BCD have a similar NSR to those obtained by exact optimization.

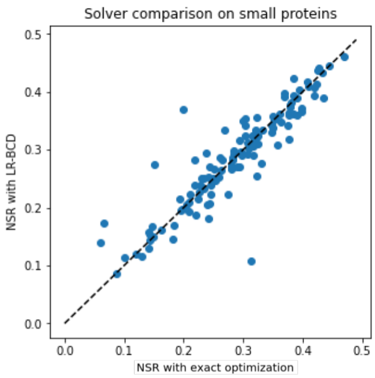

Figure S1. Similarity to the native sequence (NSR) of sequences designed with exact optimization or LR-BCD.

1.4 Exact optimization vs bounded-optimality proofs[2]

Besides LR-BCD, toulbar2 offers the ability to trade computational time for quality by offering bounded quality proofs. Although we have not extensively evaluated the approach on large protein design problems, we tested this on the full redesign of a randomly chosen 1,782 amino acid structure (PDB ID: 11SZ). We used the "Design.py" script from EffieDes' GitHub repo with the `-s` flag to save the Cost Function Network model as a separate file, which could then be solved using dedicated toulbar2 algorithms. We used the `toulbar2 -A -pils <CFN file>` command to solve the corresponding instance. This command preprocessed the instance targeting both strong lower (-A) and upper (-pils) bounds. On a single core of a MacBook M3 laptop, a solution with a score of -8,907.920 was produced after only seven minutes. At the same time, a lower bound of -9,052.856 was made available. This proved that the solution was within 1.60% of the global optimum. For reference, the native sequence was evaluated using the provided `Effie-Energy.py` script, showing a score of -7,078.693. On this randomly chosen protein, seven minutes of optimization therefore already brought us very close to the bottom of the Effie funnel. A more extensive analysis of the trade-offs between score quality and computational time, also in comparison with sequence sampling approaches, would be needed to evaluate these trade-offs.

2 Design of BMC-H proteins

2.1 Implementation of multi-state design approaches

2.1.1 Multi-state design with ProteinMPNN

ProteinMPNN is an auto-regressive model that learned the probability distribution $P(\mathbf{s}_i \mid \mathbf{s}_{<i}, B)$ over amino acids for a residue at position i given a state B and the identity of previously fixed amino acids $s_{<i}$. Applied iteratively on a protein with ℓ residues, using the chain rule

$$P(\mathbf{s}|B) = P(\mathbf{s}_1|B)P(\mathbf{s}_2|\mathbf{s}_1, B) \cdots P(\mathbf{s}_\ell|\mathbf{s}_1, \ldots, \mathbf{s}_{\ell-1}, B)$$

ProteinMPNN can directly sample $P(\mathbf{s}|B)$, the conditional probability of sequences $\mathbf{s}$ given the target state B. Using low temperature at each auto-regressive step favors the sampling of high-probability sequences.

It is important to note that the marginal conditional probabilities $P(\mathbf{s}_i|\mathbf{s}_{<i}, B)$ that ProteinMPNN has learned to predict assume that the future amino acids $\mathbf{s}_{>i}$ will also be sampled from the same model. If this is not the case, these predicted marginal conditional probabilities may become arbitrarily wrong and sampling may eventually explore low-probability regions.

For symmetrical design, ProteinMPNN can tie symmetrical residues together. When a tied position is sampled, the shared amino acid identity is chosen using the sum of the logits of the tied residues.

For multi-state design, the authors of ProteinMPNN[8] suggest using a linear combination of the logits obtained on different states with positive and negative weights, λ^+ and λ^-, to favor or disfavor chosen states. The GitHub repository for the software (`https://github.com/dauparas/ProteinMPNN`) contains a helper script `make_pos_neg_tied_positions_dict.py` for this purpose.

Assuming that a positive state B_+, (weight 1) and a negative state B_- (weight -1) are used, this means that each amino acid will be sampled from a distribution $P \propto \frac{P(\mathbf{s}_i|\mathbf{s}_{<i}, B_+)}{P(\mathbf{s}_i|\mathbf{s}_{<i}, B_-)}$ instead of the respective distributions $P(\mathbf{s}_i|\mathbf{s}_{<i}, B_+)$ and $P(\mathbf{s}_i|\mathbf{s}_{<i}, B_-)$ that ProteinMPNN auto-regressive model assumes on either state. This may alter its accuracy in unpredictable ways.

For our design objective, we determined the most suitable weight combinations to be, for a fixed positive weight of $\lambda^+ = 1$, $-0.45 < \lambda^- < -0.25$. Indeed, if $\lambda^- < -0.45$, the formation of the heterohexamer AB becomes energetically unfavorable (see Figures S14 and S15). On the other hand, if $\lambda^- > -0.25$, homohexamers AA and BB become favorable. The weight combinations involving λ^- in this range were explored with a finer resolution than the ones with $\lambda^- > -0.25$ or $\lambda^- < -0.45$.

2.1.2 Multi-state design with Effie and bi-objective optimization

Problem setting As explained in the manuscript, given a state B, the neural architecture of Effie predicts the score function $E(\mathbf{s}|B)$ as a pairwise decomposable score function, i.e., a sum of terms that each involves two residues:

$$E(\mathbf{s}|B) = \sum_{i<j} E_{ij}(\mathbf{s}_i, \mathbf{s}_j|B) \tag{S1}$$

We assume that we have positive and negative rigid backbone states $B = B_+ \cup B_-$, all with the same number ℓ of residues. In this setting, B_- incorporates the two homomeric states, AA and BB. At each position $1 \leq i \leq \ell$ of a given designable region (small or large), we have a set S_i of possible amino acids. When designing multi-component symmetrical proteins, the identity of a given component's i^{th} residue will be the same in all its occurrences in each subunit of the symmetrical assembly in Eq. (S1). Every variable that does not represent the first occurrence of a component can therefore be replaced by the corresponding variable in the component's first occurrence. This drastically reduces the number of variables in the above sum. For a C6 monomeric hexamer, such as 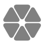 or 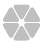, this reduces the number of variables by 6. For a two-component C3 hexamer, such as 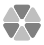, the number of variables is reduced by 3. These reductions are applied systematically later on.

Intuitively, the multi-state sequence design (MSD) problem is to find a sequence $\mathbf{s}$ which minimizes:[37]

$$E(\mathbf{s}|B_+) - E(\mathbf{s}|B_-)$$

Minimizing this difference means minimizing the score of the designed sequence $\mathbf{s}$ on positive states and maximizing it on negative states, thus favoring positive against negative states. Alternatively, different compromises of the two objectives may be worth exploring, whereas this single combination may favor the minimizaton of the positive state to the detriment of maximization the negative one, or *vice-versa*. Given $E(s|B_+) = E^+(s)$ that is to be minimized and $E(s|B_-) = E^-(s)$ that is to be maximized, a more sophisticated formulation consists of defining the following bi-objective optimization problem:

$$\begin{aligned} \underset{\mathbf{s}}{min} \quad & E^+(\mathbf{s}) \\ \underset{\mathbf{s}}{max} \quad & E^-(\mathbf{s}) \end{aligned} \tag{S2}$$

With such a formulation, any solution (sequence) $\mathbf{s}$ of the problem is associated to two different scores (one for each objective) forming a score-pair: $(E^+(\boldsymbol{s}), E^-(\boldsymbol{s}))$. In this context, a given sequence $\mathbf{s}$ is said to dominate another sequence $\mathbf{t}$ (informally "$\mathbf{s}$ is better than $\mathbf{t}$"), if $\mathbf{s}$ has a strictly better score for one objective and an

equal-or-better score for the other one, i.e., either $E^+(\mathbf{s}) < E^+(\mathbf{t})$ and $E^-(\mathbf{s}) \geq E^-(\mathbf{t})$, or $E^+(\mathbf{s}) \leq E^+(\mathbf{t})$ and $E^-(\mathbf{s}) > E^-(\mathbf{t})$. Two sequences that do not dominate each other are otherwise considered incomparable. The goal of multi-objective optimization is then to find a complete set of score-pairs corresponding to incomparable sequences that are not dominated by any other sequences. This set of exhaustive optimal score-pairs is usually designated as the Pareto front.[12] From a decision-making perspective, such outcome allows a designer to review a large number of candidate sequences where positive and negative scores are diversely balanced. Figure S2 shows an example of a Pareto front (score-pairs of Pareto-optimal solutions) for a *min* and *max* bi-objective optimization problem.

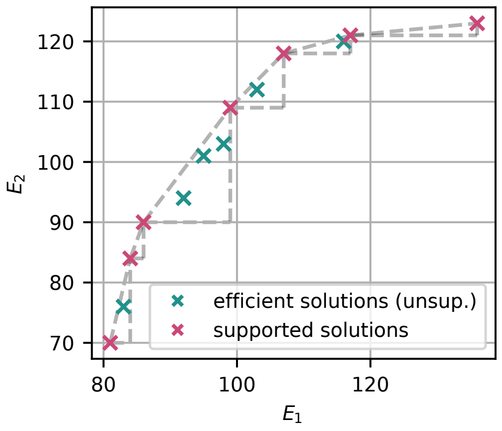

Figure S2. Example of a Pareto front for our (min, max) negative design problem. Red solutions (called supported solutions) are located on the convex hull of the Pareto front. Other (unsupported) solutions are represented in green.

Given that the score function E predicted by Effie is pairwise decomposable, the discrete bi-objective optimization problem is known to be #P-hard,[12] whereas its single-objective counterpart is NP-hard.[31] Practically speaking, obtaining solutions of the bi-objective problem is usually more expensive due to the mixing of the two objectives. Although challenging, these problems are, however, considerably simpler than the full-atom physics-based formulation which was proven to be at the second level of Stockmeyer's Polynomial Hierarchy (*i.e.*, $\mathsf{NP}^{\mathsf{NP}}$-complete or Σ_2^p-complete).[35,37]

Sequence generation by approximating Pareto frontiers One way of approximating Pareto fronts is to rely on single-objective optimization tools such as toulbar2. This can be performed by defining a pair of weights λ^+ and λ^- and minimizing the following weighted score:

$$\min_{\mathbf{s}} \quad \lambda^+ E^+(\mathbf{s}) + \lambda^- E^-(\mathbf{s}) \tag{S3}$$

Where $\lambda^+ \geq 0$ and $\lambda^- \leq 0$. This corresponds to a scalarized formulation of the bi-objective problem (S2). Varying the two multipliers enables the tuning of the contribution of positive versus negative states. More precisely, when solved to optimality this formulation yields a Pareto-optimal solution located on the convex hull of the Pareto front, designated as a supported solution in Figure S2. Our approach to solving problem (S2) is based on a variant of the Dichotomy method[3] which we call the approximation dichotomy algorithm. The original dichotomy algorithm repetitively optimizes the linear combination of the two objectives (S3) using different weights to perform an exhaustive enumeration of non-dominated (supported) score-pairs starting from the extreme weights-pairs $(1, 0)$ and $(0, 1)$. Each time a new provably optimal solution is found, an intermediate pair of weights is computed, and the search is repeated until all possible supported score-pairs obtainable by optimizing (S3) are produced.

Optimizing the NP-hard formulation (S3) is still challenging with exact optimization tools, even if one relies on the efficient provable algorithms. We therefore modified the Dichotomy method to account for potential suboptimal solutions returned after solving with a given pair of weights. Our modified algorithm called ApproximationDichotomy benefits from the mathematical guarantees of exact optimization algorithms (producing valid

bounds), as well as the ability of heuristic solvers to rapidly produce high-quality solutions (without guarantee of optimality). The overall approach that solves problem (S2) is then decomposed into two phases that both use ApproximationDichotomy. In the first phase, it produces an initial coarse approximation of the Pareto front with provable bounds. In the second phase, the density of solutions is further increased using heuristic capabilities of single-objective solvers.

ApproximationDichotomy procedure Our approximation dichotomy algorithm relies on the call to a function $Solve(\lambda^+, \lambda^-, ub, t)$ which solves problem (S3). Such a function takes as input a pair of weights (λ^+, λ^-), an upper bound ub (sequences with score greater than ub are not sought) and a time limit t. When the time is exceeded, $Solve$ returns a tuple (S, l) where l is a lower bound on the optimum of (S3) and S is a set of solutions found during the search. These two output values allow one to construct proven bounds on the Pareto-optimal solutions. In the main loop of the algorithm, the construction of a new pair of weights is modified from the original Dichotomy method[3] through the use of the function $GetCandidate$ as illustrated in Figure S3. This function controls the progression of the Pareto front approximation, as well as the number of iterations of the algorithm when the output solutions of $Solve$ are not proven to be optimal. The general procedure $ApproximationDichotomy(t, m, Solve)$ takes as arguments a time limit t and a limit m on the number of calls to the $Solve$ procedure. It returns a lower bound L and an upper bound U on the Pareto front. The procedure is described in more details in Algorithm ApproximationDichotomy.

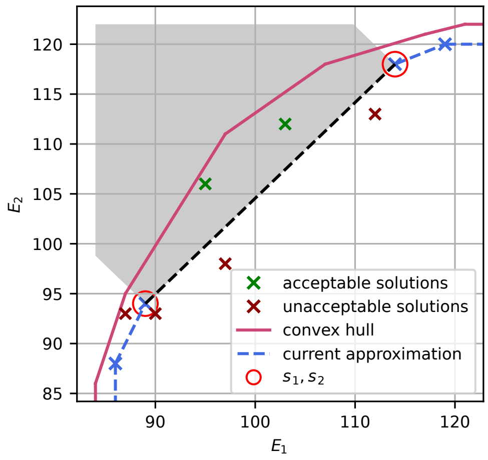

Figure S3. Illustration of the selection of a candidate solution (function $GetCandidate$) to form new pairs of weights in our ApproximationDichotomy algorithm. The green and red crosses represent solutions returned by $Solve$. The grey area represents the area where solutions can be considered to form new pairs of weights. In this area, the left green point (i.e. with the best scalarized score) is to be selected to produce two new pairs of weights by association with the input solutions $\boldsymbol{s_1}$ and $\boldsymbol{s_2}$.

Phase 1 : coarse approximation with exact bounds. Phase 1 of our algorithm performs a first iteration of the ApproximationDichotomy algorithm to provide an initial coarse approximation of the Pareto front. For given weights (λ^+, λ^-), the single objective (S3) is optimized using an interrupted exact optimization algorithm, providing a lower/upper bound pair (lb, ub) of the optimum. The use of exact algorithms enables to assert that for each possible solution $\mathbf{s}$:

$$\lambda^+ E^+(\mathbf{s}) + \lambda^- E^-(\mathbf{s}) \geq lb$$

Thus, the tuple $(\lambda^+, \lambda^-, lb)$ defines a half-space in the space of objective costs, in which we know that no solution exists. As we repeatedly optimize the scalarized score (S3) in the approximation dichotomy with various weights, the union of these half-spaces defines a bounding space on the Pareto front whose frontier is represented as an orange curve in Figure S4 (providing that the Pareto front is located on the lower right side of the orange curve). We call this curve a lower bound by analogy to the lower bounds produced by single-optimization algorithms. Together with incumbent solutions returned during each search, they delineate an approximation of the Pareto front in which the designer can choose sequences (Figure S5**A**).

Phase 2 : increase of the solution density and quality. The original Dichotomy algorithm only tries to identify the convex hull of the Pareto front, and the number of solutions needed to define it may be limited. To offer a wider diversity of designs, we generated more incumbent solutions by re-optimizing with stochastic metaheuristic optimization algorithms. In practice, the ApproximationDichotomy procedure is called with a $perturbedHeuristic(nbrepeat, \epsilon)$ procedure in place of *Solve*. This $perturbedHeuristic$ performs $nbrepeat$ call to a heuristic solver with a perturbed objective function (with the same scalarization weights) obtained by adding a small random uniform noise (in $[0, \epsilon]$) on the scalarized objective. To control the number and diversity of the resulting sequences, they are clustered using *MMseqs2*,[34] adjusting the similarity threshold to the number of desired solutions. A sequence can then be selected in each cluster. The parameters $nbrepeat = 5$ and $\epsilon = 0.005$ were chosen for the computational design.

Our method for approximating bi-objective Pareto fronts has been implemented with the Cost Function Network solver toulbar2[18] for its efficiency at optimizing pairwise-decomposable cost functions such as Effie,[16] (`https://github.com/toulbar2/toulbar2`). Phase 1 of the method uses its systematic provable optimization algorithm *Hybrid Best First Search (HBFS)*,[1] providing mathematically proven lower bounds on the objective. The second phase uses the Variable Neighborhood Search metaheuristic algorithm (VNS)[6,29] implemented in toulbar2. Since this phase focuses on the solutions, no lower bound L is returned.

Figure S4 shows the cost pairs of the solutions computed with our algorithm for the two designable regions. In total, 450 solutions were selected for the small region and 778 for the large one. The figure also depicts the lower bound curve obtained in the first phase. Although the bounding of the Pareto front is rather narrow for the small region, it is more uncertain in the larger one due to the increase of size of the optimization problems. Figure S5**A** provides a comparison of the two phases of the algorithm with respect to the approximate Pareto front. The second phase has the effect of smoothing the lower right side of the uncertain area, thus reducing its surface.

The source code of our method is available at `https://github.com/effiedes/boond`.

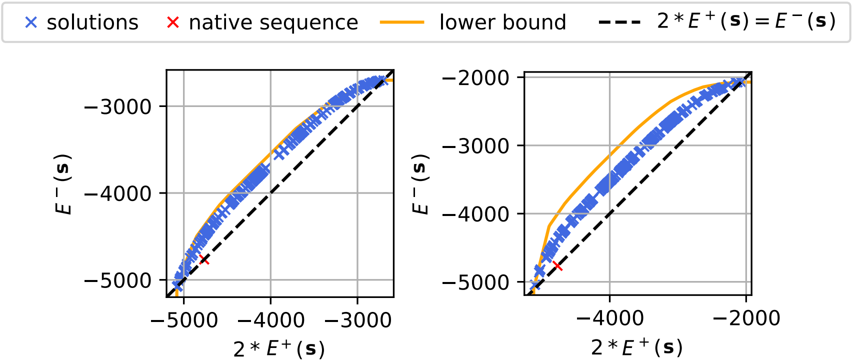

Figure S4. Solutions and lower-bound curve obtained with ApproximationDichotomy algorithm on the two designable regions: small (left) and large (right). The Pareto front of the problem must be located between the blue points and the orange curve. The slightly convex curve above the diagonal indicates to which point the scores of the positive state (X axis) and negative state (Y axis) can be pushed away. The native state appears on the diagonal as a red cross. Its position indicates which levels of Effie score should be sufficient to stabilize the considered fold.

Function ApproximationDichotomy($t, m, Solve$)

$L := \varnothing$; $U := \varnothing$; $Q := \varnothing$;
/* Solve $1 \times E^{+}(\boldsymbol{s}) + 0 \times E^{-}(\boldsymbol{s})$ within time t, store upper & lower bounds */
$S_1, l_1 :=$ Solve$(1, 0, \infty, t)$; $\boldsymbol{s_1^*} :=$ pop-first(S_1) ;
L.push$(\langle 1, 0, l_1 \rangle)$;
U.merge(S_1);
/* Solve $0 \times E^{+}(\boldsymbol{s}) - 1 \times E^{-}(\boldsymbol{s})$ within time t, store upper & lower bounds */
$S_2, l_2 :=$ Solve$(0, -1, \infty, t)$; $\boldsymbol{s_2^*} :=$ pop-first(S_2);
L.push$(\langle 0, -1, l_2 \rangle)$;
U.merge(S_2);
/* Proceed if starter solutions have been found */
if $\boldsymbol{s_1^*} \neq \varnothing \wedge \boldsymbol{s_2^*} \neq \varnothing$ **then**
  $Q := \{(\boldsymbol{s_1^*}, \boldsymbol{s_2^*})\}$
while $Q \neq \varnothing$ **and** $|L| \leq m$ **do**
  /* Bisect by (λ_1, λ_2)-scalarization and store bounds */
  $(\boldsymbol{s_1}, \boldsymbol{s_2}) :=$ pop-first(Q);
  $\lambda^{+} := E^{-}(\boldsymbol{s_1}) - E^{-}(\boldsymbol{s_2})$; $\lambda^{-} := E^{+}(\boldsymbol{s_2}) - E^{+}(\boldsymbol{s_1})$;
  $S, l :=$ Solve$(\lambda^{+}, \lambda^{-}, [\lambda^{+}E^{+}(\boldsymbol{s_1}) + \lambda^{-}E^{-}(\boldsymbol{s_2})], t)$;
  $\boldsymbol{s} :=$GetCandidate$(S, \boldsymbol{s_1}, \boldsymbol{s_2}, \lambda^{+}, \lambda^{-})$;
  L.push$(\langle \lambda^{+}, \lambda^{-}, l \rangle)$;
  U.merge(S) ;
  /* Push $\boldsymbol{s}$ in Q only if it improves the Pareto frontier approximation */
  if $\boldsymbol{s} \neq \varnothing$ **then**
    Q.push$((\boldsymbol{s_1}, \boldsymbol{s}))$; Q.push$((\boldsymbol{s}, \boldsymbol{s_2}))$;
return L, U

Function GetCandidate$(S, \boldsymbol{s_1}, \boldsymbol{s_2}, \lambda^{+}, \lambda^{-})$

$\boldsymbol{s_{new}} := \emptyset$;
for $\boldsymbol{s} \in S$ **do**
  $select := True$
  if $\boldsymbol{s_{new}} \neq \emptyset \wedge \lambda^{+}E^{+}(\boldsymbol{s}) + \lambda^{-}E^{-}(\boldsymbol{s}) \geq \lambda^{+}E^{+}(\boldsymbol{s_{new}}) + \lambda^{-}E^{-}(\boldsymbol{s_{new}})$ **then**
    $select := False$
  $\vec{v_1} := \begin{pmatrix} -\lambda^{+} \\ -\lambda^{-} \end{pmatrix}$; $\vec{v_2} := \begin{pmatrix} E^{+}(\boldsymbol{s}) - E^{+}(\boldsymbol{s_1}) \\ E^{-}(\boldsymbol{s}) - E^{-}(\boldsymbol{s_1}) \end{pmatrix}$; $\vec{v_3} := \begin{pmatrix} E^{+}(\boldsymbol{s}) - E^{+}(\boldsymbol{s_2}) \\ E^{-}(\boldsymbol{s}) - E^{-}(\boldsymbol{s_2}) \end{pmatrix}$
  if $\langle \vec{v_1}, \vec{v_2} \rangle < 0 \vee \langle \vec{v_1}, \vec{v_3} \rangle < 0$ **then**
    $select := False$
  if $select$ **then**
    $\boldsymbol{s_{new}} := \boldsymbol{s}$
return $\boldsymbol{s_{new}}$

Algorithm 1: Approximation dichotomy algorithm to approximate a Pareto front.

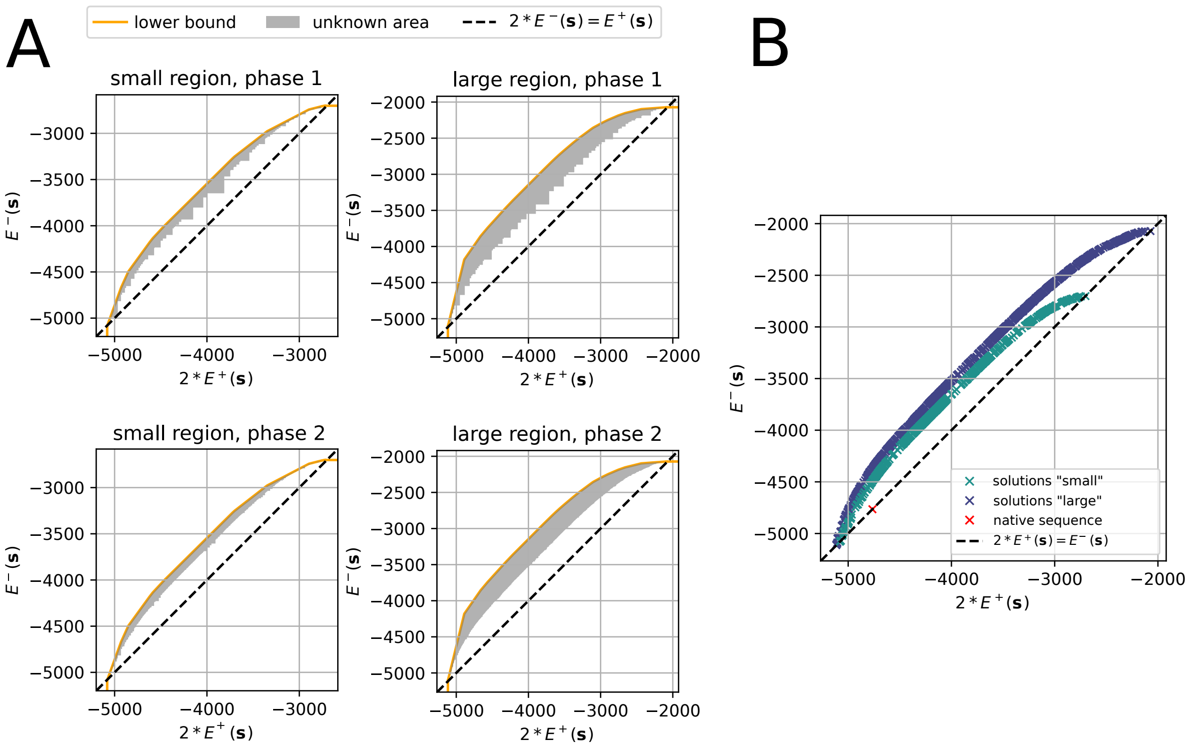

Figure S5. Comparison of the solutions in the two designable regions. A Bounds on the Pareto front of the problem for the two different designable regions and for the two phases of our method. The Pareto-optimal solutions are proven to be located in the grey area. **B** Superposition of the solutions computed for the two designable regions. Increasing the region size leads to solutions that are better on both objectives, more concentrated on the upper-left area (higher Effie cost).

2.2 Details and discussion on the in silico comparative analysis of EffieDes and ProteinMPNN on BMC-H designs

All predicted sequences by both Effie and ProteinMPNN were scored on the positive (AB) and negative hexameric states (AA and BB), with Effie and ProteinMPNN scoring functions, defining *pre-min* scores. Moreover, the hexameric structural conformations were constructed using Custozyme+ and minimized with Rosetta `beta_nov16` score function[30] and were then re-evaluated with both Effie and ProteinMPNN score functions, defining *post-min* scores.

The score differences $\Delta A = E_{\mathrm{AB}} - E_{\mathrm{AA}}$ and $\Delta B = E_{\mathrm{AB}} - E_{\mathrm{BB}}$ were computed for each design pre- and post-minimization and indicate the effectiveness of the design in satisfying the objective of favoring positive over negative states. The score difference $\Delta WT = E_{\mathrm{AB}} - E_{\mathrm{WT}}$ assess how far the positive state AB deviate from the wild-type homomeric RMM template. The normalized score differences $\bar{\Delta}A$ and $\bar{\Delta}B$ are represented on Figures 3B, S9,S8 and S10.

To assess the ability of EffieDes and ProteinMPNN in achieving our negative design objective, we calculated the averaged values $\overline{\Delta A}$ and $\overline{\Delta B}$ for each approach and we defined the vector $\overrightarrow{u} = \left(\overline{\Delta A}, \overline{\Delta B}\right)$: the further this vector extends in the direction $[-1, -1]$, the better. For each design method, we hence computed the scalar product on pre- and post-minimization scores:

$$\gamma = \overrightarrow{u} \cdot \overrightarrow{v_0}$$

with $\overrightarrow{v_0} = [-1, -1]$ (Figures 3B, S9, S8, S10 and S11). Table S2 provides γ values for all scoring evaluations with a higher γ indicating better design results.

Before minimization, the evaluation of EffieDes designs by ProteinMPNN scoring gives a $\gamma_{\mathrm{MPNN}} = 2.58$ and 3.16 on the small and large designable regions, respectively, while the evaluation of ProteinMPNN designs on the same regions gives lower $\gamma_{\mathrm{MPNN}} = 1.23$ and 1.65, respectively (Table S2 and Figures 3B, S8). Moreover, using Effie scoring, ProteinMPNN designs are considered even worse with negative γ_{Effie} for either designable region ($\gamma_{\mathrm{Effie}} = -0.40$ and -0.68 for the small and large regions, respectively). This implies that Effie scoring evaluates most of these sequence pairs as not meeting the design objectives.

After minimization, scores vary significantly, resulting in more scattered values for $\bar{\Delta}A$ and $\bar{\Delta}B$ in Figures S9 and S10, and, in all cases, $\gamma_{\text{post min}} < \gamma_{\text{pre min}}$ (see Table S2). This is consistent with the distortions of the hexamer conformations obtained for designs where the negative states are given a strong weight, resulting in high scores for states AA, BB, but also AB, and large differences $\bar{\Delta}A$ and $\bar{\Delta}B$ (Figures S14 and S15). For ProteinMPNN designs, scoring with Effie gives $\bar{\Delta}A$ and $\bar{\Delta}B$ that still tend to be positive post-minimization (Figures S9 and S10), with negative γ_{Effie} values and only a few designs displaying negative values for both $\bar{\Delta}A$ and $\bar{\Delta}B$ (less than ~ 10 % of designs for either region, as showed on Table S2).

ProteinMPNN scoring shows more designs with negative $\bar{\Delta}A$ and $\bar{\Delta}B$, but γ_{MPNN} of ProteinMPNN designs remains lower than γ_{MPNN} of EffieDes sequences (Table S2 and Figure S11). A possible explanation of the lesser degradation of ProteinMPNN scores after minimization lies in the different amounts of noise used during training between ProteinMPNN (0.2 Å std-dev) and Effie (0.14 Å std-dev), suggesting that using larger amounts of noise for Effie training could be useful.

Exploiting the ability of Effie scoring to separate the inter-chains component from the total score, $\Delta A_{\mathrm{inter}}$, $\Delta B_{\mathrm{inter}}$ and $\Delta WT_{\mathrm{inter}}$ were calculated and normalized. As above, the averaged values $\overline{\Delta A}$ and $\overline{\Delta B}$, as well as the scalar product γ_{inter} were used for comparison of both approaches. Thereby, we can see that the score differences between positive and negative states are mainly due to differences in inter-chain scores, as $\gamma_{\mathrm{inter}} \approx \gamma_{\mathrm{Effie}}$ (Tables S2 and S3). The post-minimization $\Delta WT_{\mathrm{inter}}$ score differences show that ProteinMPNN heteromeric AB designs scores are closer to the WT score than EffieDes designs (see Figures S12 and S13). However, in all cases, for the ProteinMPNN approach, $\gamma_{\mathrm{inter}} < 0$ which shows that ProteinMPNN designs rarely favor AB interactions over AA and BB interactions (see Table S3), failing to optimize the objective of our multi-state negative design.

All these scoring results show that under the specific design constraints induced by negative multi-state, autoregressive sampling clearly struggles to recover sequences satisfying the negative multi-state design objectives, while EffiDes seems better able to address this problem.

2.3 Supplementary material and methods for experimental characterization of BMC-H proteins

2.3.1 Cloning

DNA constructs for co-expression studies of Duo pairs in fusion to Flag peptide (Asp-Tyr-Lys-Asp-Asp-Asp-Asp-Lys) or His_6 tags, which sequences are given in Table S9, were directly cloned by Twist Bioscience between BglII and XhoI sites of their pET24 (+) (Kan^R) expression vector.

2.3.2 Protein expression, solubility and purification

The expression of proteins configured for the tripartite GFP assay (monomer A-GFP10 + monomer B-GFP11 + GFP1-9-His_6), or consisting instead of A-Flag and B-His_6 components was induced using the same protocol. After growing 1 mL of precultures overnight at 37 °C, 120 µL was inoculated in 12 mL of LBK. When the cultures reached mid-log phase ($OD_{600nm} = 0.6 - 0.8$), expression was induced with IPTG (200 µM final conc.). Incubation was continued for 4 hours before cell harvesting at 4,000 g, 4 °C. Supernatants were fully discarded and the pellets were gently resuspended in 0.8 mL of BugBuster (Novagen, amine free), supplemented with Benzonase (Proteogenix, $27\,U \cdot mL^{-1}$ final) and lysozyme ($20\,\mu g \cdot mL^{-1}$). The protease inhibitor phenylmethylsulfonyl fluoride was added immediately afterwards (PMSF, 1 mM final). After incubation at room temperature (RT) for 5 min, samples were transferred back to 4 °C before adding sodium phosphate (NaPi, 20 mM final, pH 8.1) and imidazole (10 mM). Total cellular content (TCC) fractions were then prepared from mixtures of 15 µL of these lysed samples and 45 µL of SDS loading dye (1.34X) and were denatured at 95 °C for 8 min. Insoluble debris and aggregates were removed by a 21,000 g centrifugation (10 min, 4 °C). Aliquots from resulting soluble fractions were denatured for sodium dodecylsulfate polyacrylamide gel electrophoresis (SDS-PAGE) analysis, as detailed for the TCC fraction. The remaining supernatant was loaded onto two Vivapure 8-96 well cobalt-chelate micro-columns (VivaScience) that had been preconditioned with Sol A (20 mM NaPi, 300 mM NaCl, 10 mM imidazole, pH 8.0). After 4×500 µL washing steps with Sol A, purified proteins were eluted with 300 µL of Sol B (Sol A plus 300 mM imidazole). EDTA was added (5 mM final conc.) and 60 µL of such purified fractions were denatured at 95 °C after mixing with 15 µL of SDS loading dye (5X). Analysis by SDS-polyacrylamide gel electrophoresis was typically performed on 17 % gels. Loaded volumes of TCC (typically 4 µL), soluble (4 µL), and purified (6 µL) fractions were identical for all compared samples. Protein bands were visualized with Coomassie Brilliant Blue R250 (BioRad).

2.3.3 Western blot analysis

After SDS-PAGE, gel contents were electro-transferred to a PVDF membrane (Immobilion-P, Milipore). Membranes were blocked at RT for 1 h in 5 % non-fat dry milk resuspended in TBS containing 0.05 % Tween 20. Subsequent treatments and washing steps followed standard protocols. The primary antibody immunolabelling was carried out with a mouse monoclonal anti-Flag antibody (MA1-91878, ThermoFisher), diluted at 1:2000 in the blocking solution. After incubation with a similarly diluted alkaline phosphatase-conjugated goat anti-mouse IgG (H+L) secondary antibody (31346, Fisher Scientific), and washing steps, blots were developed by reaction with Sigmafast BCIP/NBT substrate (typically for 3 to 5 min).

2.3.4 Size-exclusion chromatography

The protein oligomeric state was estimated by SEC using a Beckman Ultraspherogel SEC2000 column (7.5 × 300 mm) operated by a Waters 2690 HPLC separation module. After equilibration of the column in 25 mM NaPi, 150 mM NaCl, pH 7.5, each protein sample (30 µL) was injected and run at 1 µL/min flow rate. Elution was monitored at 280 nm with a Waters 996 Photodiode Array Detector (absorption). Elution volumes permitted to estimate the species molecular weight by comparison to calibration standards run under identical conditions: dextran blue (2 MDa), ferritin (440 kDa), aldolase (158 kDa), conalbumin (75 kDa), ovalbumin (43 kDa), carbonic anhydrase (35 kDa) and ribonuclease A (13.7 kDa).

Separation of Duo4 oligomeric species was carried out in a Superdex 200 Increase 10/300 GL column, operated by an Akta Pure Protein Purification System. About 300 µL of the corresponding cobalt-chelate-purified sample was injected and run in the column preconditioned with 10 mM Tris HCl, 200 mM NaCl, 0.5 mM EDTA, at pH 7.8. Protein-containing peaks were collected manually (280 nm detection). Fractions corresponding to the same peak were pooled together and concentrated 20 to 40-fold using Vivaspin 500 concentrator devices (10 kDa MWCO, PES). Resulting samples were reanalyzed by SEC-HPLC as indicated above.

2.4 Discussion on experimental characterization of BMC-H designs

2.4.1 Analysis of protein contents by SDS-PAGE

Although the expression levels of A-GFP10/B-GFP11 monomers and GFP1-9 were found to be adequate for the majority of the pairs, regardless of the design method, band intensities in soluble fractions appeared to decrease more often and more severely for ProteinMPNN designed pairs, as shown on Figure S16. These soluble fractions were prepared by centrifugation after cellular lysis, which induces the sedimentation of aggregated material, membranes, and other cellular debris. Aggregation of a fraction of the expressed BMC-H proteins therefore likely explains this decrease in band intensities mainly observed for ProteinMPNN designs. Of note, purification of a selection of cases using cobalt-based affinity chromatography proved that the two GFP10 and GFP11-tagged partners co-eluted in association to His_6-tagged GFP1-9 (Figure S17).

2.4.2 Copurification using Flag-peptide

Nine EffieDes and three ProteinMPNN designs were further investigated using a different experimental approach based on the attachment of a Flag peptide and the His_6 tag on monomers A and B from each pair, respectively (Figure S18A). The cross-association of both monomers from the Duo4, Duo11, and Duo13 was in that manner proven by the antibody-based detection of the Flag-carrying monomer co-purifying with the His_6-tagged monomer. Moreover, the western blot band intensities of these three designs are comparable or even higher than the ones detected for the positive control, consisting of a combination of Flag- and His_6- tagged WT RMM monomers (Figure S18B).

2.4.3 Size-exclusion chromatography

Duo4 yielding the most intense western blot band on Figure S18B, was characterized in more detail. Namely, after purification, its oligomeric state was investigated by size-exclusion chromatography (SEC), which revealed the existence of two preponderant species: one eluting at volumes expected for a hexamer and the second migrating as a dimer (Figure S19A). Some aggregated material was also detected. These two major peaks were collected separately from each other and, after reconcentration, they were re-injected in the SEC column under the same conditions, which preserved their respective elution volumes (*i.e.*, oligomeric state), as observed in Figure S19B. More importantly, each oligomeric state remained stable under the conditions of our experimental setup, ruling out equilibration between the two oligomeric species in solution. Therefore, we hypothesize that the formation of dimers (or aggregated species) might be the consequence of phenomena not directly related to the protein structural design problem, such as the difficulties of precisely tuning the expression levels and rates of each monomer inside the cells,[21] which could be regulated by using optimized genetic organizations.

2.5 Figures and Tables

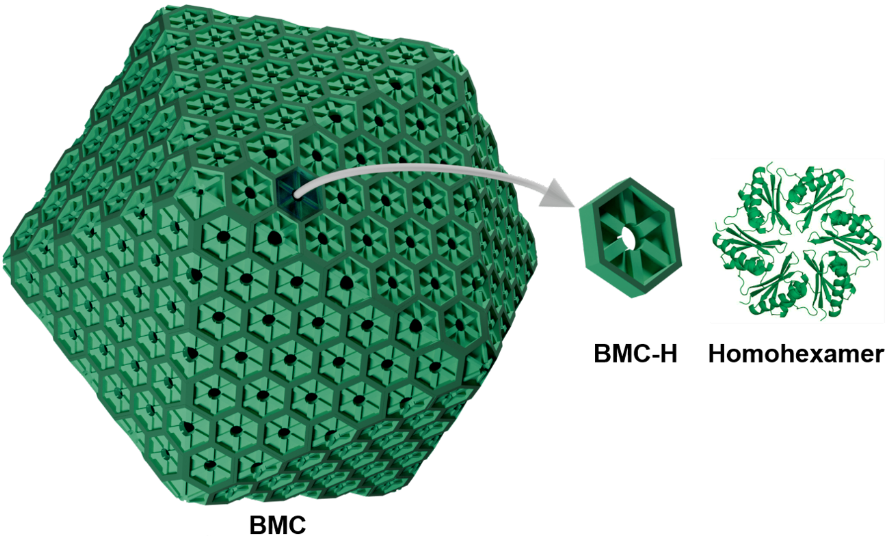

Figure S6. Representation of BMC-H homohexamer in a bacterial microcompartment (BMC) shell.

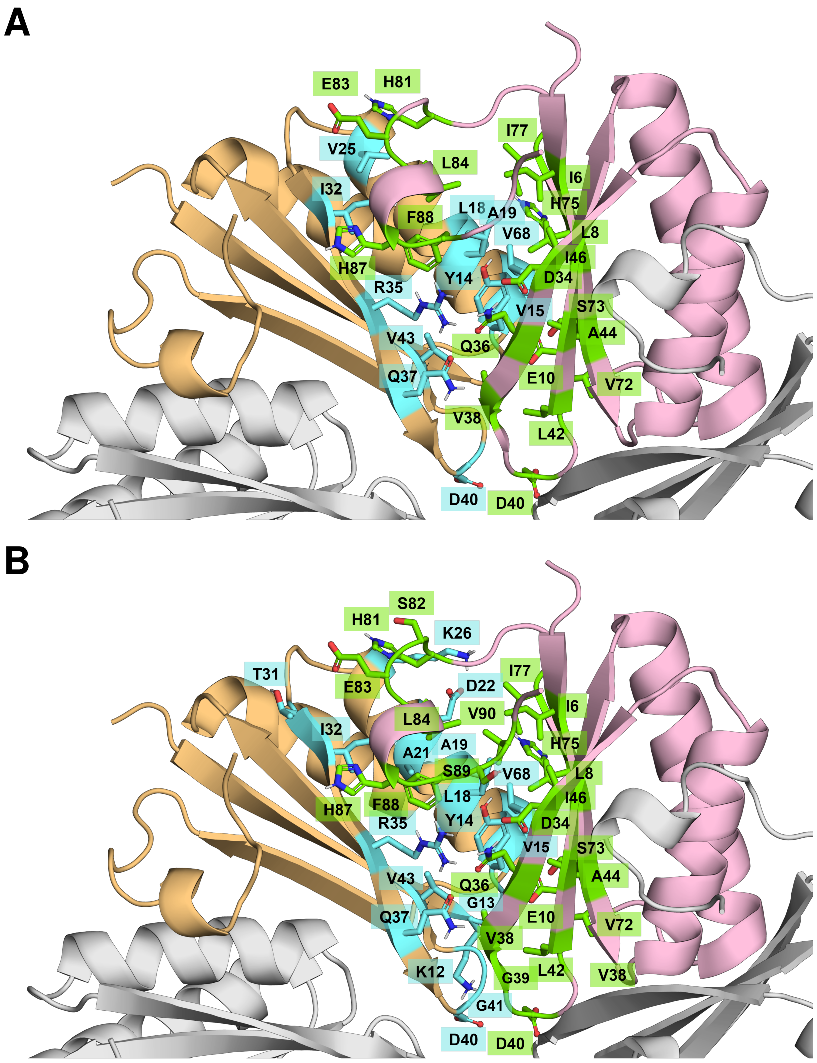

Figure S7. Interface between two monomers from WT RMM with the designable residues from the small (A) or the large region (B) displayed in sticks. The hexamer backbone is showed in cartoon representation with two subunits involved in one interface colored either in orange or in pink. Each side of the interface is colored either in cyan or in green with the side chains' atoms colored by type. For the small region (**A**), the first side of the interface is composed of **Y14, V15, L18, A19, V25, I32, R35, Q37, D40, V43 and V68**, and the second side of the interface of **I6, L8, E10, D34, Q36, V38, D40, L42, A44, I46, V72, S73, H75, I77, H81, E83, L84, H87 and F88**. For the large region (**B**), the first side of interface is composed of **K12, G13, Y14, V15, L18, A19, A21, D22, K26, T31, I32, R35, Q37, D40, G41, V43 and V68**, and the second side of the interface of **I6, L8, E10, D34, Q36, V38, G39, D40, L42, A44, I46, V72, S73, H75, I77, H81, S82, E83, L84, H87, F88, S89 and V90**.

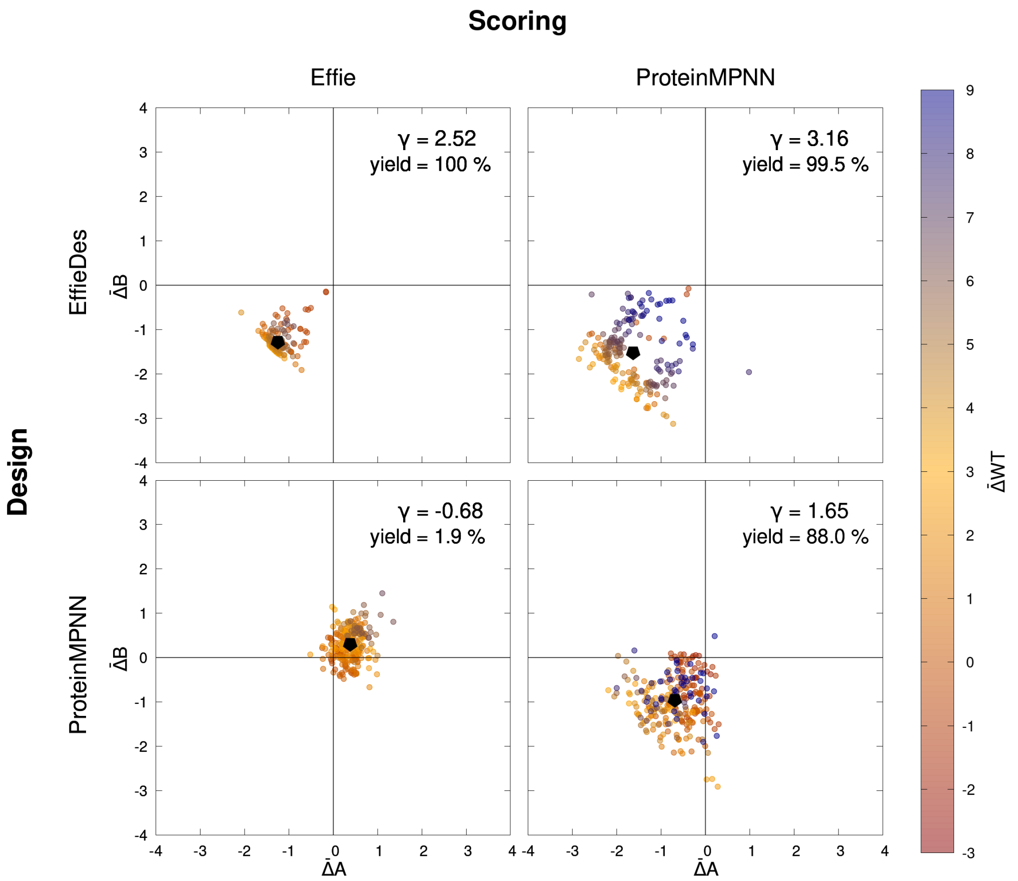

Figure S8. Comparison of pre-minimization score differences $\bar{\Delta}$A and $\bar{\Delta}$B for mutant sequences generated by EffieDes or ProteinMPNN on the large designable region. $\bar{\Delta}$A, $\bar{\Delta}$B and $\bar{\Delta}$WT correspond to the normalized values by the standard deviations over all ΔA and ΔB score differences from either Effie or ProteinMPNN scoring ($\sigma_{\text{Effie}} = 171.134$ and $\sigma_{\text{ProtMPNN}} = 64.1453$). Each dot represents a design with its color indicating the $\bar{\Delta}$WT score: blue colors indicate a likely weakly stable heterohexamer AB, compared to the WT homohexamer. The larger black dot corresponds to the averaged $\overline{\bar{\Delta}\text{A}}$ and $\overline{\bar{\Delta}\text{B}}$ used for the calculation of the γ values.

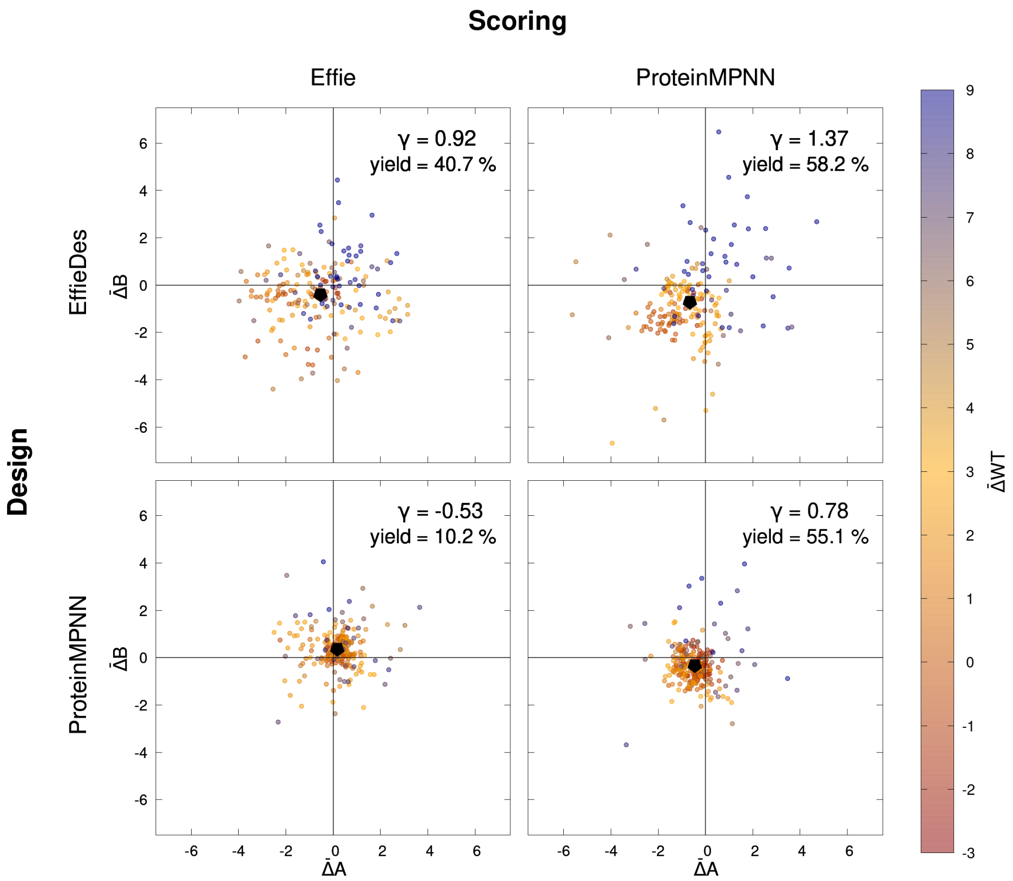

Figure S9. Comparison of the post-minimization score differences between positive and negative hexameric states for mutants generated by EffieDes or ProteinMPNN on the small designable region. $\bar{\Delta}$A, $\bar{\Delta}$B and $\bar{\Delta}$WT correspond to the normalized values by the standard deviations over all ΔA and ΔB score differences from either Effie or ProteinMPNN scoring ($\sigma_{\text{Effie}} = 171.134$ and $\sigma_{\text{ProtMPNN}} = 64.1453$). Each dot represents a design with its color indicating the $\bar{\Delta}$WT score: blue colors indicate a likely weakly stable heterohexamer AB, compared to the WT homohexamer. The larger black dot corresponds to the averaged $\overline{\bar{\Delta}\text{A}}$ and $\overline{\bar{\Delta}\text{B}}$ used for the calculation of the γ values.

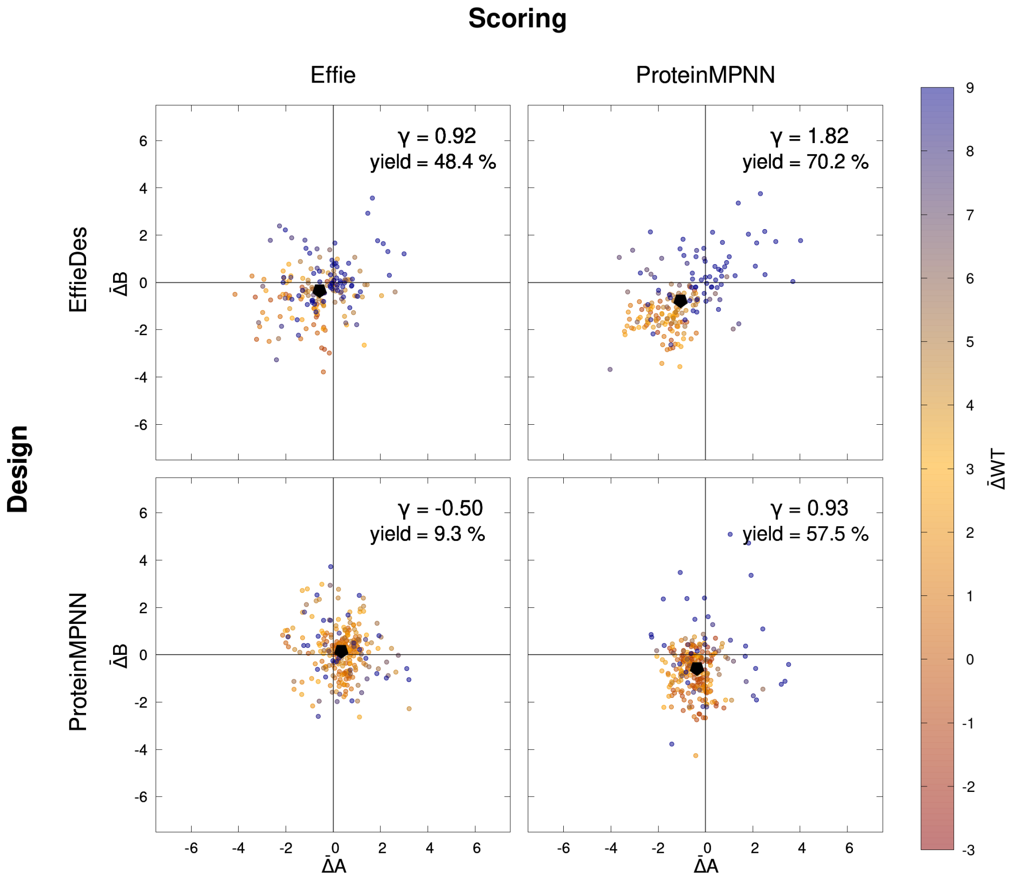

Figure S10. Comparison of post-minimization score differences $\bar{\Delta}$A and $\bar{\Delta}$B for mutant sequences generated by EffieDes or ProteinMPNN on the large designable region. $\bar{\Delta}$A, $\bar{\Delta}$B and $\bar{\Delta}$WT correspond to the normalized values by the standard deviations over all ΔA and ΔB score differences from either Effie or ProteinMPNN scoring ($\sigma_{\text{Effie}} = 171.134$ and $\sigma_{\text{ProtMPNN}} = 64.1453$). Each dot represents a design with its color indicating the $\bar{\Delta}$WT score: blue colors indicate a likely weakly stable heterohexamer AB, compared to the WT homohexamer. The larger black dot corresponds to the averaged $\overline{\bar{\Delta}\text{A}}$ and $\overline{\bar{\Delta}\text{B}}$ used for the calculation of the γ values.

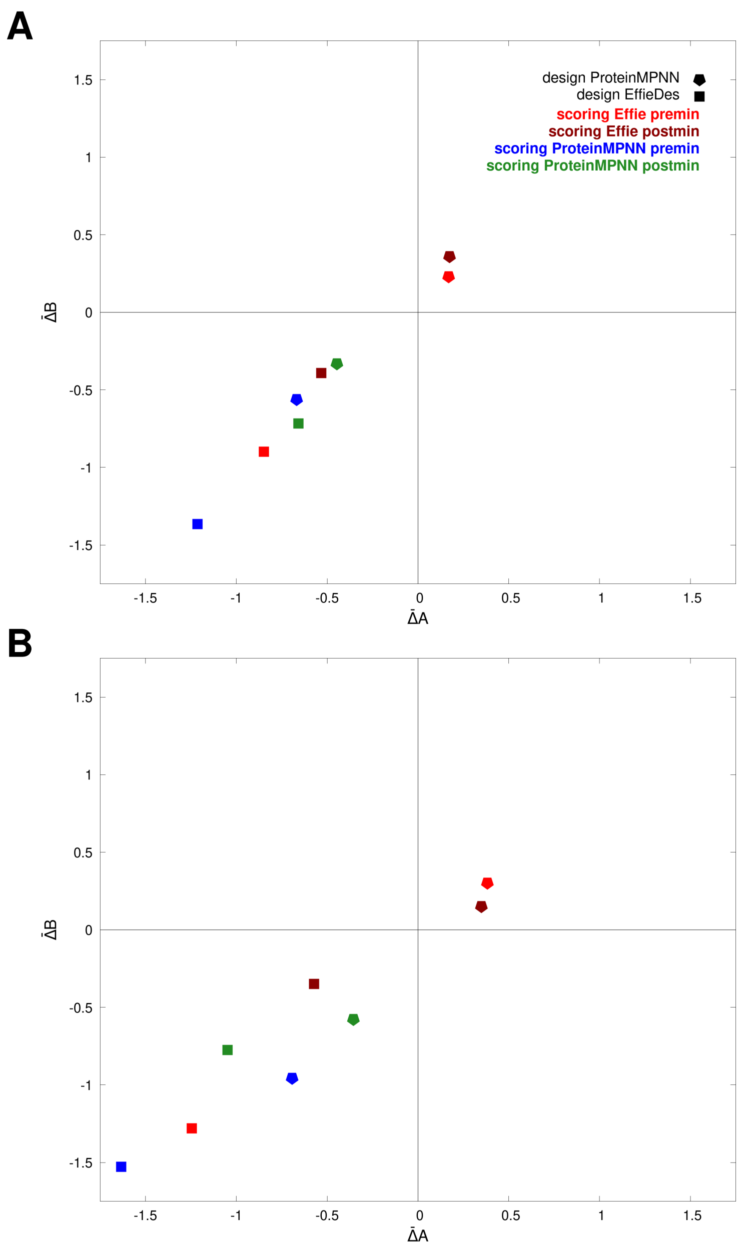

Figure S11. Comparison of averaged score differences $\overline{\Delta A}$ and $\overline{\Delta B}$ for (A) the small or (B) large designable region. Score differences obtained with Effie are shown in red or brown, and the ones obtained with ProteinMPNN are colored in blue or green. Averaged pre-minimization score differences are colored in red or blue and the post-minimization ones are in brown or green.

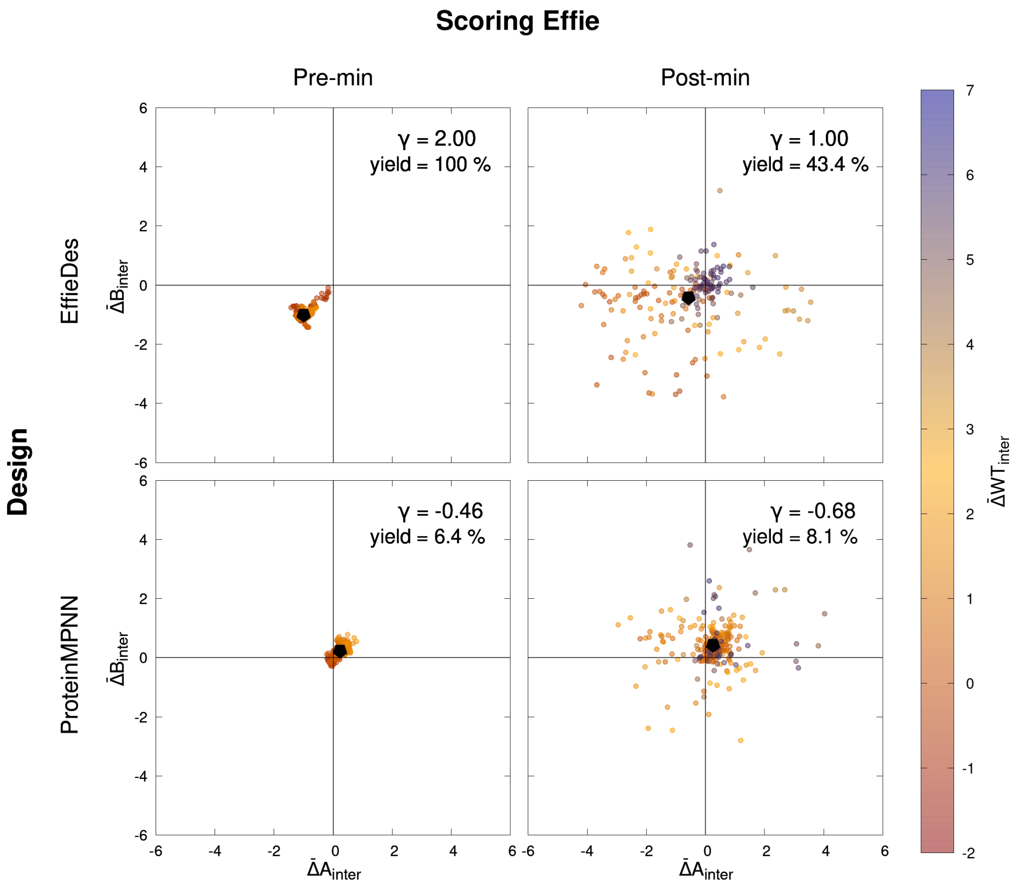

Figure S12. Comparison of Effie inter-chain scores for mutant sequences predicted either by EffieDes or ProteinMPNN on the small designable region, pre- (left) or post-minimization (right). $\bar{\Delta}A_{\text{inter}}$, $\bar{\Delta}B_{\text{inter}}$ and $\bar{\Delta}WT_{\text{inter}}$ correspond to the normalized values by the standard deviations over all ΔA_{inter} and ΔB_{inter} score differences ($\sigma_{\text{inter}} = 148.985$). Each dot represents a design with its color indicating the $\bar{\Delta}WT_{\text{inter}}$ score: blue colors indicate a likely weak interaction between monomers A and B, compared to the interaction between monomers in the WT homohexamer. The larger black dot corresponds to the averaged $\overline{\Delta A}_{\text{inter}}$ and $\overline{\Delta B}_{\text{inter}}$ used for the calculation of the γ_{inter} values.

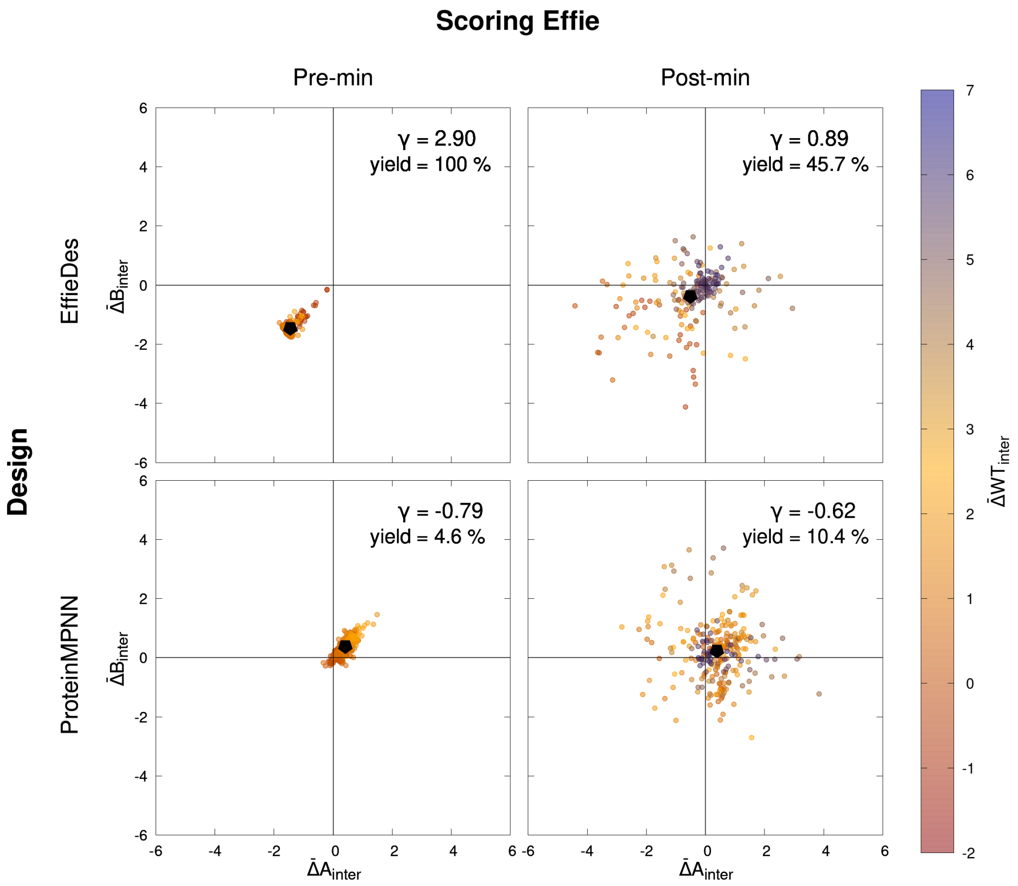

Figure S13. Comparison of Effie inter-chain scores for mutant sequences predicted either by EffieDes or ProteinMPNN on the large designable region, pre- (left) or post-minimization (right). $\bar{\Delta}A_{inter}$, $\bar{\Delta}B_{inter}$ and $\bar{\Delta}WT_{inter}$ correspond to the normalized values by the standard deviations over all ΔA_{inter} and ΔB_{inter} score differences (σ_{inter} = 148.985). Each dot represents a design with its color indicating the $\bar{\Delta}WT_{inter}$ score: blue colors indicate a likely weak interaction between monomers A and B, compared to the interaction between monomers in the WT homohexamer. The larger black dot corresponds to the averaged $\overline{\Delta A}_{inter}$ and $\overline{\Delta B}_{inter}$ used for the calculation of the γ_{inter} values.

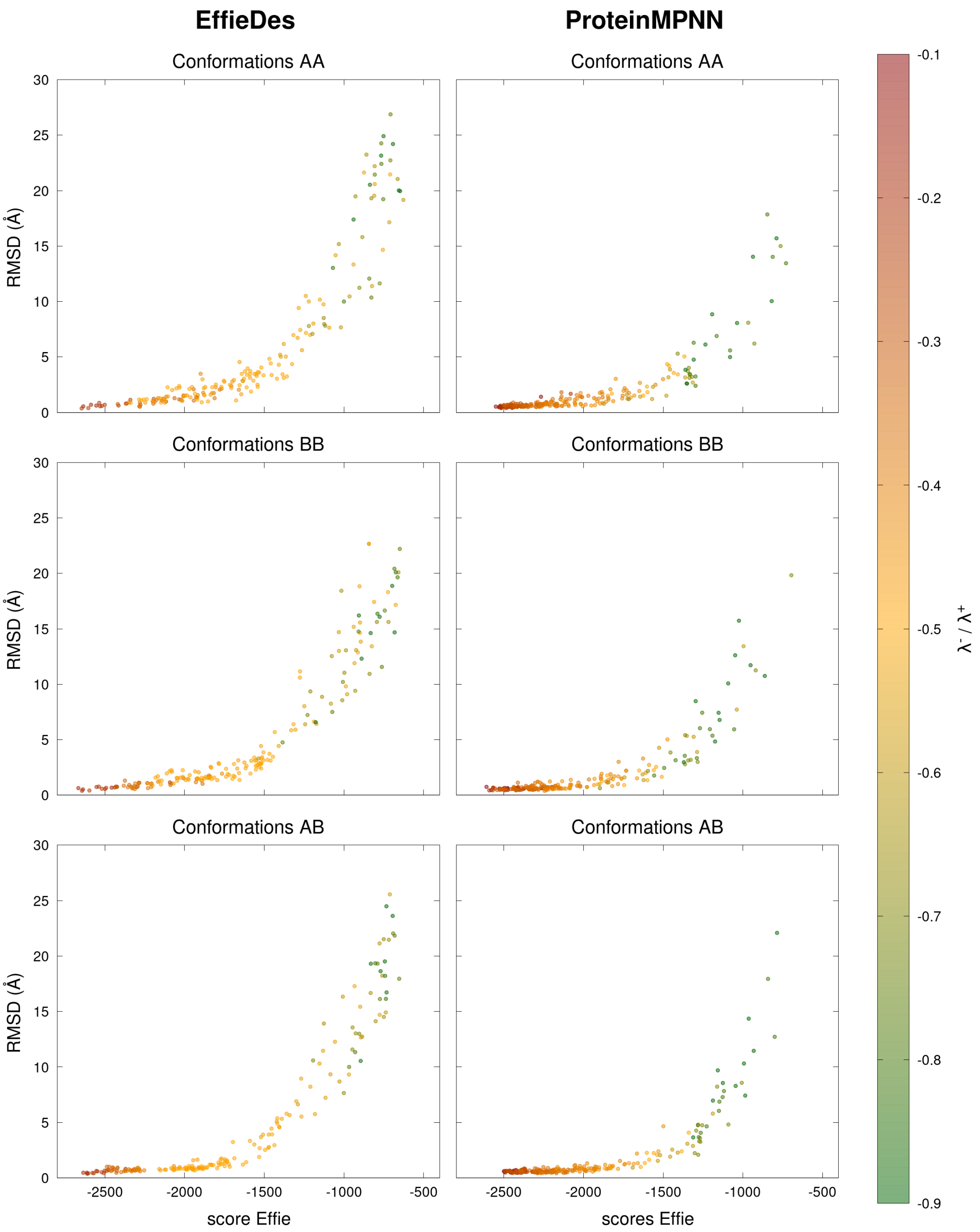

Figure S14. RMSD of the backbone of the hexamer of each state as a function of their score Effie for mutant sequences predicted on the small region by either EffieDes (left) or ProteinMPNN (right). Each dot represents a design and is colored according to the ratio $\frac{\lambda^-}{\lambda^+}$ with λ^+ the weight applied on the positive state and λ^- the weight applied on both negative states in each design approach.

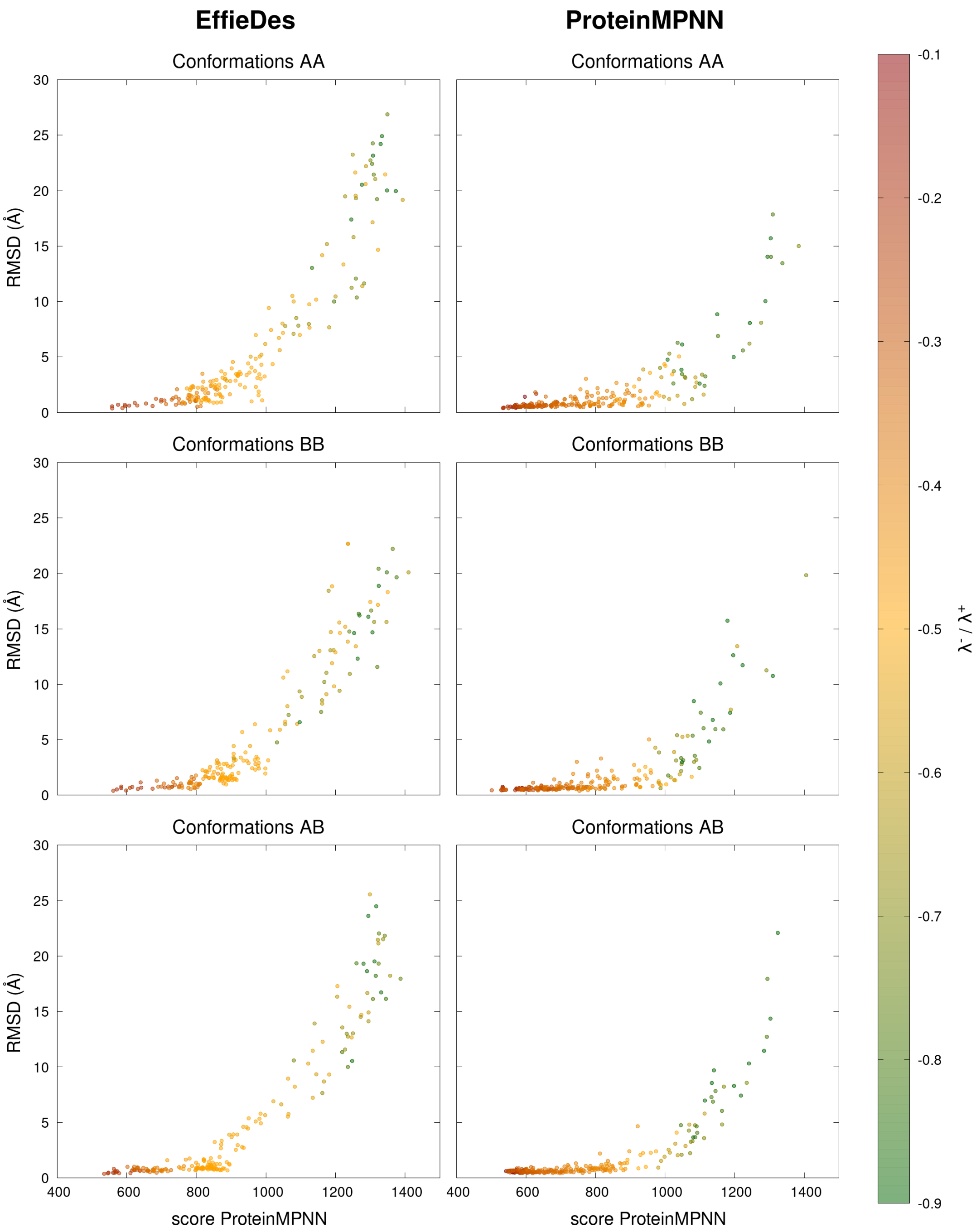

Figure S15. RMSD of the backbone of the hexamer of each state as a function of their ProteinMPNN score for mutant sequences predicted on the small region by either EffieDes (left) or ProteinMPNN (right). Each dot represents a design and is colored according to the ratio $\frac{\lambda^-}{\lambda^+}$ with λ^+ the weight applied on the positive state and λ^- the weight applied on both negative states in each design approach.

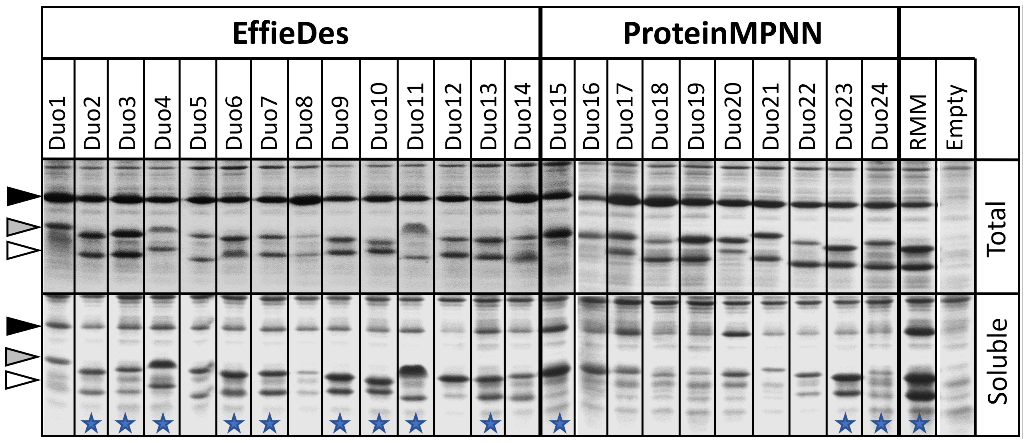

Figure S16. Expression and solubility of the Duo designs. Cells of emphE. coli overexpressing A-GFP10 and B-GFP11 monomers, plus the GFP1-9, were recovered 4 h post-induction. Total cellular contents (top) and proteins remaining soluble after lysis and centrifugation (bottom) were analyzed after thermal denaturation on SDS-polyacrylamide gels. The white, grey, and black arrows indicate the approximate positions of B-GFP11, A-GFP10 and GFP1-9, respectively. Selected cases indicated with a star were further investigated.

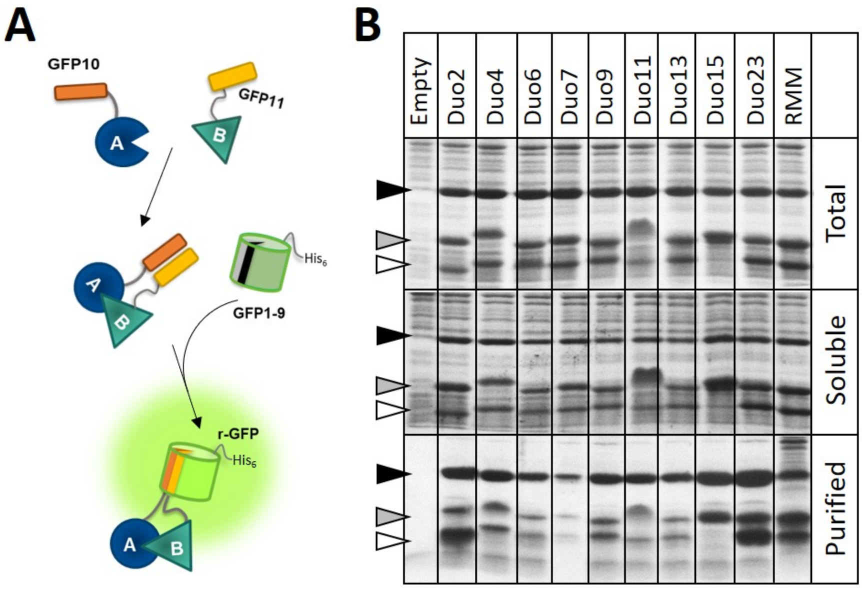

Figure S17. Experimental screening of the Duo designs using the tripartite GFP technology. (**A**) Schematic explanation of the tripartite GFP technology. An adapted GFP, composed of 11 β-strands, is split in 3 parts: the large GFP1-9 portion, plus the two terminal β-strands GFP10 and GFP11. The small strands are expressed in fusion with the two proteins to be assayed (A and B). For an interacting A/B pair, GFP10 and GFP11 are brought in close proximity, thus facilitating the reconstitution of a full GFP (r-GFP). On the contrary, when proteins A and B are not good interacting, the GFP reconstitution (encounter of the three parts) is entropically hampered. (**B**) Purification of selected Duo designs. Cells overexpressing each designed pair of A-GFP10 and B-GFP11 monomers, plus the His_6-tagged GFP1-9 component, were collected at the end of the culture. Corresponding total cellular contents were analyzed in Coomassie blue-stained polyacrylamide gels (Top). Fractions remaining soluble after lysis and centrifugation (middle) were loaded on cobalt-based chelating resins, with the intention to retain His_6-tagged GFP1-9 and any bound partner. The resulting purified fractions are shown in the bottom of the panel.

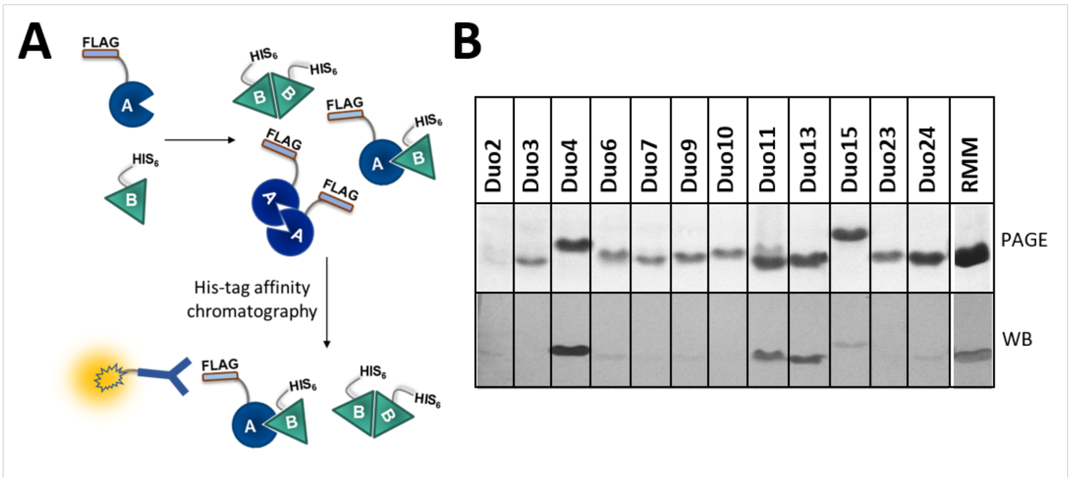

Figure S18. Verification of the interaction between both monomers A and B from selected Duo designs by coprecipitation. A) Scheme depicting the coprecipitation approach: monomer A is connected to a Flag peptide, whereas monomer B is fused to a His_6-purification tag. After overexpression and cellular lysis, soluble proteins carrying the purification tag are retained inside cobalt-based chelating resins. After washing, bound material is eluted from the resin and analyzed. **B)** Coomassie blue-stained polyacrylamide gels after coprecipitation (Top). Detection of co-purified A-FLAG partners by western blot, using Flag-specific antibodies (Bottom). For clarity, only the portion of the gel corresponding to the region of migration of the Duo monomers is shown.

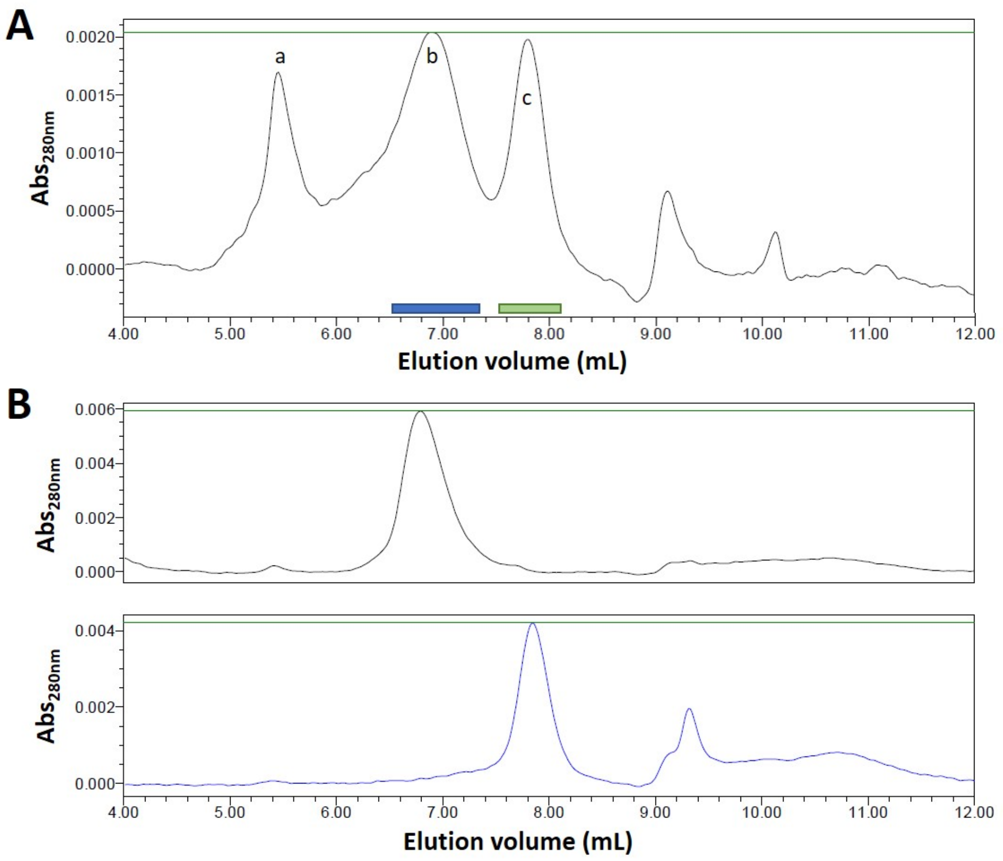

Figure S19. Characterization of the oligomeric state of Duo4. A. Size-exclusion chromatogram after injection of purified Duo4 on an analytical Beckman S2000 HPLC column. Shown is the profile of Duo4 absorption at 280 nm. Approximate molecular weights of 70 and 23 kDa were estimated for species eluting within peaks *b* and *c*, whereas the peak *a* likely consist of aggregated material (> 2 MDa). Elution at volumes higher than 9 mL would correspond to proteolysis (< 5 kDa) and/or small buffer components. **B.** Stability of isolated species displaying different oligomeric states. The purified Duo4 was injected in a Superdex 200 10/300 column (FPLC mode). This permitted to collect peaks *b* and *c* separately (the different fractions collected are indicated in panel A by blue and green bars). These fractions were concentrated and analyzed exactly as in panel A (on top for peak *a*, bottom for peak *b*).

Table S2. Evaluation of EffieDes or ProteinMPNN designs for each designable region.

	Design space	n	Scoring Effie pre-min γ	Scoring Effie pre-min yield	Scoring Effie post-min γ	Scoring Effie post-min yield	Scoring ProteinMPNN pre-min γ	Scoring ProteinMPNN pre-min yield	Scoring ProteinMPNN post-min γ	Scoring ProteinMPNN post-min yield
EffieDes	small	189	1.75	100 %	0.92	40.7 %	2.58	99.5 %	1.37	58.2 %
	large	188	2.52	100 %	0.92	48.4 %	3.16	99.5 %	1.82	70.2 %
ProteinMPNN	small	236	-0.40	3.0 %	-0.53	10.2 %	1.23	82.6 %	0.78	55.1 %
	large	259	-0.68	1.9 %	-0.50	9.3 %	1.65	88.0 %	0.93	57.5 %

(n) is the number of designs generated, (γ) indicates how much the positive state is improved over the negative states (see main text), (yield) gives the percentage of *in silico* successful designs, where the positive state gets a better score than both negative states.

Table S3. Evaluation of inter-chain Effie scores for EffieDes or ProteinMPNN designs for each designable region.

	Design space	n	Scoring Effie pre-min γ_{inter}	Scoring Effie pre-min yield	Scoring Effie post-min γ_{inter}	Scoring Effie post-min yield
EffieDes	small	189	2.00	100 %	1.00	43.4 %
	large	188	2.90	100 %	0.89	45.7 %
ProteinMPNN	small	236	-0.46	6.4 %	-0.68	8.1 %
	large	259	-0.79	4.6 %	-0.62	10.4 %

(n) is the number of designs generated, (γ_{inter}) indicates how much the positive state is improved over the negative states when considering only Effie inter-chain scores, (yield) gives the percentage of designs with both $\bar{\Delta}\text{A}_{\text{inter}} < 0$ and $\bar{\Delta}\text{B}_{\text{inter}} < 0$.

Table S4. Evaluation by scoring with Effie of the pairs of mutant sequences generated using either EffieDes or ProteinMPNN selected for experimental characterization.

	Scoring Effie						
	pre-min*			post-min			Mutant name
	ΔA	ΔB	ΔWT	ΔA	ΔB	ΔWT	
EffieDes	-32.3	-149.6	170.4	-80.4	-161.1	223.2	Duo1
	-60.4	-171.1	70.6	-223.4	-336.1	179.4	Duo2
	-157.0	-59.4	201.6	-813.5	-163.2	296.7	Duo3
	-73.4	-86.6	139.6	-107.6	-592.3	223.2	Duo4
	-146.4	-170.5	144.9	-611.9	-210.9	198.7	Duo5
	-59.1	-60.6	112.1	77.9	-300.3	130.4	Duo6
	-49.6	-128.8	60.1	-201.6	-204.4	43.1	Duo7
	-103.2	-147.8	35.2	-226.5	-327.4	97.9	Duo8
	4.9	-138.5	111.3	-103.6	-86.0	226.5	Duo9
	-30.3	-133.7	158.2	-138.7	-24.2	303.2	Duo10
	-76.4	-52.6	194.0	-134.7	-292.1	285.3	Duo11
	-25.7	-163.1	75.4	-129.6	-169.7	94.4	Duo12
	-51.6	-104.9	192.0	-444.8	-44.3	114.3	Duo13
	-60.1	-151.9	147.6	-486.8	-169.5	108.6	Duo14
ProteinMPNN	3.1	15.4	249.1	-45.5	-39.3	313.2	Duo15
	-30.1	55.7	134.6	120.3	51.0	172.1	Duo16
	14.2	52.9	304.5	-322.0	93.5	361.7	Duo17
	31.7	-5.7	270.0	35.8	-297.6	258.9	Duo18
	106.2	58.5	427.0	99.1	-266.7	465.3	Duo19
	93.1	42.0	495.2	81.2	232.7	849.3	Duo20
	24.3	73.1	400.2	-117.8	-8.5	611.4	Duo21
	-32.0	11.3	272.4	231.7	176.8	693.1	Duo22
	-4.5	-7.5	136.0	-332.4	-355.8	179.3	Duo23
	38.8	51.0	210.2	-189.5	100.3	208.1	Duo24

* Mutant sequence pairs were scored on the WT template conformation.

Table S5. Evaluation by scoring with ProteinMPNN of the pairs of mutant sequences generated using either EffieDes or ProteinMPNN selected for experimental characterization.

	Scoring ProteinMPNN						
	pre-min*			post-min			Mutant name
	ΔA	ΔB	ΔWT	ΔA	ΔB	ΔWT	
EffieDes	-45.1	-77.6	140.2	-82.0	-85.2	83.4	Duo1
	-53.2	-91.2	103.1	-60.7	-59.5	89.3	Duo2
	-141.5	-42.3	192.0	-239.5	-21.1	163.2	Duo3
	-21.6	-71.5	153.9	-37.1	-144.4	52.1	Duo4
	-92.0	-97.6	143.4	-142.7	-57.8	124.4	Duo5
	-45.6	-64.7	111.4	-59.8	-105.8	36.0	Duo6
	-67.5	-75.4	80.3	-64.6	-117.6	10.5	Duo7
	-98.8	-97.2	76.4	-96.2	-69.9	30.2	Duo8
	-10.9	-96.7	107.9	11.2	-74.4	32.7	Duo9
	-43.0	-94.9	154.9	-61.1	-65.8	46.4	Duo10
	-62.9	-56.2	156.3	-22.8	-98.8	116.9	Duo11
	-43.0	-109.6	94.2	-9.9	-126.1	28.4	Duo12
	-36.3	-59.3	172.2	-35.3	-92.2	71.0	Duo13
	-44.0	-86.0	137.0	-72.6	-87.3	76.0	Duo14
ProteinMPNN	-39.2	-32.9	125.3	-46.4	-73.9	92.5	Duo15
	-75.2	-29.5	47.5	-16.4	-29.9	37.4	Duo16
	-65.7	-45.9	105.1	-76.3	-44.0	104.3	Duo17
	-84.4	-76.2	152.6	-62.1	-82.1	107.2	Duo18
	-89.8	-68.5	202.0	-67.2	-92.2	133.8	Duo19
	-83.3	-100.8	254.0	-119.3	-51.3	214.8	Duo20
	-69.0	-115.4	213.9	-57.6	-56.6	194.3	Duo21
	-104.7	-40.0	118.9	-75.7	-68.4	93.3	Duo22
	-59.8	-79.2	22.5	-95.1	-142.8	-26.8	Duo23
	-42.1	-77.8	89.5	-64.5	-100.4	4.8	Duo24

* Mutant sequence pairs were scored on the WT template conformation.

Table S6. Evaluation by Effie inter-chain scores and RMSD to the WT hexamer template of mutant sequence pairs generated using either EffieDes or ProteinMPNN selected for experimental characterization.

	Scoring Effie						RMSD	Mutant name
	pre-min*			post-min				
	ΔA_{inter}	ΔB_{inter}	ΔWT_{inter}	ΔA_{inter}	ΔB_{inter}	ΔWT_{inter}		
EffieDes	-74.2	-107.1	2.6	-27.6	-95.9	123.1	0.99	Duo1
	-105.2	-127.4	-19.8	-208.4	-299.8	120.5	1.15	Duo2
	-114.1	-102.1	4.2	-696.6	-232.1	117.7	1.09	Duo3
	-87.1	-72.9	9.5	-116.2	-584.9	147.3	1.00	Duo4
	-166.9	-150.0	-33.8	-577.4	-225.4	54.8	1.05	Duo5
	-71.8	-47.2	-3.1	62.2	-242.4	97.3	0.63	Duo6
	-88.1	-90.3	-22.6	-181.2	-147.2	29.3	0.72	Duo7
	-130.7	-120.4	-50.5	-203.8	-314.5	67.7	0.96	Duo8
	-55.2	-78.5	16.4	-101.3	-43.2	248.5	0.90	Duo9
	-64.6	-99.5	8.0	-144.3	-88.5	239.7	0.98	Duo10
	-65.6	-63.8	33.8	-213.9	-247.0	164.1	1.16	Duo11
	-78.4	-110.1	-2.9	-123.1	-83.5	96.6	1.12	Duo12
	-71.1	-85.5	28.3	-408.1	-34.8	25.7	0.79	Duo13
	-96.1	-116.0	-0.6	-485.6	-105.7	60.9	0.97	Duo14
ProteinMPNN	-6.4	24.3	77.5	-36.0	22.5	234.7	0.80	Duo15
	-17.7	42.6	44.2	63.4	87.8	141.3	0.69	Duo16
	31.2	35.7	107.2	-270.3	81.4	211.7	0.72	Duo17
	40.4	-14.2	93.0	27.4	-297.5	148.0	0.76	Duo18
	82.6	81.0	201.3	151.3	-216.4	281.8	1.01	Duo19
	87.3	47.6	218.2	104.0	221.3	621.8	1.52	Duo20
	34.1	62.4	164.0	-103.0	-19.1	404.9	1.32	Duo21
	-28.5	6.4	76.3	251.4	251.0	604.1	2.01	Duo22
	-18.0	5.1	33.9	-347.6	-294.1	161.6	0.71	Duo23
	45.1	46.0	50.7	-215.8	87.5	167.5	0.66	Duo24

* Mutant sequence pairs were scored on the WT template conformation.

Table S7. Amino acid sequences of recombinant BMC-h proteins and number of incorporated mutations compared with WT RMM.

Protein	Nb mut.	Amino acids sequence
RMM (WT)		MSSNAIGLIETKGYVAALAAADAMVKAANVTITDRQQVGDGLVAVIVTGEVGAVKAATEAGAETASQVGELVSVHVIPRPHSELGAHFSVSSK
Duo1A	20	MSSNAIGAIQTKGTGAAIAAADAMVKAANVTLTSADTTGDGNVVVYVTGEVGAVKAATEAGAETASQDGELVAVYVTPRPHSELGAKRSVSSK
Duo1B	18	MSSNAIGIIITKGTVAADAAADAMVKAANVTDTIIDTTGDGNVLVLVTGEVGAVKAATEAGAETASQVGELLIVIVLPRPHSELGAVFSVSSK
Duo2A	18	MSSNAIGGIQTKGFAAALAAADAMVKAANVTVTAVVTTGDGEVKVYVTGEVGAVKAATEAGAETASQVGELLGVGVIPRPHSELGAIRSVSSK
Duo2B	19	MSSNAIGVITTKGFIAADAAADAMVKAANVTPTDLVTTGDGEVLVLVTGEVGAVKAATEAGAETASQLGELLTVVVLPRPHSELGAAFSVSSK
Duo3A	26	MSSNAKGAIQTKGWGAAIIAADAMIKAANVTLTSAKTTGGGNVAVYVTGEVGAVKAATEAGAETASQLGELVAVYVFPRPGSNHGAKRSVSSK
Duo3B	20	MSSNAIGIIITKGWVAADAAADAMEKAANVTDTDIKTTGGGNVLVLVTGEVGAVKAATEAGAETASQVGELLIVGVVPRPWSELGAVFSVSSK
Duo4A	25	MSSNAIGVIITKGFVAAVAAADAMVKAANVVLTSVYNTGDGQVLVLVTGEVGAVKAATEAGAETASQVGELLFVIVFPHPHEDLGAGADISSK
Duo4B	24	MSSNAIGLIATKGFGAALAAADAMVKAANVVGTPLYNTGDGQVVVFVTGEVGAVKAATEAGAETASQDGELVAVYVLPHPHEDLGAVLDISSK
Duo5A	32	MSSNAVGGIQTKGAGAAIGAADAMLKAANVVLTSAEVTGAGEVVVYVTGEVGAVKAATEAGAETASQGGELLAVAVIPHPLEIFGANRDISSK
Duo5B	28	MSSNALGIIITKGAVAADVAADAMGKAANVVGTSTEVTGAGEVLVLVTGEVGAVKAATEAGAETASQVGELLNVLVFPHPHEQLGAVFDISSK
Duo6A	17	MSSNAIGLISTKGFGAALAAADAMVKAANVTLTSGFNTGDGNVAVFVTGEVGAVKAATEAGAETASQAGELLAVHVLPRPHSELGAKLSVSSK
Duo6B	18	MSSNAIGVIVTKGFTAATAAADAMVKAANVTITSVFNTGDGNVLVLVTGEVGAVKAATEAGAETASQVGELYLVIVMPRPHSELGAIFSVSSK
Duo7A	18	MSSNAIGGIETKGAGAAIAAADAMVKAANVTLTDITNTGDGMVAVYVTGEVGAVKAATEAGAETASQAGELIAVAVFPRPHSELGATRSVSSK
Duo7B	17	MSSNAIGIITTKGAVAADAAADAMVKAANVTPTATTNTGDGMVLVLVTGEVGAVKAATEAGAETASQVGELINVIVIPRPHSELGAKFSVSSK
Duo8A	23	MSSNAVGGIETKGAGAAIAAADAMLKAANVTLTDITNTGGGMVAVYVTGEVGAVKAATEAGAETASQAGELIAVAVIPRPASIFGATRSVSSK
Duo8B	19	MSSNAIGIIVTKGAVAADVAADAMVKAANVTPTATTNTGGGMVGVLVTGEVGAVKAATEAGAETASQVGELINVIVIPRPHSELGAKFSVSSK
Duo9A	19	MSSNAIGLITTKGTGAALAAADAMVKAANVTVTSIKSSGDGNVTVFVTGEVGAVKAATEAGAETASQIGELLAVLVIPRPHSDLGAVLSVSSK
Duo9B	22	MSSNAIGVIVTKGTTAAVAAADAMVKAANVTLTSYKSSGDGNVLVTVTGEVGAVKAATEAGAETASQAGELIVVYVNPRPHSDLGAKASVSSK
Duo10A	25	MSSNAVGLITTKGIGAALDAADAMLKAANVTITSILSCGGGMCTVFVTGEVGAVKAATEAGAETASQIGELLAVLVLPRPSSTLGAVLSVSSK
Duo10B	26	MSSNAIGVIVTKGITAAVAAADAMTKAANVTLTSFLSCGGGMCLVTVTGEVGAVKAATEAGAETASQAGELLVVSVRPRPISQLGAKASVSSK
Duo11A	19	MSSNAIGIIITKGFVAAHAAADAMVKAANVTGTIIMTCGDGNVLVIVTGEVGAVKAATEAGAETASQVGELAYVGVLPRPHSDLGAVLSVSSK
Duo11B	21	MSSNAIGAIATKGFGAAIAAADAMVKAANVTLTAFMTCGDGNVVVYVTGEVGAVKAATEAGAETASQWGELVAVYVTPRPHSDLGAFDSVSSK
Duo12A	22	MSSNAVGLITTKGFGAALGAADAMVKAANVTITSIKNTGNGNVTVFVTGEVGAVKAATEAGAETASQAGELLAVAVLPRPHSDLGAVLSVSSK
Duo12B	23	MSSNAIGVIVTKGFTAAAVAADAMTKAANVTLTSAKNTGNGNVAVLVTGEVGAVKAATEAGAETASQVGELLIVLVFPRPHSQLGAKASVSSK
Duo13A	18	MSSNAIGLITTKGTGAALAAADAMVKAANVTVTVITKCGDGSVNVFVTGEVGAVKAATEAGAETASQIGELAAVLVIPRPHSELGAILSVSSK
Duo13B	21	MSSNAIGVIITKGTTAATAAADAMVKAANVTLTSITKCGDGSVLVLVTGEVGAVKAATEAGAETASQAGELIVVYVSPRPHSELGAAASVSSK
Duo14A	20	MSSNAIGGIQTKGGGAAIAAADAMVKAANVTVTGLRTTGDGEVVVYVTGEVGAVKAATEAGAETASQVGELLAVGVYPRPHSELGALRSVSSK
Duo14B	19	MSSNAIGIIITKGGVAADAAADAMVKAANVTGTSLRTTGDGEVLVLVTGEVGAVKAATEAGAETASQLGELLVVIVLPRPHSELGAAFSVSSK

Duo15A	17	MSSNAIGIIETKGVTAATAAADAMVKAANVTQTDFRSDGDGSVLVLVTGEVGAVKAATEAGAETASQKGELLIVRVIPRPHSELGAAFSVSSK
Duo15B	20	MSSNAIGQIKTKGMAAAIAAADAMVKAANVTETAVRSDGDGSVAVFVTGEVGAVKAATEAGAETASQGGELQEVTVDPRPHSELGAKWSVSSK
Duo16A	15	MSSNAIGIIETKGDTAANAAADAMVKAANVTITGRQQSGDGQVTVLVTGEVGAVKAATEAGAETASQVGELMTVTVKPRPHSELGAVVSVSSK
Duo16B	15	MSSNAIGQIETKGWAAAIAAADAMVKAANVTITNLQQSGDGQVRVNVTGEVGAVKAATEAGAETASQVGELLQVQVVPRPHSELGAAFSVSSK
Duo17A	22	MSSNAVGIIETKGNVAADFAADAMLKAANVTITDVQRSGNGSDTVIVTGEVGAVKAATEAGAETASQRGELLTVTVPPRPWSAIGACFSVSSK
Duo17B	23	MSSNAVGRIETKGFAAAMMAADAMYKAANVTSTSVQRSGNGSVTVVVTGEVGAVKAATEAGAETASQVGELFCVGVEPRPHSKLGAKRSVSSK
Duo18A	27	MSSNAVGGIQTLGEGAATAAADAMVQAANVKTTDMKDNGNGHVTVIVTGEVGAVKAATEAGAETASQAGELMQVAVIPRPNMDLGAYFAASSK
Duo18B	26	MSSNAIGIIETKGFVAAMCAADAMVDAANVKLTAVKDNGNGHVLVRVTGEVGAVKAATEAGAETASQVGELREVLVIPRPLDYLGARQGISSK
Duo19A	29	MSSNAIGGIATMRNTAALKALNAMVAAANVTITSIDRDGDSGSTVWVTGEVGAVKAATEAGAETASQVGELRGVGVLPRPNEALGAFFKVSSK
Duo19B	28	MSSNAIGAITTSGAVAAVMAGDAMVTAANVTMTNWDRDGDSGVTVLVTGEVGAVKAATEAGAETASQMGELQEVFVEPRPTSTLGAAAWVSSK
Duo20A	31	MSSNAMGGIETLTFAAAIMAEAAMVAAANVVITAVLNQGDADVKVWVTGEVGAVKAATEAGAETASQVGELKRVDVEPRPDNDLGAVFDFSSK
Duo20B	29	MSSNAVGIIETKGEVAADKAAPAMVRAANVLFTAKLNQGDADTCVIVTGEVGAVKAATEAGAETASQVGELRTVTVAPRPISPIGAKVCISSK
Duo21A	31	MSSNARGGIQTYSVSAALAAATAMVKAANVTLTAVHDSGNAEHTVAVTGEVGAVKAATEAGAETASQVGELRQVAVNPRPHTLVGANIRLSSK
Duo21B	30	MSSNAIGSITTWGFVAAYEAEDAMVRAANVAPTALHDSGNAEQCVLVTGEVGAVKAATEAGAETASQVGELITVVVEPRPASSLGAAFDYSSK
Duo22A	19	MSSNAVGGIFTKGYSAALGAADAMCKAANVTITDRQQDGEGLVSVKVTGEVGAVKAATEAGAETASQVGELLQVGVNPRPDSRNGAVLSVSSK
Duo22B	20	MSSNAIGVIETKGDLAAEWAADAMLKAANVTPTSYQQDGEGLVTVLVTGEVGAVKAATEAGAETASQVGELLEVFVIPRPDSVIGARFSVSSK
Duo23A	19	MSSNAIGIIETKGIVAAIAAADAMLKAANVTITATRNDGDGRVLVRVTGEVGAVKAATEAGAETASQVGELLNVGVIPRPDSTLGALLSVSSK
Duo23B	22	MSSNAVGMIQTKGEGAAVVAADAMVKAANVTLTHVRNDGDGRVTVVVTGEVGAVKAATEAGAETASQWGELLQVHVIPRPHSDLGATWSVSSK
Duo24A	22	MSSNAIGLITTKGAVAAMFAADAMLKAANVTPTSRQSTGDGMDTVFVTGEVGAVKAATEAGAETASQIGELLEVAVNPRPNSSLGARWSVSSK
Duo24B	23	MSSNACGAIQTKGPTAAVMAADAMLKAANVTLTDVQSTGDGMVVVIVTGEVGAVKAATEAGAETASQVGELIEVGVLPRPNSKSGAIWSVSSK

Table S8. BMC-H Duo DNA sequences for tripartite GFP assays. Fragment DNA sequences coding for A-GFP10 and B-GFP11 Duo monomers are provided with indication of flanking homology regions (blue lowercase letters) required for GIBSON-assembly of each pair of monomers with the NdeI/SalI open vector-1. NdeI and SalI sites are colored purple and red, respectively. The complete fragment sequence is given for Duo-1 monomers A and B, which are representative examples of GFP10 and GFP11-tagged organizations, respectively. Only the variable portion of the sequence, between NdeI and NotI sites (in blue), is given for all other Duo sequences. Receptor Vector-1 derives from a pET26b-vector with insertion of the indicated DNA portion between BglII and BlpI sites (yellow boxes). This vector codes for WT RMM-GFP10/RMM-GFP11 and His_6-tagged GFP1-9 and serves as a positive interaction control. Coding regions are in bold letters, the GFP10 sequence in light green and GFP11 in dark green.

Case	Sequence
VECTOR-1 (BglII/BlpI)	AGATCTCGATCCCGCGAAATTAATACGACTCACTATAGGGGAATTGTGAGCGGATAACAATTCCCCTCTAGAAATAagatttAAAtactttaagaagga<br>gatatacatATGAGTAGTAACGCGATTGGTTTAATTGAAACGAAAGGATACGTCGCCGCACTGGCTGCTGCAGATGCTATGGTAAAAGCTGCA<br>AATGTGACCATCACCGACCGGCAGCAGGTTGGCGATGGCTTAGTGGCAGTGATCGTAACGGGTGAGGTTGGGGCCGTAAAAGCTGCCACT<br>GAAGCAGGCGCTGAAACTGCGTCGCAGGTTGGCGAGCTGGTTAGCGTGCATGTTATCCCACGTCCCCATTCGGAACTCGGCGCACATTTTAG<br>CGTTAGCTCAAAAGGTGCGGCCGCATCAGAAGGAGGCGGTAGCGGGGGCCCTGGTTCGGGAGGGGAAGGTTCTGCTGGGGGAGGGAGCG<br>CTGGCGGGGGGTCTGATTTACCAGACGATCATTACCTGAGCACACAAACGATCCTTTCGAAAGACCTGAACGCAAGCTGAggatcaattgtttaaG<br>AAGGAGATATACCATGGCAAGTAGTAACGCGATTGGTTTAATTGAAACGAAAGGATACGTCGCCGCACTGGCTGCTGCAGATGCTATGGTA<br>AAAGCTGCAAATGTGACCATCACCGACCGGCAGCAGGTTGGCGATGGCTTAGTGGCAGTGATCGTAACGGGTGAGGTTGGGGCCGTAAAA<br>GCTGCCACTGAAGCAGGCGCTGAAACTGCGTCGCAGGTTGGCGAGCTGGTTAGCGTGCATGTTATCCCACGTCCCCATTCGGAACTCGGCGC<br>ACATTTTAGCGTTAGCTCAAAAGGATCCGCAGGCAGCGGTGGAAGTCCGGGTGGCGGTTCAGGCGGTAGCGGCAGCTCTGCGAGCGGCGG<br>CAGCACCAGCGAAAAACGCGATCACATGGTGCTGCTGGAATATGTGACCGCGGCGGGCATTACCGATGCGAGCTAATGACAAGTATGtcgact<br>cctaggaaagctttCTCGAGTTAACTCGTGAGCAATAACTAGCATAACCCCTTGGGGCCTCTAAACGGGTCTTGAGGGGTTTTTTGCTGAAAGTACA<br>CGGCCGCATAATCGAAATTAATACGACTCACTATAGGGGAATTGTGAGCGGATAACAATTCCCCTCTAGAATTAATTAAGTTTAACTTTAAGAA<br>GGAGATATACCTATGCGCAAAGGCGAAGAACTGTTTACCGGCGTGGTGCCGATTCTGATTGAACTGGATGGCGATGTGAACGGCCATAAAT<br>TTTTTGTGCGCGGCGAAGGCGAAGGCGATGCGACCATTGGCAAACTGAGCCTGAAATTTATTTGCACCACCGGCAAACTGCCGGTGCCGTG<br>GCCGACCCTGGTGACCACCCTGACCTATGGCGTGCAGTGCTTTAGCCGCTATCCGGATCACATGAAACGCCATGATTTTTTTAAAAGCGCGAT<br>GCCGGAAGGCTATGTGCAGGAACGCACCATTTATTTTAAAGATGATGGCACCTATAAAACCCGCGCGGAAGTGAAATTTGAAGGCGATACC<br>CTGGTGAACCGCATTGAACTGAAAGGCATTGATTTTAAAGAAGATGGCAACATTCTGGGCCATAAACTGGAATATAACTTTAACAGCCATAA<br>AGTGTATATTACCGCGGATAAACAGAACAACGGCATTAAAGCGAACTTTACCATTCGCCATAACGTGGAAGATGGCAGCGTGCAGCTGGCG<br>GATCATTATCAGCAGAACACCCCGATTGGCGATGGCCCGGTGCTGCTGCCGGATAACGGCAGCTCTGGTGCACATCACCATCACCATCATTA<br>AGCGGCAGCACTTGTTACCGGTCACCTCTCGAGAAAACGCGTCGAGAGCTGAG
Sequence of fragments coding for A monomers for GIBSON-assembly	
Duo-1A	agatttAAAtactttaagaaggagatatacatATGAGTAGTAACGCGATTGGTGCTATTCAGACGAAAGGAACCGGGGCCGCAATCGCTGCTGCAGAT<br>GCTATGGTAAAAGCTGCAAATGTGACCCTGACCAGCGCTGATACCACAGGCGATGGCAATGTGGTCGTGTACGTAACGGGTGAGGTTGGG<br>GCCGTAAAAGCTGCCACTGAAGCAGGCGCTGAAACTGCGTCGCAGGACGGCGAGCTGGTTGCGGTGTATGTTACCCCACGTCCCCATTCGG<br>AACTCGGCGCAAAACGTAGCGTTAGCTCAAAAGGTGCGGCCGCATCAGAAGGAGGCGGTAGCGGGGGCCCTGGTTCGGGAGGGGAAGGT<br>TCTGCTGGGGGAGGGAGCGCTGGCGGGGGGTCTGATTTACCAGACGATCATTACCTGAGCACACAAACGATCCTTTCGAAAGACCTGAACG<br>CAAGCTGATAAggatcaattgtttaa
Only given portion between NdeI and NotI for remaining sequences	
Duo-2A	catATGAGTAGTAACGCGATTGGTGGGATTCAGACGAAAGGATTCGCTGCCGCACTGGCTGCTGCAGATGCTATGGTAAAAGCTGCAAATGT<br>GACCGTGACCGCGGTAGTGACCACAGGCGATGGCGAGGTGAAGGTGTACGTAACGGGTGAGGTTGGGGCCGTAAAAGCTGCCACTGAAG<br>CAGGCGCTGAAACTGCGTCGCAGGTTGGCGAGCTGTTAGGTGTGGGCGTTATCCCACGTCCCCATTCGGAACTCGGCGCAATTCGTAGCGTT<br>AGCTCAAAAGGTGCGGCCGC
Duo-3A	catATGAGTAGTAACGCGAAGGGTGCTATTCAGACGAAAGGATGGGGGGCCGCAATTATCGCTGCAGATGCTATGATCAAAGCTGCAAATG<br>TGACCCTGACCAGCGCTAAAACCACAGGCGGCGGCAATGTGGCAGTGTACGTAACGGGTGAGGTTGGGGCCGTAAAAGCTGCCACTGAAG<br>CAGGCGCTGAAACTGCGTCGCAGTTAGGCGAGCTGGTTGCGGTGTATGTTTTCCCACGTCCCGGCTCGAACCACGGCGCAAAACGTAGCGTT<br>AGCTCAAAAGGTGCGGCCGC
Duo-4A	catATGAGTAGTAACGCGATTGGTGTAATTATTACGAAAGGATTCGTCGCCGCAGTGGCTGCTGCAGATGCTATGGTAAAAGCTGCAAATGT<br>GGTGCTGACCAGCGTATATAACACAGGCGATGGCCAAGTGTTGGTGCTGGTAACGGGTGAGGTTGGGGCCGTAAAAGCTGCCACTGAAGC<br>AGGCGCTGAAACTGCGTCGCAGGTTGGCGAGCTGTTATTCGTGATTGTTTTCCCACACCCCCATGAGGATCTCGGCGCAGGCGCGGACATCA<br>GCTCAAAAGGTGCGGCCGC
Duo-5A	catATGAGTAGTAACGCGGTGGGTGGGATTCAGACGAAAGGAGCGGGGGCCGCAATCGGCGCTGCAGATGCTATGTTGAAAGCTGCAAAT<br>GTGGTGCTGACCAGCGCTGAAGTGACAGGCGCGGGCGAGGTGGTCGTGTACGTAACGGGTGAGGTTGGGGCCGTAAAAGCTGCCACTGAA<br>GCAGGCGCTGAAACTGCGTCGCAGGGGGGCGAGCTGTTAGCGGTGGCGGTTATCCCACACCCCCTGGAGATTTTCGGCGCAAACCGTGACA<br>TCAGCTCAAAAGGTGCGGCCGC
Duo-6A	catATGAGTAGTAACGCGATTGGTTTAATTAGCACGAAAGGATTCGGGGCCGCACTGGCTGCTGCAGATGCTATGGTAAAAGCTGCAAATGT<br>GACCCTGACCAGCGGATTTAACACAGGCGATGGCAATGTGGCAGTGTTCGTAACGGGTGAGGTTGGGGCCGTAAAAGCTGCCACTGAAGC<br>AGGCGCTGAAACTGCGTCGCAGGCCGGCGAGCTGTTAGCGGTGCATGTTCTGCCACGTCCCCATTCGGAACTCGGCGCAAAACTGAGCGTTA<br>GCTCAAAAGGTGCGGCCGC

Duo-7A	catATGAGTAGTAACGCGATTGGTGGGATTGAAACGAAAGGAGCGGGGGCCGCAATCGCTGCTGCAGATGCTATGGTAAAAGCTGCAAATG<br>TGACCCTGACCGACATAACCAACACAGGCGATGGCATGGTGGCAGTGTACGTAACGGGTGAGGTTGGGGCCGTAAAAGCTGCCACTGAAG<br>CAGGCGCTGAAACTGCGTCGCAGGCCGGCGAGCTGATCGCGGTGGCGGTTTTCCCACGTCCCCATTCGGAACTCGGCGCAACCCGTAGCGTT<br>AGCTCAAAAGGTGCGGCCGC
Duo-8A	catATGAGTAGTAACGCGGTGGGTGGGATTGAAACGAAAGGAGCGGGGGCCGCAATCGCTGCTGCAGATGCTATGTTGAAAGCTGCAAAT<br>GTGACCCTGACCGACATAACCAACACAGGCGGCGGCATGGTGGCAGTGTACGTAACGGGTGAGGTTGGGGCCGTAAAAGCTGCCACTGAA<br>GCAGGCGCTGAAACTGCGTCGCAGGCCGGCGAGCTGATCGCGGTGGCGGTTATCCCACGTCCCGCGTCGATTTTCGGCGCAACCCGTAGCGT<br>TAGCTCAAAAGGTGCGGCCGC
Duo-9A	catATGAGTAGTAACGCGATTGGTTTAATTACCACGAAAGGAACCGGGGCCGCACTGGCTGCTGCAGATGCTATGGTAAAAGCTGCAAATGT<br>GACCGTGACCAGCATAAAAAGCAGCGGCGATGGCAATGTGACGGTGTTCGTAACGGGTGAGGTTGGGGCCGTAAAAGCTGCCACTGAAGC<br>AGGCGCTGAAACTGCGTCGCAGATCGGCGAGCTGTTAGCGGTGCTGGTTATCCCACGTCCCCATTCGGATCTCGGCGCAGTTCTGAGCGTTA<br>GCTCAAAAGGTGCGGCCGC
Duo-10A	catATGAGTAGTAACGCGGTGGGTTTAATTACCACGAAAGGAATTGGGGCCGCACTGGACGCTGCAGATGCTATGTTGAAAGCTGCAAATGT<br>GACCATCACCAGCATACTGAGCTGTGGCGGCGGCATGTGTACGGTGTTCGTAACGGGTGAGGTTGGGGCCGTAAAAGCTGCCACTGAAGCA<br>GGCGCTGAAACTGCGTCGCAGATCGGCGAGCTGTTAGCGGTGCTGGTTCTGCCACGTCCCAGCTCGACCCTCGGCGCAGTTCTGAGCGTTAG<br>CTCAAAAGGTGCGGCCGC
Duo-11A	catATGAGTAGTAACGCGATTGGTATCATTATTACGAAAGGATTCGTCGCCGCACATGCTGCTGCAGATGCTATGGTAAAAGCTGCAAATGT<br>GACCGGCACCATCATAATGACCTGTGGCGATGGCAATGTGTTGGTGATCGTAACGGGTGAGGTTGGGGCCGTAAAAGCTGCCACTGAAGCA<br>GGCGCTGAAACTGCGTCGCAGGTTGGCGAGCTGGCCTACGTGGGCGTTCTGCCACGTCCCCATTCGGATCTCGGCGCAGTTCTGAGCGTTAG<br>CTCAAAAGGTGCGGCCGC
Duo-12A	catATGAGTAGTAACGCGGTGGGTTTAATTACCACGAAAGGATTCGGGGCCGCACTGGGCGCTGCAGATGCTATGGTAAAAGCTGCAAATG<br>TGACCATCACCAGCATAAAAAACACAGGCAACGGCAATGTGACGGTGTTCGTAACGGGTGAGGTTGGGGCCGTAAAAGCTGCCACTGAAG<br>CAGGCGCTGAAACTGCGTCGCAGGCCGGCGAGCTGTTAGCGGTGGCGGTTCTGCCACGTCCCCATTCGGATCTCGGCGCAGTTCTGAGCGTT<br>AGCTCAAAAGGTGCGGCCGC
Duo-13A	catATGAGTAGTAACGCGATTGGTTTAATTACCACGAAAGGAACCGGGGCCGCACTGGCTGCTGCAGATGCTATGGTAAAAGCTGCAAATGT<br>GACCGTGACCGTGATAACCAAATGTGGCGATGGCTCAGTGAATGTGTTCGTAACGGGTGAGGTTGGGGCCGTAAAAGCTGCCACTGAAGC<br>AGGCGCTGAAACTGCGTCGCAGATCGGCGAGCTGGCCGCGGTGCTGGTTATCCCACGTCCCCATTCGGAACTCGGCGCAATTCTGAGCGTTA<br>GCTCAAAAGGTGCGGCCGC
Duo-14A	catATGAGTAGTAACGCGATTGGTGGGATTCAGACGAAAGGAGGCGGGGCCGCAATCGCTGCTGCAGATGCTATGGTAAAAGCTGCAAATG<br>TGACCGTGACCGGCCTTCGTACCACAGGCGATGGCGAGGTGGTCGTGTACGTAACGGGTGAGGTTGGGGCCGTAAAAGCTGCCACTGAAG<br>CAGGCGCTGAAACTGCGTCGCAGGTTGGCGAGCTGTTAGCGGTGGGCGTTTACCCACGTCCCCATTCGGAACTCGGCGCACTGCGTAGCGTT<br>AGCTCAAAAGGTGCGGCCGC
Duo-15A	catATGAGTAGTAACGCGATTGGTATCATTGAAACGAAAGGAGTGACTGCCGCAACCGCTGCTGCAGATGCTATGGTAAAAGCTGCAAATGT<br>GACCCAAACCGACTTCCGTAGCGACGGCGATGGCTCAGTGTTGGTGCTGGTAACGGGTGAGGTTGGGGCCGTAAAAGCTGCCACTGAAGCA<br>GGCGCTGAAACTGCGTCGCAGAAGGGCGAGCTGTTAATCGTGCGTGTTATCCCACGTCCCCATTCGGAACTCGGCGCAGCGTTTAGCGTTAG<br>CTCAAAAGGTGCGGCCGC
Duo-16A	catATGAGTAGTAACGCGATTGGTATCATTGAAACGAAAGGAGACACTGCCGCAAACGCTGCTGCAGATGCTATGGTAAAAGCTGCAAATGT<br>GACCATCACCGGCCGGCAGCAGAGCGGCGATGGCCAAGTGACGGTGCTGGTAACGGGTGAGGTTGGGGCCGTAAAAGCTGCCACTGAAGC<br>AGGCGCTGAAACTGCGTCGCAGGTTGGCGAGCTGATGACGGTGACCGTTAAACCACGTCCCCATTCGGAACTCGGCGCAGTGGTGAGCGTT<br>AGCTCAAAAGGTGCGGCCGC
Duo-17A	catATGAGTAGTAACGCGGTGGGTATCATTGAAACGAAAGGAAACGTCGCCGCAGATTTCGCTGCAGATGCTATGTTGAAAGCTGCAAATGT<br>GACCATCACCGACGTACAGCGTAGCGGCAACGGCTCAGACACGGTGATCGTAACGGGTGAGGTTGGGGCCGTAAAAGCTGCCACTGAAGC<br>AGGCGCTGAAACTGCGTCGCAGCGCGGCGAGCTGTTAACGGTGACCGTTCCGCCACGTCCCTGGTCGGCGATAGGCGCATGCTTTAGCGTTA<br>GCTCAAAAGGTGCGGCCGC
Duo-18A	catATGAGTAGTAACGCGGTGGGTGGGATTCAGACGCTGGGAGAGGGGGCCGCAACCGCTGCTGCAGATGCTATGGTACAGGCTGCAAATG<br>TGAAGACCACCGACATGAAAGATAATGGCAACGGCCACGTGACGGTGATCGTAACGGGTGAGGTTGGGGCCGTAAAAGCTGCCACTGAAG<br>CAGGCGCTGAAACTGCGTCGCAGGCCGGCGAGCTGATGCAAGTGGCGGTTATCCCACGTCCCAACATGGATCTCGGCGCATATTTTGCCGCG<br>AGCTCAAAAGGTGCGGCCGC
Duo-19A	catATGAGTAGTAACGCGATTGGTGGGATTGCGACGATGCGGAACACTGCCGCACTGAAGGCTTTAAACGCTATGGTAGCGGCTGCAAATGT<br>GACCATCACCAGCATAGATCGTGACGGCGATAGCGGGAGCACGGTGTGGGTAACGGGTGAGGTTGGGGCCGTAAAAGCTGCCACTGAAGC<br>AGGCGCTGAAACTGCGTCGCAGGTTGGCGAGCTGCGCGGTGTGGGCGTTCTGCCACGTCCCAACGAGGCGCTCGGCGCATTTTTTAAGGTTA<br>GCTCAAAAGGTGCGGCCGC
Duo-20A	catATGAGTAGTAACGCGATGGGTGGGATTGAAACGCTGACTTTCGCTGCCGCAATTATGGCTGAGGCGGCTATGGTAGCGGCTGCAAATGT<br>GGTGATCACCGCGGTACTGAACCAAGGCGATGCGGACGTGAAGGTGTGGGTAACGGGTGAGGTTGGGGCCGTAAAAGCTGCCACTGAAGC<br>AGGCGCTGAAACTGCGTCGCAGGTTGGCGAGCTGAAGCGCGTGGATGTTGAGCCACGTCCCGATAATGATCTCGGCGCAGTGTTTGACTTC<br>AGCTCAAAAGGTGCGGCCGC
Duo21-A	catATGAGTAGTAACGCGCGTGGTGGGATTCAGACGTATAGTGTGAGCGCCGCACTGGCTGCTGCAACCGCTATGGTAAAAGCTGCAAATGT<br>GACCCTGACCGCGGTACATGATAGCGGCAACGCGGAGCACACGGTGGCGGTAACGGGTGAGGTTGGGGCCGTAAAAGCTGCCACTGAAGC<br>AGGCGCTGAAACTGCGTCGCAGGTTGGCGAGCTGCGCCAAGTGGCGGTTAACCCACGTCCCCATACTCTGGTAGGCGCAAACATTCGCCTGA<br>GCTCAAAAGGTGCGGCCGC

Duo-22A	catATGAGTAGTAACGCGGTGGGTGGGATTTTTACGAAAGGATACAGCGCCGCACTGGGCGCTGCAGATGCTATGTGTAAAGCTGCAAATG<br>TGACCATCACCGACCGGCAGCAGGACGGCGAAGGCTTAGTGTCTGTGAAAGTAACGGGTGAGGTTGGGGCCGTAAAAGCTGCCACTGAAG<br>CAGGCGCTGAAACTGCGTCGCAGGTTGGCGAGCTGTTACAAGTGGGCGTTAACCCACGTCCCGATTCGCGTAATGGCGCAGTTCTGAGCGTT<br>AGCTCAAAAGGTGCGGCCGC
Duo-23A	catATGAGTAGTAACGCGATTGGTATCATTGAAACGAAAGGAATTGTCGCCGCAATCGCTGCTGCAGATGCTATGTTGAAAGCTGCAAATGT<br>GACCATCACCGCGACTCGTAACGACGGCGATGGCCGGGTGTTGGTGCGTGTAACGGGTGAGGTTGGGGCCGTAAAAGCTGCCACTGAAGC<br>AGGCGCTGAAACTGCGTCGCAGGTTGGCGAGCTGTTAAATGTGGGCGTTATCCCACGTCCCGATTCGACCCTCGGCGCACTGCTGTCCGTTA<br>GCTCAAAAGGTGCGGCCGC
Duo-24A	catATGAGTAGTAACGCGATTGGTTTAATTACCACGAAAGGAGCGGTCGCCGCAATGTTCGCTGCAGATGCTATGTTGAAAGCTGCAAATGT<br>GACCCCGACCAGCCGGCAGAGCACAGGCGATGGCATGGACACGGTGTTCGTAACGGGTGAGGTTGGGGCCGTAAAAGCTGCCACTGAAGC<br>AGGCGCTGAAACTGCGTCGCAGATCGGCGAGCTGTTAGAGGTGGCGGTTAACCCACGTCCCAACTCCAGCCTCGGCGCACGTTGGAGCGTT<br>AGCTCAAAAGGTGCGGCCGC
Sequence of fragments coding for B monomers for GIBSON-assembly	
Duo-1B	taaggatcaattgtttaagaaggagatatacatATGAGTAGTAACGCGATTGGTATCATTATTACGAAAGGAACCGTCGCCGCAGATGCTGCTGCAGAT<br>GCTATGGTAAAAGCTGCAAATGTGACCGACACCATCATAGATACCACAGGCGATGGCAATGTGTTGGTGCTGGTAACGGGTGAGGTTGGG<br>GCCGTAAAAGCTGCCACTGAAGCAGGCGCTGAAACTGCGTCGCAGGTTGGCGAGCTGTTAATCGTGATTGTTCTGCCACGTCCCCATTCGGA<br>ACTCGGCGCAGTGTTTAGCGTTAGCTCAAAAGGTGCGGCCGCAGGCAGCGGTGGCAGCCCGGGCGGCGGCAGCGGCGGCAGCGGCAGCA<br>GCGCGAGCGGCGGCAGCACCAGCGAAAAACGCGATCACATGGTGCTGCTGGAATATGTGACCGCGGCGGGCATTACCGATGCGAGCTAAT<br>GACAAGTATGtcgactcctaggaaagcttt
Only given portion between NdeI and NotI for remaining sequences	
Duo-2B	catATGAGTAGTAACGCGATTGGTGTAATTACCACGAAAGGATTCATCGCCGCAGATGCTGCTGCAGATGCTATGGTAAAAGCTGCAAATGT<br>GACCCCGACCGACCTTGTGACCACAGGCGATGGCGAGGTGTTGGTGCTGGTAACGGGTGAGGTTGGGGCCGTAAAAGCTGCCACTGAAGC<br>AGGCGCTGAAACTGCGTCGCAGTTAGGCGAGCTGTTAACGGTGGTGGTTCTGCCACGTCCCCATTCGGAACTCGGCGCAGCGTTTAGCGTTA<br>GCTCAAAAGGTGCGGCCGC
Duo-3B	catATGAGTAGTAACGCGATTGGTATCATTATTACGAAAGGATGGGTCGCCGCAGATGCTGCTGCAGATGCTATGGAGAAAGCTGCAAATGT<br>GACCGACACCGACATAAAAACCACAGGCGGCGGCAATGTGTTGGTGCTGGTAACGGGTGAGGTTGGGGCCGTAAAAGCTGCCACTGAAGC<br>AGGCGCTGAAACTGCGTCGCAGGTTGGCGAGCTGTTAATCGTGGGCGTTGTGCCACGTCCCTGGTCGGAACTCGGCGCAGTGTTTAGCGTTA<br>GCTCAAAAGGTGCGGCCGC
Duo-4B	catATGAGTAGTAACGCGATTGGTTTAATTGCGACGAAAGGATTCGGGGCCGCACTGGCTGCTGCAGATGCTATGGTAAAAGCTGCAAATGT<br>GGTGGGCACCCCGCTTTATAACACAGGCGATGGCCAAGTGGTCGTGTTCGTAACGGGTGAGGTTGGGGCCGTAAAAGCTGCCACTGAAGCA<br>GGCGCTGAAACTGCGTCGCAGGACGGCGAGCTGGTTGCGGTGTATGTTCTGCCACACCCCCATGAGGATCTCGGCGCAGTGCTGGACATCA<br>GCTCAAAAGGTGCGGCCGC
Duo-5B	catATGAGTAGTAACGCGCTGGGTATCATTATTACGAAAGGAGCGGTCGCCGCAGATGTCGCTGCAGATGCTATGGGGAAAGCTGCAAATG<br>TGGTGGGCACCAGCACTGAAGTGACAGGCGCGGGCGAGGTGTTGGTGCTGGTAACGGGTGAGGTTGGGGCCGTAAAAGCTGCCACTGAA<br>GCAGGCGCTGAAACTGCGTCGCAGGTTGGCGAGCTGTTAAATGTGCTGGTTTTCCCACACCCCCATGAGCAGCTCGGCGCAGTGTTTGACAT<br>CAGCTCAAAAGGTGCGGCCGC
Duo-6B	catATGAGTAGTAACGCGATTGGTGTAATTGTGACGAAAGGATTCACTGCCGCAACCGCTGCTGCAGATGCTATGGTAAAAGCTGCAAATGT<br>GACCATCACCAGCGTATTTAACACAGGCGATGGCAATGTGTTGGTGCTGGTAACGGGTGAGGTTGGGGCCGTAAAAGCTGCCACTGAAGCA<br>GGCGCTGAAACTGCGTCGCAGGTTGGCGAGCTGTACTTAGTGATTGTTATGCCACGTCCCCATTCGGAACTCGGCGCAATTTTTAGCGTTAGC<br>TCAAAAGGTGCGGCCGC
Duo-7B	catATGAGTAGTAACGCGATTGGTATCATTACCACGAAAGGAGCGGTCGCCGCAGATGCTGCTGCAGATGCTATGGTAAAAGCTGCAAATGT<br>GACCCCGACCGCGACTACCAACACAGGCGATGGCATGGTGTTGGTGCTGGTAACGGGTGAGGTTGGGGCCGTAAAAGCTGCCACTGAAGC<br>AGGCGCTGAAACTGCGTCGCAGGTTGGCGAGCTGATCAATGTGATTGTTATCCCACGTCCCCATTCGGAACTCGGCGCAAAATTTAGCGTTA<br>GCTCAAAAGGTGCGGCCGC
Duo-8B	catATGAGTAGTAACGCGATTGGTATCATTGTGACGAAAGGAGCGGTCGCCGCAGATGTCGCTGCAGATGCTATGGTAAAAGCTGCAAATGT<br>GACCCCGACCGCGACTACCAACACAGGCGGCGGCATGGTGGGTGTGCTGGTAACGGGTGAGGTTGGGGCCGTAAAAGCTGCCACTGAAGC<br>AGGCGCTGAAACTGCGTCGCAGGTTGGCGAGCTGATCAATGTGATTGTTATCCCACGTCCCCATTCGGAACTCGGCGCAAAATTTAGCGTTA<br>GCTCAAAAGGTGCGGCCGC
Duo-9B	catATGAGTAGTAACGCGATTGGTGTAATTGTGACGAAAGGAACCACTGCCGCAGTGGCTGCTGCAGATGCTATGGTAAAAGCTGCAAATGT<br>GACCCTGACCAGCTACAAAAGCAGCGGCGATGGCAATGTGTTGGTGACCGTAACGGGTGAGGTTGGGGCCGTAAAAGCTGCCACTGAAGC<br>AGGCGCTGAAACTGCGTCGCAGGCCGGCGAGCTGATCGTTGTGTATGTTAACCCACGTCCCCATTCGGATCTCGGCGCAAAAGCGAGCGTTA<br>GCTCAAAAGGTGCGGCCGC
Duo-10B	catATGAGTAGTAACGCGATTGGTGTAATTGTGACGAAAGGAATTACTGCCGCAGTGGCTGCTGCAGATGCTATGACTAAAGCTGCAAATGT<br>GACCCTGACCAGCTTCCTGAGCTGTGGCGGCGGCATGTGTTTGGTGACCGTAACGGGTGAGGTTGGGGCCGTAAAAGCTGCCACTGAAGCA<br>GGCGCTGAAACTGCGTCGCAGGCCGGCGAGCTGTTAGTTGTGAGCGTTCGTCCACGTCCCATTTCGCAGCTCGGCGCAAAAGCGAGCGTTAG<br>CTCAAAAGGTGCGGCCGC
Duo-11B	catATGAGTAGTAACGCGATTGGTGCTATTGCGACGAAAGGATTCGGGGCCGCAATCGCTGCTGCAGATGCTATGGTAAAAGCTGCAAATGT<br>GACCCTGACCGCGTTCATGACCTGTGGCGATGGCAATGTGGTCGTGTACGTAACGGGTGAGGTTGGGGCCGTAAAAGCTGCCACTGAAGCA<br>GGCGCTGAAACTGCGTCGCAGTGGGGCGAGCTGGTTGCGGTGTATGTTACCCCACGTCCCCATTCGGATCTCGGCGCATTTGATAGCGTTAG<br>CTCAAAAGGTGCGGCCGC

Duo-12B	catATGAGTAGTAACGCGATTGGTGTAATTGTGACGAAAGGATTCACTGCCGCAGCGGTCGCTGCAGATGCTATGACTAAAGCTGCAAATGTGACCCTGACCAGCGCTAAAAACACAGGCAACGGCAATGTGGCAGTGCTGGTAACGGGTGAGGTTGGGGCCGTAAAAGCTGCCACTGAAGCAGGCGCTGAAACTGCGTCGCAGGTTGGCGAGCTGTTAATCGTGCTGGTTTTCCCACGTCCCCATTCGCAGCTCGGCGCAAAAGCGAGCGTTAGCTCAAAAGGTGCGGCCGC
Duo-13B	catATGAGTAGTAACGCGATTGGTGTAATTATTACGAAAGGAACCACTGCCGCAACCGCTGCTGCAGATGCTATGGTAAAAGCTGCAAATGTGACCCTGACCAGCATAACCAAATGTGGCGATGGCTCAGTGTTGGTGCTGGTAACGGGTGAGGTTGGGGCCGTAAAAGCTGCCACTGAAGCAGGCGCTGAAACTGCGTCGCAGGCCGGCGAGCTGATCGTTGTGTATGTTAGCCCACGTCCCCATTCGGAACTCGGCGCAGCGGCGAGCGTTAGCTCAAAAGGTGCGGCCGC
Duo-14B	catATGAGTAGTAACGCGATTGGTATCATTATTACGAAAGGAGGCGTCGCCGCAGATGCTGCTGCAGATGCTATGGTAAAAGCTGCAAATGTGACCGGCACCAGCCTTCGTACCACAGGCGATGGCGAGGTGTTGGTGCTGGTAACGGGTGAGGTTGGGGCCGTAAAAGCTGCCACTGAAGCAGGCGCTGAAACTGCGTCGCAGTTAGGCGAGCTGTTAGTTGTGATTGTTCTGCCACGTCCCCATTCGGAACTCGGCGCAGCGTTTAGCGTTAGCTCAAAAGGTGCGGCCGC
Duo-15B	catATGAGTAGTAACGCGATTGGTCAAATTAAAACGAAAGGAATGGCTGCCGCAATCGCTGCTGCAGATGCTATGGTAAAAGCTGCAAATGTGACCGAGACCGCGGTACGTAGCGACGGCGATGGCTCAGTGGCAGTGTTCGTAACGGGTGAGGTTGGGGCCGTAAAAGCTGCCACTGAAGCAGGCGCTGAAACTGCGTCGCAGGGGGGCGAGCTGCAAGAGGTGACCGTTGACCCACGTCCCCATTCGGAACTCGGCGCAAAATGGAGCGTTAGCTCAAAAGGTGCGGCCGC
Duo-16B	catATGAGTAGTAACGCGATTGGTCAAATTGAAACGAAAGGATGGGCTGCCGCAATCGCTGCTGCAGATGCTATGGTAAAAGCTGCAAATGTGACCATCACCAATCTTCAGCAGAGCGGCGATGGCCAAGTGCGCGTGAATGTAACGGGTGAGGTTGGGGCCGTAAAAGCTGCCACTGAAGCAGGCGCTGAAACTGCGTCGCAGGTTGGCGAGCTGTTACAAGTGCAGGTTGTGCCACGTCCCCATTCGGAACTCGGCGCAGCGTTTAGCGTTAGCTCAAAAGGTGCGGCCGC
Duo-17B	catATGAGTAGTAACGCGGTGGGTCGCATTGAAACGAAAGGATTCGCTGCCGCAATGATGGCTGCAGATGCTATGTACAAAGCTGCAAATGTGACCAGCACCAGCGTACAGCGTAGCGGCAACGGCTCAGTGACGGTGGTGGTAACGGGTGAGGTTGGGGCCGTAAAAGCTGCCACTGAAGCAGGCGCTGAAACTGCGTCGCAGGTTGGCGAGCTGTTCTGTGTGGGCGTTGAGCCACGTCCCCATTCGAAACTCGGCGCAAAACGTAGCGTTAGCTCAAAAGGTGCGGCCGC
Duo-18B	catATGAGTAGTAACGCGATTGGTATCATTGAAACGAAAGGATTCGTCGCCGCAATGTGTGCTGCAGATGCTATGGTAGATGCTGCAAATGTGAAGCTGACCGCGGTAAAAGATAATGGCAACGGCCACGTGTTGGTGCGTGTAACGGGTGAGGTTGGGGCCGTAAAAGCTGCCACTGAAGCAGGCGCTGAAACTGCGTCGCAGGTTGGCGAGCTGCGCGAGGTGCTGGTTATCCCACGTCCCCTGGACTATCTCGGCGCACGTCAGGGTATCAGCTCAAAAGGTGCGGCCGC
Duo-19B	catATGAGTAGTAACGCGATTGGTGCTATTACCACGAGCGGAGCGGTCGCCGCAGTGATGGCTGGCGATGCTATGGTAACCGCTGCAAATGTGACCATGACCAACTGGGATCGTGACGGCGATAGCGGGGTGACGGTGCTGGTAACGGGTGAGGTTGGGGCCGTAAAAGCTGCCACTGAAGCAGGCGCTGAAACTGCGTCGCAGATGGGCGAGCTGCAAGAGGTGTTTGTTGAGCCACGTCCCACCTCGACCCTCGGCGCAGCGGCGTGGGTTAGCTCAAAAGGTGCGGCCGC
Duo-20B	catATGAGTAGTAACGCGGTGGGTATCATTGAAACGAAAGGAGAGGTCGCCGCAGATAAGGCTGCACCGGCTATGGTACGTGCTGCAAATGTGCTGTTCACCGCGAAGCTGAACCAAGGCGATGCGGACACATGTGTGATCGTAACGGGTGAGGTTGGGGCCGTAAAAGCTGCCACTGAAGCAGGCGCTGAAACTGCGTCGCAGGTTGGCGAGCTGCGCACGGTGACCGTTGCGCCACGTCCCATTTCGCCGATAGGCGCAAAAGTGTGTATCAGCTCAAAAGGTGCGGCCGC
Duo-21B	catATGAGTAGTAACGCGATTGGTAGTATTACCACGTGGGGATTCGTCGCCGCATACGAGGCTGAGGATGCTATGGTACGTGCTGCAAATGTGGCGCCGACCGCGCTTCATGATAGCGGCAACGCGGAGCAATGTGTGCTGGTAACGGGTGAGGTTGGGGCCGTAAAAGCTGCCACTGAAGCAGGCGCTGAAACTGCGTCGCAGGTTGGCGAGCTGATCACGGTGGTGGTTGAGCCACGTCCCGCGTCGAGCCTCGGCGCAGCGTTTGACTACAGCTCAAAAGGTGCGGCCGC
Duo-22B	catATGAGTAGTAACGCGATTGGTGTAATTGAAACGAAAGGAGACTTGGCCGCAGAATGGGCTGCAGATGCTATGTTGAAAGCTGCAAATGTGACCCCGACCAGCTACCAGCAGGACGGCGAAGGCTTAGTGACGGTGCTGGTAACGGGTGAGGTTGGGGCCGTAAAAGCTGCCACTGAAGCAGGCGCTGAAACTGCGTCGCAGGTTGGCGAGCTGTTAGAGGTGTTTGTTATCCCACGTCCCGATTCGGTGATAGGCGCACGTTTTAGCGTTAGCTCAAAAGGTGCGGCCGC
Duo-23B	catATGAGTAGTAACGCGGTGGGTATGATTCAGACGAAAGGAGAGGGGGCCGCAGTGGTCGCTGCAGATGCTATGGTAAAAGCTGCAAATGTGACCCTGACCCACGTACGTAACGACGGCGATGGCCGGGTGACGGTGGTGGTAACGGGTGAGGTTGGGGCCGTAAAAGCTGCCACTGAAGCAGGCGCTGAAACTGCGTCGCAGTGGGGCGAGCTGTTACAAGTGCATGTTATCCCACGTCCCCATTCGGATCTCGGCGCAACCTGGAGCGTTAGCTCAAAAGGTGCGGCCGC
Duo-24B	catATGAGTAGTAACGCGTGTGGTGCTATTCAGACGAAAGGACCGACTGCCGCAGTGATGGCTGCAGATGCTATGTTGAAAGCTGCAAATGTGACCCTGACCGACGTACAGAGCACAGGCGATGGCATGGTGGTCGTGATCGTAACGGGTGAGGTTGGGGCCGTAAAAGCTGCCACTGAAGCAGGCGCTGAAACTGCGTCGCAGGTTGGCGAGCTGATCGAGGTGGGCGTTCTGCCACGTCCCAACTCGAAATCAGGCGCAATTTGGAGCGTTAGCTCAAAAGGTGCGGCCGC

Table S9. BMC-H Duo DNA sequences for FLAG/His6 assays. Next DNA sequences were ordered directly cloned between BglII and XhoI sites of the pET24(+) vector (Kanamycin resistance) from Twist Biosciences. FLAG-tag is highlighted in dark blue, other details are the same as in Table S8.

Case	Sequence
Duo-2A/B	taatacgactcactataggggatctagatccgtatgacataaggaggtgaacatATGTCGTCGAACGCAATCGGTGGCATTCAGACTAAAGGTTTCGCTGCGGCACTCGCCGCAGCTGACGCCATGGTTAAAGCAGCCAATGTCACTGTGACTGCTGTCGTAACGACGGGTGACGGTGAGGTAAAGGTTTACGTTACCGGAGAGGTGGGCGCAGTTAAAGCTGCAACAGAGGCGGGAGCGGAAACGGCGAGCCAAGTTGGGGAGCTATTGGGCGTAGGAGTCATACCGCGGCCGCACTCGGAGTTGGGTGCCATAAGATCCGTTAGCTCAAAGGGGAGCGGAAGTGGGGCCGATTACAAAGATGACGATGACAAGTAAgtctgccaccctaagggggtcattgaATGAGCTCGAACGCTATCGGAGTAATAACGACAAAGGGCTTCATCGCGGCTGACGCTGCGGCGGATGCCATGGTGAAGGCTGCTAACGTGACTCCCACAGACTTAGTGACCACGGGTGATGGTGAAGTCCTAGTCTTGGTAACCGGAGAGGTCGGCGCCGTCAAGGCAGCAACGGAGGCAGGAGCCGAGACGGCCTCCCAATTAGGGGAGTTGCTGACTGTCGTTGTACTTCCCCGGCCGCATTCCGAACTCGGTGCAGCGTTCTCCGTTAGCTCAAAAGGTAGCGGAAGTGGTCATCACCACCACCATCACTAAtaacaaagctagcataaccccttggggcctctaaacgggtcttgaggggttttttg
Duo-3A/B	taatacgactcactataggggatctagagccccgaaccttagggaggtaaacatATGAGTTCTAATGCAAAGGGCGCCATTCAGACCAAAGGCTGGGGCGCGGCAATCATCGCGGCGGACGCCATGATTAAAGCTGCTAACGTCACATTAACATCCGCGAAGACAACTGGGGGCGGGAATGTTGCAGTGTACGTCACTGGCGAAGTGGGTGCGGTTAAAGCGGCCACAGAAGCGGGAGCAGAAACGGCAAGTCAGCTTGGAGAGCTTGTTGCAGTCTATGTGTTTCCCCGGCCCGGGAGTAACCACGGCGCTAAACGCTCAGTGAGCAGCAAGGGGTCAGGGTCCGGGGCCGATTACAAAGACGACGACGACAAATGAaatagacgggctaaggaggttcgacgATGAGTTCTAACGCGATCGGGATCATCATTACTAAAGGGTGGGTAGCAGCTGATGCTGCGGCGGACGCCATGGAAAAGGCGGCAAACGTTACTGACACGGACATAAAGACGACAGGCGGCGGGAACGTTTTAGTTTTAGTTACCGGAGAGGTGGGAGCAGTAAAGGCCGCTACAGAGGCCGGTGCTGAAACCGCGTCGCAGGTCGGTGAATTATTAATTGTCGGTGTCGTTCCGCGGCCGTGGTCAGAGCTGGGCGCTGTATTCAGCGTATCTTCTAAGGGCTCAGGTAGTGGGCATCATCACCACCATCACTAAtaacaaagctagcataaccccttggggcctctaaacgggtcttgaggggttttttg
Duo-4A/B	taatacgactcactataggggatctagaggaaaaacccctaaggagggacacatATGAGTTCTAATGCAATTGGAGTGATCATCACAAAAGGATTCGTAGCTGCGGTGGCCGCCGCTGATGCTATGGTCAAAGCAGCGAACGTGGTTTTAACTTCCGTGTATAACACTGGGGACGGGCAGGTCCTCGTATTAGTAACCGGTGAGGTCGGTGCAGTAAAGGCCGCGACGGAGGCGGGAGCAGAAACTGCGTCCCAAGTCGGTGAGTTATTGTTCGTAATCGTCTTCCCACATCCACACGAAGATCTCGGGGCCGGCGCTGACATTAGTTCCAAAGGCTCCGGAAGTGGAGCGGACTACAAAGACGATGATGATAAATGAcccgtcattgataaggaggtccaagtATGAGCTCGAATGCGATCGGATTGATCGCAACGAAAGGTTTCGGTGCGGCCTTGGCAGCAGCAGACGCCATGGTGAAGGCGGCTAACGTCGTTGGTACACCGTTGTACAACACTGGGGACGGACAAGTAGTTGTCTTTGTTACAGGCGAAGTAGGGGCTGTAAAGGCTGCCACAGAAGCAGGTGCGGAGACTGCATCCCAAGACGGTGAGCTCGTCGCCGTGTACGTGTTGCCTCACCCGCACGAGGACCTCGGAGCGGTACTGGACATTTCTTCGAAGGGGTCTGGTAGCGGACACCATCATCACCATCATTAAtaacaaagctagcataaccccttggggcctctaaacgggtcttgaggggttttttg
Duo-6A/B	taatacgactcactataggggatctagagagcccgggattaaggaggacggcatATGTCCAGCAACGCAATCGGGTTAATAAGCACGAAAGGTTTCGGCGCCGCACTGGCTGCCGCTGATGCTATGGTTAAAGCTGCGAATGTGACCCTGACCAGCGGATTTAATACAGGTGATGGTAATGTGGCAGTGTTCGTAACGGGCGAAGTTGGTGCCGTCAAAGCTGCAACTGAAGCCGGCGCTGAAACCGCGTCGCAGGCCGGCGAGCTGTTAGCGGTGCATGTTCTGCCACGCCCCCACTCTGAATTGGGCGCAAAACTGAGCGTGTCTAGTAAAGGATCGGGCAGCGGGGCCGATTATAAGGATGACGATGACAAGTGAggttcgctgaataaggaggtcagggcATGTCGAGCAACGCCATTGGGGTCATTGTCACTAAAGGCTTTACTGCCGCAACCGCCGCTGCAGATGCTATGGTAAAAGCTGCAAATGTGACCATCACCAGCGTATTTAACACAGGCGATGGCAATGTGTTGGTGCTGGTAACCGGTGAGGTGGGGGCGGTAAAGGCTGCCACGGAAGCAGGCGCAGAAACTGCGAGTCAAGTTGGCGAGCTGTACTTAGTGATTGTTATGCCGCGTCCTCATTCGGAACTCGGTGCAATTTTTAGCGTTAGCTCAAAGGGAAGCGGTAGCGGTCATCATCATCACCATCATTAAtaacaaagctagcataaccccttggggcctctaaacgggtcttgaggggttttttg
Duo-7A/B	taatacgactcactataggggatctagatccgtatgacataaggaggtgaacatATGTCGTCGAACGCAATCGGTGGCATTGAGACTAAAGGTGCGGGGGCCGCAATCGCTGCCGCTGATGCTATGGTTAAAGCTGCGAATGTGACCCTGACCGACATAACCAATACAGGTGATGGTATGGTGGCAGTGTACGTAACGGGCGAAGTTGGTGCCGTCAAAGCTGCAACTGAAGCCGGCGCTGAAACCGCGTCGCAGGCCGGCGAGCTGATCGCGGTGGCGGTTTTCCCACGCCCCCACTCTGAATTGGGCGCAACCCGTAGCGTGAGCTCAAAAGGCTCCGGAAGTGGAGCGGACTACAAAGACGATGATGATAAATGAcccgtcattgataaggaggtccaagtATGAGCTCGAATGCGATCGGAATCATCACAACGAAAGGTGCGGTCGCCGCAGACGCCGCTGCAGATGCTATGGTAAAAGCTGCAAATGTGACCCCGACCGCGACTACCAACACAGGCGATGGCATGGTGTTGGTGCTGGTAACCGGTGAGGTGGGGGCGGTAAAGGCTGCCACGGAAGCAGGCGCAGAAACTGCGAGTCAAGTTGGCGAGCTGATCAATGTGATTGTTATCCCGCGTCCTCATTCGGAACTCGGTGCAAAATTTAGCGTTAGCTCAAAGGGGTCTGGTAGCGGACACCATCATCACCATCATTAAtaacaaagctagcataaccccttggggcctctaaacgggtcttgaggggttttttg
Duo-9A/B	taatacgactcactataggggatctagagagcccgggattaaggaggacggcatATGTCCAGCAACGCAATCGGGTTAATAACCACGAAAGGTACAGGCGCCGCGCTTGCAGCCGCAGATGCGATGGTCAAAGCGGCTAACGTGACAGTAACGAGCATTAAGTCCTCAGGGGATGGTAATGTGACAGTCTTCGTAACGGGTGAAGTTGGGGCCGTTAAGGCTGCAACTGAGGCAGGAGCTGAGACTGCCTCCCAGATTGGAGAGTTGCTTGCTGTCTTGGTTATCCCTAGACCGCACTCAGACCTGGGCGCAGTTCTAAGTGTCTCTAGTAAAGGATCGGGCAGCGGGGCCGATTATAAGGATGACGATGACAAGTGAggttcgctgaataaggaggtcagggcATGTCGAGCAACGCCATTGGGGTCATTGTCACTAAAGGCACCACAGCTGCCGTCGCTGCCGCGGATGCCATGGTCAAGGCAGCCAATGTGACCTTGACTTCCTACAAGTCTAGCGGAGACGGAAACGTCTTAGTGACAGTGACCGGAGAAGTGGGAGCCGTGAAAGCAGCCACTGAGGCTGGTGCCGAGACCGCAAGCCAAGCCGGTGAGCTGATCGTAGTCTACGTGAATCCAAGACCTCATAGTGATCTAGGCGCCAAAGCTTCAGTGTCTAGCAAGGGAAGCGGTAGCGGTCATCATCATCACCATCATTAAtaacaaagctagcataaccccttggggcctctaaacgggtcttgaggggttttttg
Duo-10A/B	taatacgactcactataggggatctagagacgagggagataaggaggcgtgcatATGAGCAGCAACGCTGTGGGGTTGATAACTACCAAGGGAATAGGAGCGGCATTAGACGCAGCTGACGCCATGCTGAAGGCCGCGAACGTTACCATTACGTCTATTCTCAGCTGCGGTGGTGGTATGTGTACGGTGTTCGTTACCGGAGAAGTCGGAGCGGTAAAGGCCGCTACAGAAGCAGGAGCTGAAACTGCTAGTCAGATTGGAGAGCTGTTAGCTGTTTTAGTTTTACCTCGTCCCTCCAGCACCCTCGGAGCCGTTCTAAGCGTGAGCTCCAAGGGGAGTGGATCCGGCGCAGACTATAAAGATGATGATGATAAGTAAgaaaagaagtaaaaggaggcagatacATGAGCTCAAACGCTATAGGCGTAATCGTAACAAAAGGAATCACGGCGGCCGTCGCAGCGGCGGATGCCATGACCAAGGCGGCCAACGTTACCTTGACTAGCTTCTTAAGCTGCGGCGGTGGAATGTGTCTGGTGACGGTTACAGGTGAGGTTGGGGCCGTCAAAGCGGCTACCGAGGCCGGTGCCGAGACAGCGTCACAGGCTGGAGAGCTTCTAGTCGTCAGCGTAAGACCGCGTCCTATCAGTCAGCTTGGGGCCAAAGCTAGTGTGTCTAGCAAAGGTAGTGGTTCGGGCCACCATCATCACCATCATTGAtaacaaagctagcataaccccttggggcctctaaacgggtcttgaggggttttttg

Duo-11A/B	taatacgactcactataggggatctagagaacgagtaatccaggaggtctgcatATGAGCTCTAATGCTATTGGGATCATAATCACCAAGGGCTTTGTCGCCGC<br>ACATGCCGCAGCTGATGCAATGGTGAAGGCGGCGAATGTCACGGGCACGATCATTATGACCTGCGGTGACGGAAACGTGTTAGTGATCGT<br>TACCGGAGAAGTAGGCGCAGTGAAGGCCGCTACCGAGGCTGGCGCAGAGACTGCTTCGCAAGTCGGGGAGCTAGCCTATGTGGGAGTTT<br>TACCTCGGCCTCACTCAGACTTGGGCGCGGTACTATCAGTGTCATCAAAGGGGTCTGGTAGTGGCGCCGACTACAAAGATGACGACGACA<br>AGTAAagtcagagcggtaaggaggtccctttATGAGCAGTAACGCTATAGGTGCTATAGCTACAAAAGGCTTTGGAGCAGCAATTGCGGCGGCTGA<br>CGCCATGGTCAAGGCGGCCAACGTAACGCTCACGGCGTTTATGACCTGCGGGGACGGAAATGTAGTTGTGTATGTGACTGGTGAGGTAGG<br>TGCGGTAAAAGCCGCCACTGAGGCTGGGGCAGAAACAGCGTCCCAGTGGGGAGAGCTCGTAGCGGTATACGTGACACCCCGCCCGCATTC<br>AGATCTCGGTGCCTTCGACAGCGTCTCATCCAAGGGCAGTGGCAGTGGGCATCACCACCACCACCATTAAtaacaaagctagcataaccccttgggg<br>cctctaaacgggtcttgaggggttttttg
Duo-13A/B	taatacgactcactataggggatctagagagcccgggattaaggaggacggcatATGTCCAGCAACGCAATCGGGTTAATAACCACGAAAGGTACAGGCGCCG<br>CGCTTGCTGCCGCTGATGCTATGGTTAAAGCTGCGAATGTGACCGTGACCGTGATAACCAAGTGTGGTGATGGTTCAGTGAATGTGTTCGT<br>AACGGGCGAAGTTGGTGCCGTCAAAGCTGCAACTGAAGCCGGCGCTGAAACCGCGTCGCAGATCGGCGAGCTGGCCGCGGTGCTGGTTA<br>TCCCACGCCCCCACTCTGAATTGGGCGCAATTCTGAGCGTGAGCTCAAAAGGATCGGGCAGCGGGGCCGATTATAAGGATGACGATGACA<br>AGTGAggttcgctgaataaggaggtcagggcATGTCGAGCAACGCCATTGGGGTCATTATTACTAAAGGCACCACAGCTGCCACCGCTGCCGCAGA<br>TGCTATGGTAAAAGCTGCAAATGTGACCCTGACCAGCATAACCAAATGTGGCGATGGCTCAGTGTTGGTGCTGGTAACCGGTGAGGTGGG<br>GGCGGTAAAGGCTGCCACGGAAGCAGGCGCAGAAACTGCGAGTCAAGCCGGCGAGCTGATCGTTGTGTATGTTAGCCCGCGTCCTCATTC<br>GGAACTCGGTGCAGCGGCGAGCGTTAGCTCAAAGGGAAGCGGTAGCGGTCATCATCATCACCATCATTAAtaacaaagctagcataaccccttggg<br>gcctctaaacgggtcttgaggggttttttg
Duo-15A/B	taatacgactcactataggggatctagaggcccttttccaaaggaggttaacatATGAGTTCAAACGCAATCGGGATCATAGAGACAAAAGGAGTGACTGCGG<br>CCACCGCTGCCGCTGATGCTATGGTTAAAGCTGCGAATGTGACCCAAACCGACTTCCGTAGCGACGGTGATGGTTCAGTGTTGGTGCTGGT<br>AACGGGCGAAGTTGGTGCCGTCAAAGCTGCAACTGAAGCCGGCGCTGAAACCGCGTCGCAGAAGGGCGAGCTGTTAATCGTGCGTGTTAT<br>CCCACGCCCCCACTCTGAATTGGGCGCAGCGTTTAGCGTGAGCTCAAAAGGGTCAGGGTCCGGGGCCGATTACAAAGACGACGACGACAA<br>ATGAaatagacgggctaaggaggttcgacgATGAGTTCTAACGCGATCGGGCAGATCAAAACTAAAGGGATGGCTGCCGCAATCGCCGCTGCAGA<br>TGCTATGGTAAAAGCTGCAAATGTGACCGAGACCGCGGTACGTAGCGACGGCGATGGCTCAGTGGCAGTGTTCGTAACCGGTGAGGTGG<br>GGGCGGTAAAGGCTGCCACGGAAGCAGGCGCAGAAACTGCGTCGCAGGGGGGCGAGCTGCAAGAGGTGACCGTTGACCCGCGTCCACA<br>TTCGGAACTCGGCGCAAAATGGTCCGTGTCTTCAAAAGGTAGCGGAAGTGGTCATCACCACCACCATCACTAAtaacaaagctagcataacccctt<br>ggggcctctaaacgggtcttgaggggttttttg
Duo-23A/B	taatacgactcactataggggatctagaggcccttttccaaaggaggttaacatATGAGTTCAAACGCAATCGGGATCATAGAGACAAAAGGAATTGTCGCGG<br>CCATCGCGGCGGCGGACGCGATGTTAAAGGCGGCAAATGTGACTATTACTGCAACACGCAATGACGGTGATGGAAGAGTGCTTGTTCGCG<br>TCACAGGCGAGGTGGGTGCAGTTAAGGCAGCGACGGAGGCAGGCGCGGAGACTGCGTCGCAGGTCGGCGAGTTGTTAAACGTCGGAGT<br>GATTCCTAGGCCTGACAGCACCCTGGGTGCGCTGCTGAGCGTTAGCTCAAAAGGCAGCGGAAGCGGTGCCGACTATAAGGACGACGATGA<br>CAAGTAActcgtttcacctaaggagggactcagATGAGCAGCAATGCGGTGGGAATGATCCAAACCAAAGGCGAGGGCGCTGCAGTAGTAGCCGC<br>AGATGCTATGGTCAAGGCCGCCAACGTTACACTAACCCACGTCCGCAACGACGGAGACGGTAGGGTGACGGTGGTTGTCACCGGAGAAGT<br>TGGAGCCGTAAAAGCAGCCACGGAAGCAGGAGCGGAGACTGCGTCTCAGTGGGGAGAGCTTCTCCAAGTTCATGTCATCCCCAGGCCTCA<br>CTCCGACTTGGGTGCGACATGGAGTGTCTCCAGTAAGGGGTCTGGATCGGGCCATCACCATCATCACCATTAAtaacaaagctagcataaccccttg<br>gggcctctaaacgggtcttgaggggttttttg
Duo-24A/B	taatacgactcactataggggatctagagagcccgggattaaggaggacggcatATGTCCAGCAACGCAATCGGGTTAATAACCACGAAAGGTGCAGTCGCCG<br>CAATGTTCGCCGCTGATGCAATGTTGAAAGCAGCGAATGTGACCCCGACCAGCCGGCAGAGCACAGGTGATGGTATGGACACGGTGTTCG<br>TAACGGGCGAAGTTGGTGCCGTCAAAGCTGCAACTGAAGCCGGCGCTGAAACCGCGTCGCAGATCGGCGAGCTGTTAGAGGTGGCGGTT<br>AACCCACGCCCCAACTCTAGCTTGGGCGCACGTTGGAGCGTGAGCAGCAAAGGCAGCGGAAGCGGTGCCGACTATAAGGACGACGATGA<br>CAAGTAActcgtttcacctaaggagggactcagATGAGCAGCAATGCGTGTGGAGCTATTCAGACAAAAGGACCGACTGCCGCAGTCATGGCTGCA<br>GATGCTATGTTAAAAGCTGCAAACGTGACCCTGACCGACGTACAGAGCACAGGCGATGGCATGGTGGTCGTGATCGTAACCGGTGAGGTG<br>GGGGCGGTAAAGGCTGCCACGGAAGCAGGCGCAGAAACTGCGAGTCAAGTTGGCGAGCTGATCGAGGTGGGCGTTCTGCCGCGTCCTAA<br>CTCGAAATCAGGTGCAATTTGGAGCGTTAGCTCGAAAGGGTCTGGATCGGGCCATCACCATCATCACCATTAAtaacaaagctagcataacccctt<br>ggggcctctaaacgggtcttgaggggttttttg
RMM	taatacgactcactataggggatctagagccccagggtttaaggaggcccacatATGAGTTCGAACGCAATAGGATTAATAGAGACCAAGGGATACGTCGCAG<br>CCCTAGCCGCCGCTGATGCGATGGTTAAAGCCGCCAATGTTACCATTACAGATAGACAACAAGTAGGTGATGGGTTGGTTGCCGTGATCGT<br>CACAGGTGAGGTTGGGGCAGTGAAAGCAGCTACGGAAGCTGGCGCTGAGACTGCCTCTCAAGTAGGAGAGCTCGTGTCGGTACATGTGA<br>TTCCCAGACCGCATTCGGAGCTAGGTGCCCACTTTAGCGTAAGCTCGAAGGGCAGTGGCTCTGGAGCGGACTATAAAGATGACGACGATA<br>AGTAAagggacgcccccaaggagagcaacctATGAGCAGTAACGCCATTGGTCTTATAGAGACCAAGGGTTATGTGGCTGCACTGGCCGCAGCAG<br>ATGCTATGGTCAAAGCGGCTAACGTAACGATCACTGACCGACAACAAGTGGGTGATGGTCTTGTGGCAGTCATTGTCACTGGCGAAGTTG<br>GAGCCGTTAAGGCGGCAACAGAAGCAGGCGCGGAGACGGCAAGCCAGGTAGGTGAATTGGTAAGCGTCCATGTCATCCCAAGGCCACAT<br>AGCGAACTGGGCGCGCACTTCTCAGTGTCTTCAAAAGGAAGCGGTAGCGGGCATCATCATCACCATCATTAAtaacaaagctagcataaccccttg<br>gggcctctaaacgggtcttgaggggttttttg

3 De novo design of nanobodies

3.1 Supplementary Material and Methods

3.1.1 Western blot analyses of nanobodies expression

Soluble protein extracts were analyzed by SDS-PAGE on a commercial 20 % acrylamide gel (run at 180 V for 35 min). Proteins were transferred to PVDF membranes using a wet transfer system (200 mA, 1 h 15 min, 4 °C). Membranes were blocked with 5 % BSA in PBS-Tween, incubated with an anti-His tag antibody directly conjugated to horseradish peroxidase (HRP), and developed using an enhanced chemiluminescence (ECL) substrate. Chemiluminescent signals were detected using an imaging system.

3.1.2 Expression and purification of SARS-CoV-2 RBDs, and MR17 and NbRM-E1 nanobodies

Bacterial Expression of MR17 and NbRM-E1 nanobodies Culture of 500 mL of Terrific Broth (TB) medium (enclosed in 2 L Erlenmeyer flasks) was inoculated to an initial OD_{600nm} of 0.1 and grown at 37 °C, 200 rpm. Protein expression was induced with 1 mM IPTG when an OD_{600nm} between 0.5 and 0.8 was reached and incubation was continued overnight at 28 °C, 200 rpm. Cells were then harvested by centrifugation at 3,000 g for 20 min at 4 °C. Pellets were resuspended in lysis buffer (50 mM Tris-HCl pH 8, 1 M NaCl, 5 mM imidazole) in a volume = (OD × culture volume)/100. Resuspended pellets were flash-frozen and thawed in a 37 °C water bath for approximately 15 min. Lysis was enhanced by adding 1:100 PMSF (1 mM), 1:50 DNase I ($2\,mg \cdot mL^{-1}$), 1:200 lysozyme ($0.5\,mg \cdot mL^{-1}$), and 1:400 $MgSO_4$ (2 M), followed by incubation on a rotating wheel for 1 h at 4 °C. Cells were disrupted by sonication (amplitude 60 %, 1 s on / 1 s off, 3 cycles of 30 seconds). The lysate was clarified by centrifugation at 15,000 g for 30 min at 4 °C. The supernatant was collected for purification and samples of both pellet and supernatant were retained for SDS-PAGE analysis.

Nanobody Purification Affinity purification was performed using a nickel-charged resin. Buffer A consisted of 50 mM Tris-HCl pH 8, 300 mM NaCl, 5 mM imidazole, and 5 % glycerol. Buffer B was identical, except for a higher imidazole concentration (500 mM). The column was equilibrated with Buffer A before loading the clarified lysate. The flow-through was collected for SDS-PAGE analysis. After washing with 10 % Buffer B, elution was performed with 50 % Buffer B, collecting 1.5 mL fractions. The peak fractions were pooled, and a sample was retained for SDS-PAGE. The column was subsequently washed with 100 % Buffer B. The pooled elution was concentrated using a 10 kDa molecular weight cut-off centrifugal concentrator to a final volume between 1 and 2 mL. The concentrated protein sample was further purified by size-exclusion chromatography using a Superdex 75 16/60 column (Cytiva) equilibrated in PBS (1×). The sample was loaded onto the column and the MR17 nanobodies were eluted between 50 and 70 mL.

Production of the receptor binding domains (RBDs) of the SARS-CoV-2 spikes SARS-CoV-2 RBD (223 amino acids starting at position 319 of the spike sequence) coding sequences were cloned in frame behind a sequence encoding a signal peptide and in front of a His-tag coding sequence in the eukaryotic pYD11 expression plasmid. The resulting plasmids were transfected with PEImax (24765–1) (Polysciences, Inc.) in EXPI-293F cells (A14527) (Thermofisher). Transfected cells were then maintained in EXPI expression medium (Gibco, Thermofisher). Cells were removed by mild centrifugation at day+7, and RBDs were extracted from the cell culture medium by Ni^{2+} affinity chromatography followed by gel filtration. Purified RBDs were used for binding and affinity experiments by BLI.

3.1.3 Molecular Dynamics simulations of RBD-nanobody complexes

Molecular dynamics (MD) simulations were performed on designed nanobodies in complex with the SARS-CoV-2 RBD XBB.1.16 to support the selection of candidates for subsequent experimental characterization. Structural models of designed nanobody/RBD complexes were constructed using Custozyme+ and refined using the Rosetta FastRelax protocol with the *beta_nov16* score function.[30,36] Protonation states of titrable residues were assigned at pH 6.5, using the pdb2pqr server,[10,19] resulting in the protonation of Glu6 in the nanobodies. The all-atom ff14SB AMBER force field[25] was used and the complex was solvated with TIP3P water molecules in a truncated octahedral box with a minimum distance of 0.15 nm between the protein and the simulation box edge. Periodic boundary conditions were applied and ions Na^+ or Cl^- were added to reach a

150 mM concentration with the required counterions to neutralize the system. The number of ions to be added to each system was calculated using the SPLIT method described by Machado and Pantano.[24]

Energy minimization was performed in successive steps using steepest descent and conjugate gradient algorithms. Systems were then gradually heated to 310 K over 100 ps in the NVT ensemble. A short 20 ps equilibration was then performed in the NVT ensemble, followed by 200 ps under NPT conditions, at 310 K and 1 bar. Temperature and pressure were maintained constant using the Berendsen thermostat and barostat algorithms,[4] both with coupling constants of 2 ps. Long-range electrostatic interactions were treated using the particle-mesh Ewald (PME) method,[7,13] with a 12 Å cut-off radius. The SHAKE algorithm was applied to constrain all bond lengths involving hydrogen atoms to their equilibrium values.[33] Protein backbone atoms were initially restrained to their initial position using a harmonic potential, which was progressively removed throughout the preparation procedure. Production phases were carried out for 50 ns in the NPT ensemble using AMBER 18,[5] with a 2 fs integration time-step.

Interfacial contacts and hydrogen bonds between the nanobody and the RBD were quantified over the course of the 50 ns MD simulation using the cpptraj module from the AMBER package.[32] Binding free energies, ΔG, were estimated using the molecular mechanics generalized Born surface area (MM/GBSA) and Poisson-Boltzmann surface area (MM/PBSA) approaches,[14,15,17,20] as implemented in the mmpbsa.py[27] tool from the AMBER 18 package. Calculations were performed on either 50 snapshots extracted each nanosecond over the 50 ns trajectory or on 100 snapshots sampled every 100 ps over the last 10 ns (40-50 ns). The standard parameters reported by Miller et al. for calculations with AMBER program mmpbsa.py[27] were used, with the default implemented GBSA method.[28]

3.2 Figures and Tables

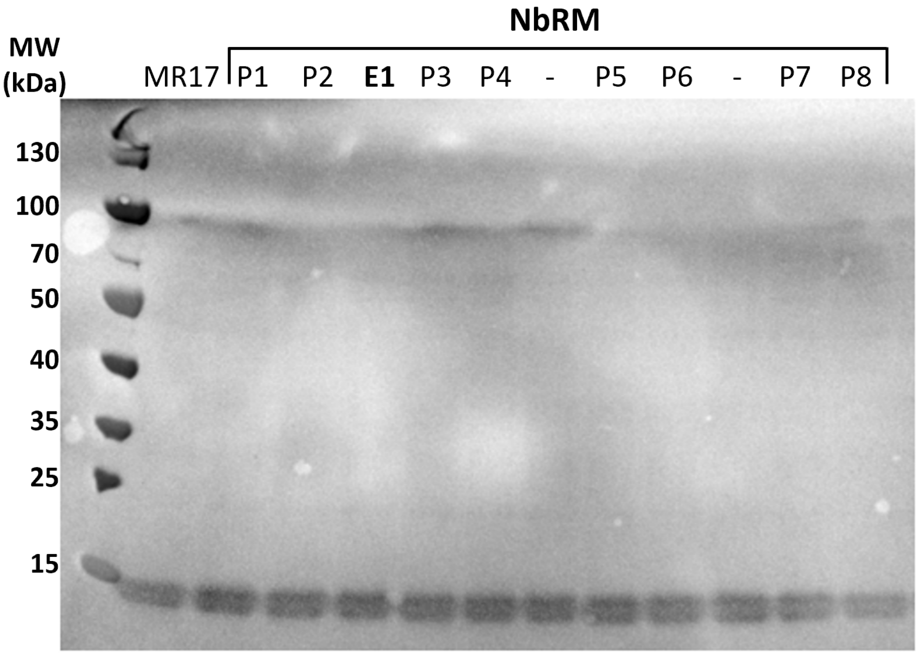

Figure S20. Expression of designed nanobodies verified by western blot on total cell extracts, using His-tag specific antibodies. Nanobodies were detected at ∼15 kDa, as expected. All designed nanobodies are correctly expressed in *E. coli*.

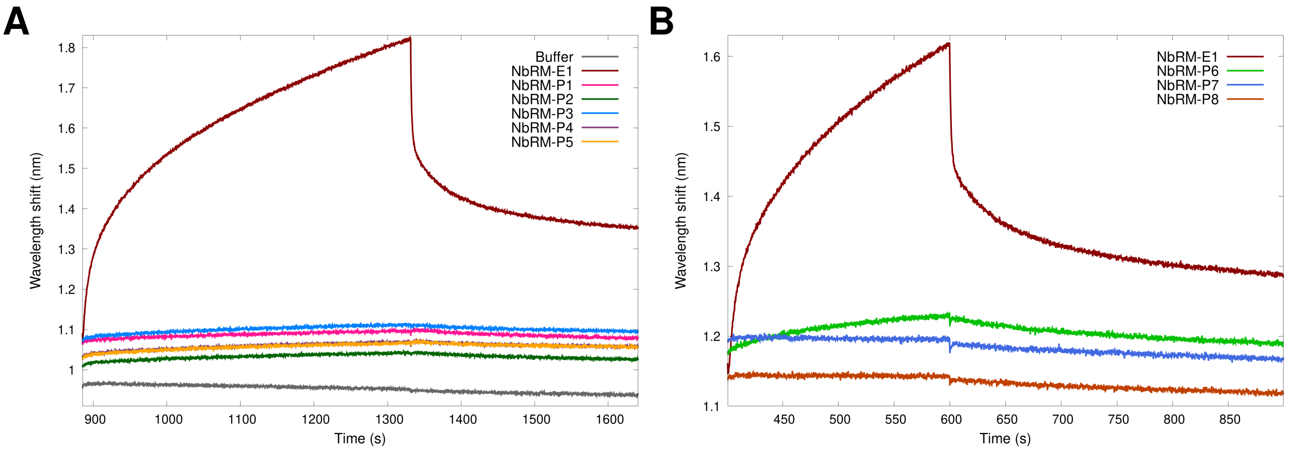

Figure S21. Interaction of designed nanobodies with SARS-CoV-2 RBD XBB.1.16 assessed by BLI experiments on bacterial extracts. RBD was fixed on the biosensor via its His-tag and it was dipped into total cell extracts for 400 s (association). The biosensor was then dipped in assay buffer for a dissociation step. Only NbRM-E1 (predicted by EffieDes) shows a wavelength shift characteristic of binding to the target RBD, whereas none of the nanobodies predicted by ProteinMPNN (denoted NbRM-Ps) interact with the XBB.1.16 RBD.

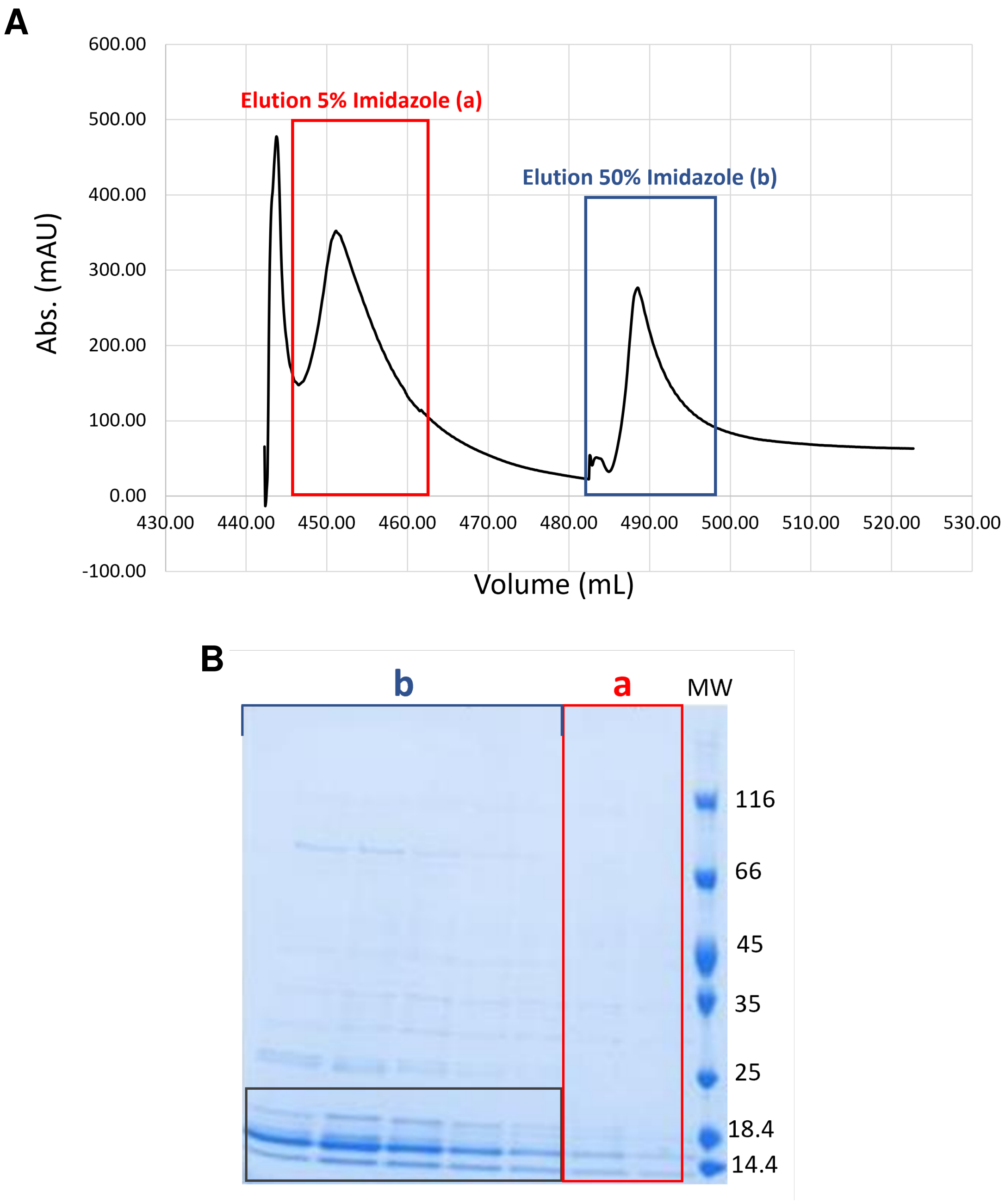

Figure S22. Purification by Nickel-affinity chromatography of the NbRM-E1 nanobody. (**A**) Chromatogram of the affinity purification on a nickel-charged resin with two peaks recovered after elution with 5 % (a) or 50 % (b) imidazole. (**B**) SDS-PAGE of the two elution peaks showing NbRM-E1 in the fractions eluted with 50 % imidazole (peak b), highlighted by the black frame.

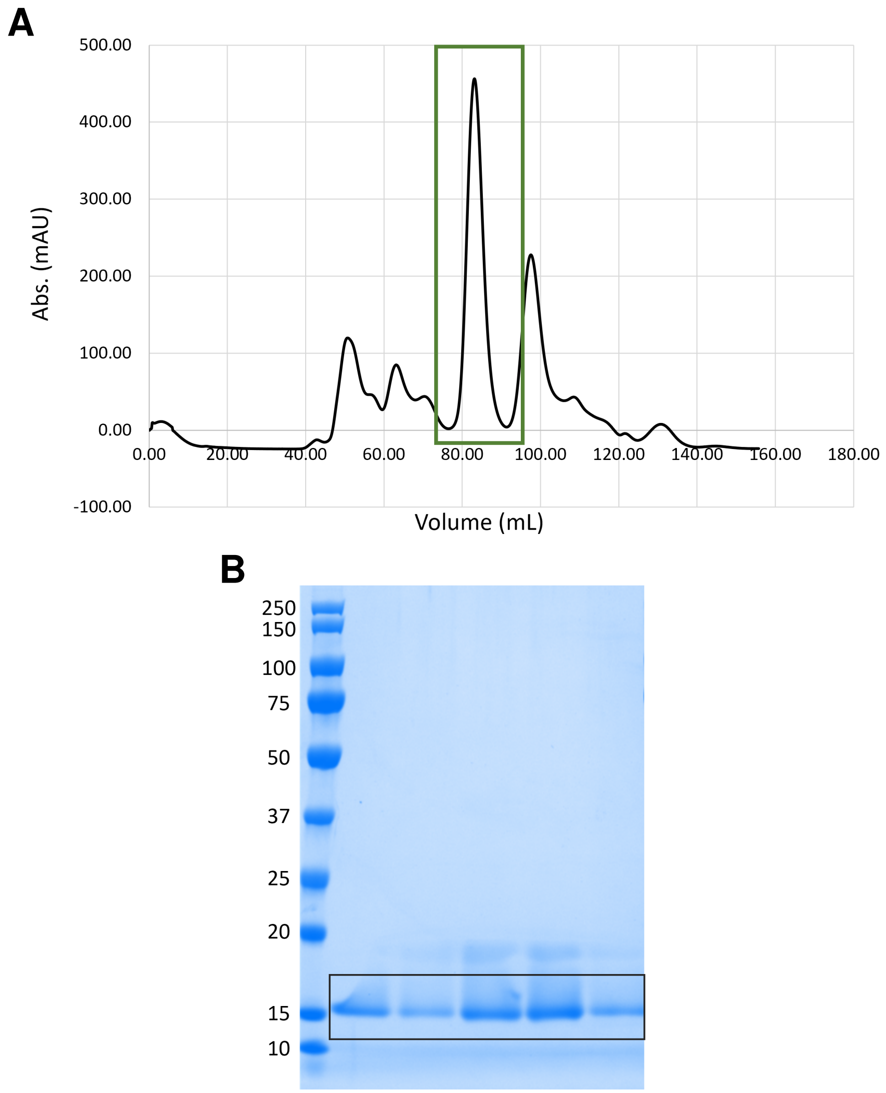

Figure S23. Purification by gel filtration of the NbRM-E1 nanobody. (**A**) Chromatogram with the peak containing the nanobody delimited in green. (**B**) SDS-PAGE of the eluted fractions containing the nanobody.

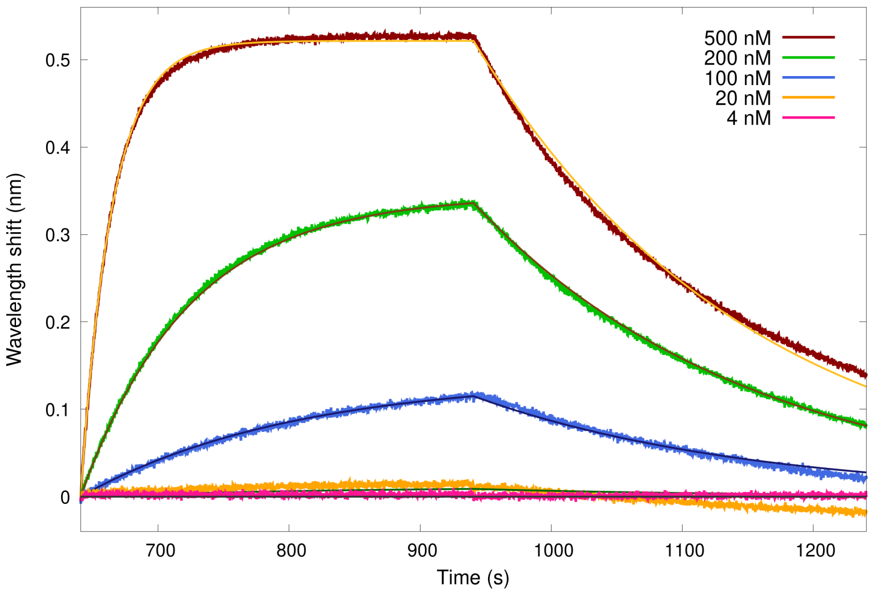

Figure S24. BLI-based determination of the dissociation constant K_D for the NbRM-E1/XBB.1.16 RBD binding. Association and dissociation curves of NbRM-E1 with XBB.1.16 RBD were generated for different concentrations of nanobody and they were fitted to the "association then dissociation" equations for a 1:1 binding model using the FortéBio Data analysis software, resulting in $K_{D\ \mathrm{NbRM\text{-}E1}} = 64$ nM with $k_{on} = 7.4\ 10^4\ M^{-1}\ s^{-1}$ and $k_{off} = 4.74\ 10^{-3}\ s^{-1}$.

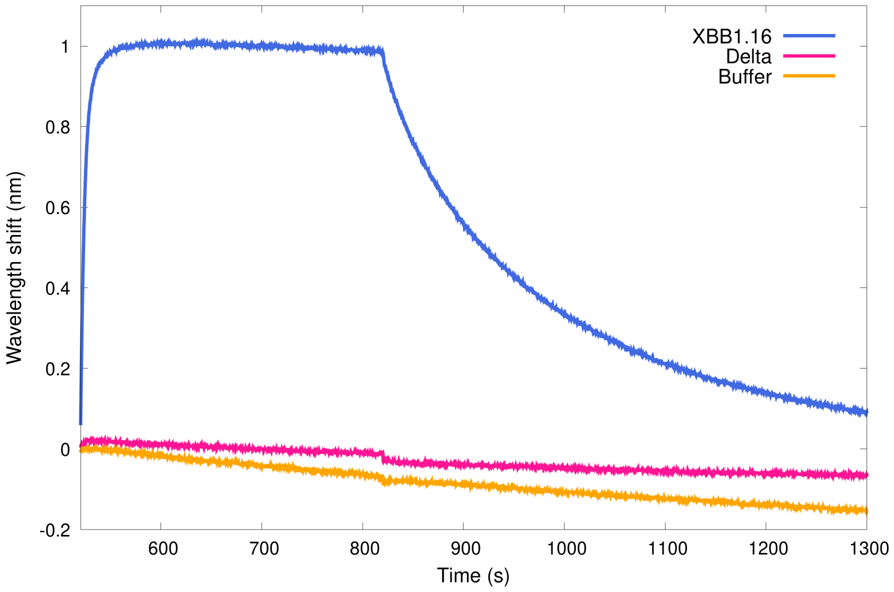

Figure S25. BLI specificity assay showing that NbRM-E1 binds to the target SARS-CoV-2 XBB.1.16 RBD (blue) but not to the Delta variant RBD (pink).

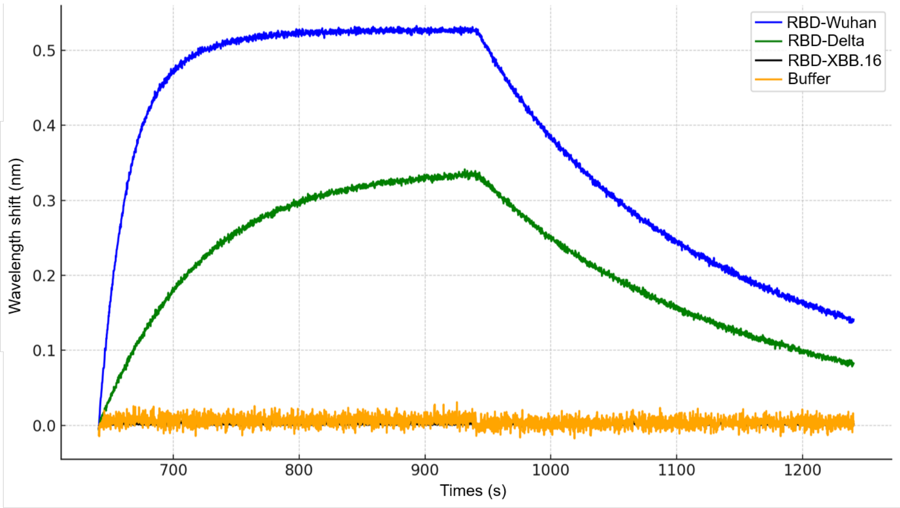

Figure S26. BLI specificity assay of MR17 nanobody binding to SARS-CoV-2 RBD variants. MR17 binds to both the Wuhan and Delta RBDs but shows no detectable interaction with the XBB.1.16 RBD.

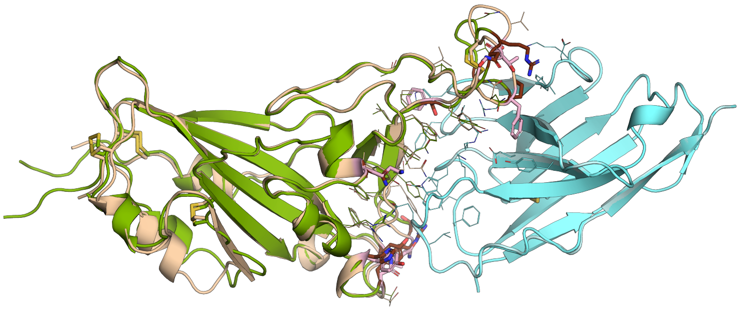

Figure S27. Comparison of SARS-CoV-2 mutations affecting MR17 interaction with the RBD. Representation of the interaction of the MR17 nanobody (blue) to the SARS-CoV-2 RBD. The SARS-CoV-2 Wuhan RBD (from the crystal structure of its complex with MR17 (PDB ID: 7C8W)[22]) is shown as a beige cartoon. The backbone of the XBB.1.16 RBD (green) was aligned to the Wuhan RBD. Interface residues between MR17 and the RBDs are depicted as lines. The 11 mutations between the Wuhan RBD and the XBB.1.16 RBD, localized at the interface with the MR17 nanobody, are represented in sticks, and colored in pink (Wuhan) or dark brown (XBB.1.16): K417N, V445P, G446S, S477N, T478R, E484A, F486P, F490S, Q498R, N501Y and Y505H. Disulfide bonds are shown as sticks, and atoms are colored by type in both line and stick representations.

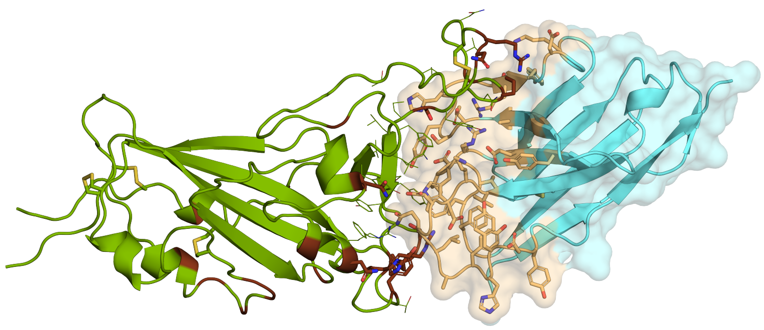

Figure S28. 3D structural model of MR17 (in blue) in complex with the SARS-CoV-2 XBB.1.16 RBD (in green). The 36 designable residues on MR17 are highlighted as orange sticks. Mutations between the Wuhan and the XBB.1.16 RBDs are colored in dark brown, and those located at the MR17-RBD interface also represented as sticks. Disulfide bonds are shown as sticks, and atoms are colored by type in both line and stick representations.

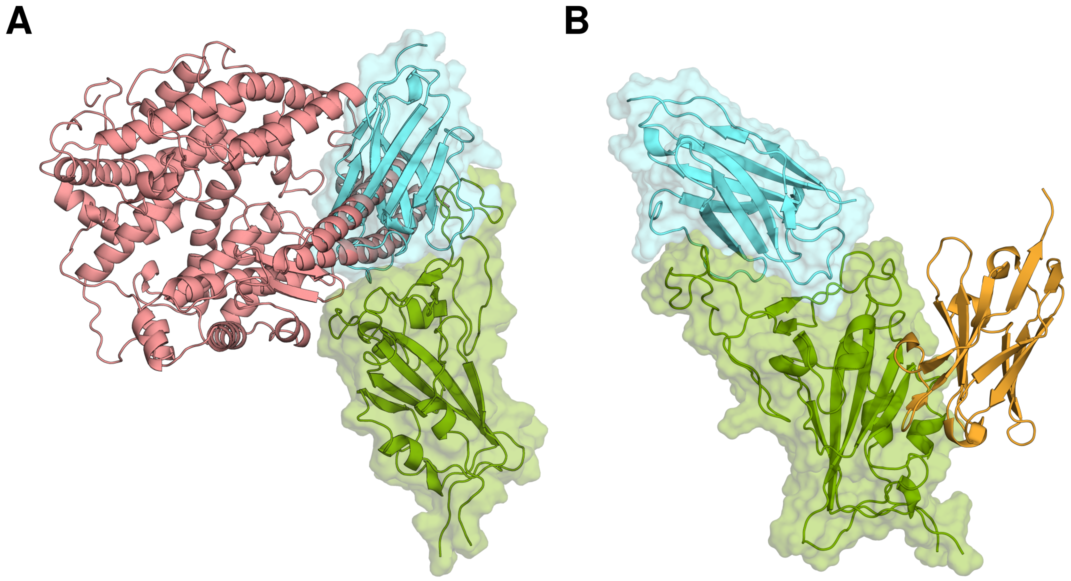

Figure S29. 3D structural models of the competitive binding of NbRM-E1 with ACE2 and VHH-72 on the SARS-CoV-2 XBB.1.16 RBD. Structural model of NbRM-E1 (blue) in complex with the SARS-CoV-2 XBB.1.16 RBD (green), shown in cartoon representation with a transparent surface, after Rosetta relaxation. (**A**) ACE2 (pink) and (**B**) VHH-72 (orange) are shown in complex with the XBB.1.16 RBD following structural alignment of either the SARS-CoV-2 XBB.1.5 RBD backbone from the cryo-EM structure of the ACE2/RBD complex (PDB ID: 8VKP)[26] or the SARS-CoV-1 RBD backbone from the crystal structure of the VHH-72/RBD complex (PDB ID: 6WAQ).[38]

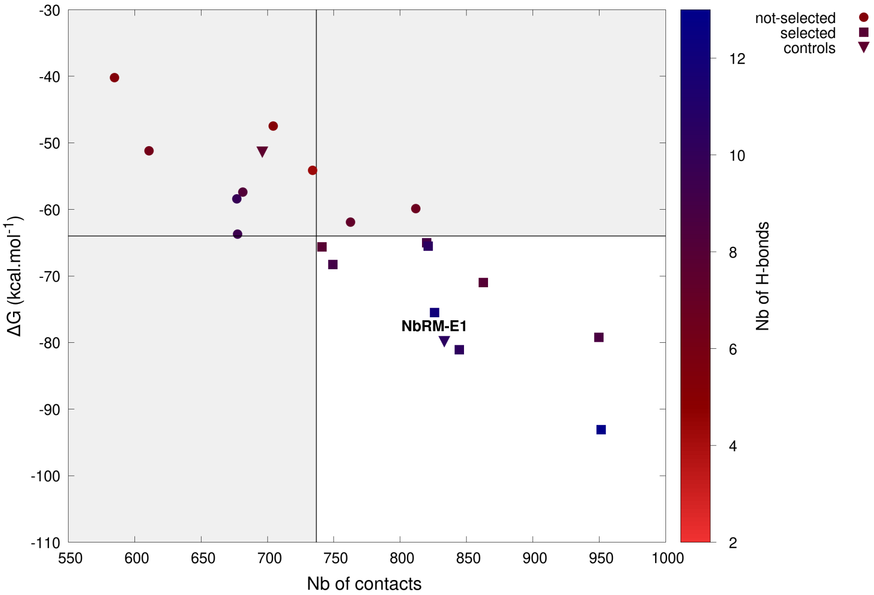

Figure S30. Selection of designed nanobodies based on MM/GBSA binding affinity estimates, interface size and interfacial hydrogen bonds during MD simulations. Binding affinities were calculated by MM/GBSA over the last 10 ns of simulation (40-50 ns) and the number of interfacial contacts and hydrogen bonds were averaged over the same time window. Sequences represented by squares in the white area of the graph were selected for experimental testing. Controls correspond to simulations of the MR17/Wuhan RBD and MR17/XBB.1.16 RBD complexes.

Table S10. Rosetta InterfaceAnalyzer metrics used for RBD/nanobody complexes selection for MD simulations. Only results obtained for the 9 experimentally tested nanobodies are shown and the number of mutations compared to the MR17 sequence is indicated. Binding free energy ΔG, normalized cross-interface energy per buried surface area ($\frac{\Delta G_{cross}}{dSASA}$), number of interface hydrogen bonds, number of aromatic and total interface residues, and buried solvent-accessible surface area at the interface (dSASA) are displayed. dSASA indicates the extent of surface buried upon complex formation and reflect interface complementarity and stability (higher values indicate more stable interfaces), while $\frac{\Delta G_{cross}}{dSASA}$ provides a normalized measure of interaction strength accounting for interface size (lower values corresponds to stronger interactions).

	design method	mut.	ΔG	$\frac{\Delta G_{cross}}{dSASA}$	H-bonds	aromatics	total residues	dSASA
XBB.1.16+MR17	-	-	-41.8	-0.0291	10	10	59	1469.0
Wuhan+MR17	-	-	-49.5	-0.0310	10	14	70	1868.6
NbRM-E1†	EffieDes	29	-54.3	-0.0326	17	11	77	1810.3
NbRM-P1†	ProteinMPNN	31	-50.7	-0.0269	11	12	86	2157.1
NbRM-P2	ProteinMPNN	31	-56.4	-0.0284	13	9	84	2175.6
NbRM-P3	ProteinMPNN	30	-54.1	-0.0310	14	10	84	1948.9
NbRM-P4	ProteinMPNN	28	-60.3	-0.0345	15	11	90	1868.1
NbRM-P5	ProteinMPNN	30	-55.9	-0.0334	13	11	87	1921.3
NbRM-P6	ProteinMPNN	27	-49.4	-0.0298	8	12	79	1748.3
NbRM-P7	ProteinMPNN	28	-56.3	-0.0298	14	11	86	2080.5
NbRM-P8	ProteinMPNN	28	-62.3	-0.0315	13	9	79	2011.2

† NbRM-E1 and NbRM-P1 were designed from the same structural template.

Table S11. Interaction between nanobodies and SARS-CoV-2 RBDs computed by MM/GBSA or MM/PBSA and calculations of the number of hydrogen bonds and contacts over the 50 ns of MD simulation.

			0-50 ns			
	design method	mut.	MM/GBSA	MM/PBSA	H-bonds	contacts
Wuhan+MR17	-	-	-79.6 ± 9.2	-13.1 ± 6.8	9.8 ± 2.3	856.0 ± 66.5
XBB.1.16+MR17	-	-	-52.9 ± 8.3	3.4 ± 5.2	7.9 ± 2.2	688.4 ± 55.8
NbRM-E1	EffieDes	29	-85.3 ± 16.1	-13.7 ± 8.9	12.5 ± 2.7	882.9 ± 99.8
NbRM-P1	ProteinMPNN	31	-89.6 ± 8.1	-5.7 ± 6.6	12.1 ± 2.2	920.0 ± 67.2
NbRM-P2	ProteinMPNN	31	-81.2 ± 8.9	-10.3 ± 6.3	9.7 ± 2.3	874.9 ± 61.8
NbRM-P3	ProteinMPNN	30	-68.7 ± 12.9	3.0 ± 8.3	6.9 ± 2.4	868.8 ± 95.0
NbRM-P4	ProteinMPNN	28	-63.3 ± 13.8	-5.0 ± 7.3	7.4 ± 2.6	836.0 ± 123.3
NbRM-P5	ProteinMPNN	30	-60.2 ± 11.0	2.2 ± 7.3	8.8 ± 2.1	712.2 ± 77.5
NbRM-P6	ProteinMPNN	27	-71.1 ± 7.1	-5.6 ± 5.7	9.4 ± 2.0	822.0 ± 83.2
NbRM-P7	ProteinMPNN	28	-65.9 ± 15.1	6.0 ± 11.8	10.1 ± 2.3	783.6 ± 84.7
NbRM-P8	ProteinMPNN	28	-68.5 ± 7.5	-8.0 ± 5.7	8.3 ± 2.1	799.3 ± 85.5

Table S12. Interaction between nanobodies and SARS-CoV-2 RBDs evaluated using MM/GBSA or MM/PBSA and calculations of the number of hydrogen bonds and contacts during the last 10 ns of MD simulations (40 to 50 ns).

			40-50 ns			
	design method	mut.	MM/GBSA	MM/PBSA	H-bonds	contacts
Wuhan+MR17	-	-	-79.8 ± 6.3	-14.1 ± 5.6	10.4 ± 2.1	833.2 ± 60.5
XBB.1.16+MR17	-	-	-51.3 ± 5.6	4.9 ± 4.8	7.5 ± 1.8	696.2 ± 49.9
NbRM-E1	EffieDes	29	-75.5 ± 7.7	-10.6 ± 6.1	12.0 ± 2.0	825.8 ± 61.0
NbRM-P1	ProteinMPNN	31	-93.1 ± 8.5	-8.4 ± 6.6	12.9 ± 2.2	951.5 ± 74.4
NbRM-P2	ProteinMPNN	31	-81.1 ± 6.8	-9.8 ± 5.1	10.3 ± 2.0	844.6 ± 55.0
NbRM-P3	ProteinMPNN	30	-79.2 ± 7.9	-4.0 ± 7.0	8.6 ± 2.5	949.8 ± 67.2
NbRM-P4	ProteinMPNN	28	-71.0 ± 6.6	-5.8 ± 5.0	7.9 ± 2.1	862.6 ± 73.2
NbRM-P5	ProteinMPNN	30	-68.3 ± 9.4	-3.3 ± 5.9	9.0 ± 2.3	749.2 ± 62.7
NbRM-P6	ProteinMPNN	27	-65.0 ± 7.9	-2.4 ± 5.8	8.9 ± 1.8	820.0 ± 76.3
NbRM-P7	ProteinMPNN	28	-65.5 ± 8.9	3.6 ± 8.3	10.4 ± 2.2	821.0 ± 60.0
NbRM-P8	ProteinMPNN	28	-65.6 ± 6.5	-6.2 ± 4.4	7.8 ± 2.0	741.1 ± 57.3

Table S13. Amino acid sequences of designed nanobodies and number of mutations relative to MR17.

Protein	Nb mut.	Amino acids sequence
MR17	-	QVQLVESGGGLVQAGGSLRLSCAASGFPVEVWRMEWYRQAP<br>GKEREGVAAIESYGHGTRYADSVKGRFTISRDNAKNTVYLQ<br>MNSLKPEDTAVYYCNVKDDGQLAYHYDYWGQGTQVTVSAG
NbRM-E1	26	QVQLVESGGGLVQAGGSLRLSCAASGLDLREHQMRWYRQAP<br>GKEREEVALIDASGAEPGAADSVKGRFTISRDNAKNTVYLQ<br>MNSLKPEDTAVYYCNVVGSGYGSGPEGAWGQGTQVTVSAG
NbRM-P1	31	QVQLVESGGGLVQAGGSLRLSCAASGGNINENTMVWYRQAP<br>GKEREQVAVISANGRTPGVAESVKGRFTISRDNAKNTVYLQ<br>MNSLKPEDTAVYYCNQYNSGYGQGPVGLWGQGTQVTVSAG
NbRM-P2	31	QVQLVESGGGLVQAGGSLRLSCAASGLDINFSDMEWWRQAP<br>GKERELVARIASGGSKPGVAESVKGRFTISRDNAKNTVYLQ<br>MNSLKPEDTAVYYCNFLTSSLNEMPSGLWGQGTQVTVSAG
NbRM-P3	30	QVQLVESGGGLVQAGGSLRLSCAASGISLNDGTMVWYRQAP<br>GKEREEVAVIDMSGRKPGVADSVKGRFTISRDNAKNTVYLQ<br>MNSLKPEDTAVYYCNLVGSDSIPRSPDLWGQGTQVTVSAG
NbRM-P4	28	QVQLVESGGGLVQAGGSLRLSCAASGIDLSAYQMVWYRQAP<br>GKEREEVAAIAPNGGSSGVADSVKGRFTISRDNAKNTVYLQ<br>MNSLKPEDTAVYYCNVVAGGGANLGPSTWGQGTQVTVSAG
NbRM-P5	30	QVQLVESGGGLVQAGGSLRLSCAASGADARQFTMTWYRQAP<br>GKEREEVAAIDMSGGKAGVADSVKGRFTISRDNAKNTVYLQ<br>MNSLKPEDTAVYYCNLLYTPANQLPTSSWGQGTQVTVSAG
NbRM-P6	27	QVQLVESGGGLVQAGGSLRLSCAASGVPVSAYTMSWYRQAP<br>GKEREAVAAISGDGTGPGTAPSVKGRFTISRDNAKNTVYLQ<br>MNSLKPEDTAVYYCNLLIGDTIPYEPGVWGQGTQVTVSAG
NbRM-P7	28	QVQLVESGGGLVQAGGSLRLSCAASGIDLSRYDMEWYRQAP<br>GKEREGVAAISADGRTPSVADSVKGRFTISRDNAKNTVYLQ<br>MNSLKPEDTAVYYCNLVLSEADPAVVNVWGQGTQVTVSAG
NbRM-P8	28	QVQLVESGGGLVQAGGSLRLSCAASGMDLSQQRMEWWRQAP<br>GKERELVAVIYGNGLGSGVADSVKGRFTISRDNAKNTVYLQ<br>MNSLKPEDTAVYYCNLLTSGLQELPSGTWGQGTQVTVSAG

References

1. Allouche, D, de Givry, S, Katsirelos, G, Schiex, T & Zytnicki, M. *Anytime hybrid best-first search with tree decomposition for weighted CSP* in *Proc. of CP-15* (Cork, Ireland, 2015), 12–28. doi:10.1007/978-3-319-23219-5_2.
2. Allouche, D., De Givry, S., Katsirelos, G., Schiex, T. & Zytnicki, M. *Anytime hybrid best-first search with tree decomposition for weighted CSP* in *International Conference on Principles and Practice of Constraint Programming* (2015), 12–29. doi:10.1007/978-3-319-23219-5_2.
3. Aneja, Y. P. & Nair, K. P. K. Bicriteria transportation problem. *Management Science* **25,** 73–78. doi:10.1287/mnsc.25.1.73 (1979).
4. Berendsen, H. J. C., Postma, J. P. M., van Gunsteren, W. F., DiNola, A. & Haak, J. R. Molecular dynamics with coupling to an external bath. *J. Chem. Phys.* **81,** 3684–3690. ISSN: 0021-9606. doi:10.1063/1.448118 (1984).
5. Case, D. *et al. AMBER 2018* University of California, San Francisco, 2018. https://ambermd.org/doc12/Amber18.pdf.
6. Charpentier, A. *et al.* Variable neighborhood search with cost function networks to solve large computational protein design problems. *J. Chem. Inf. Model.* **59,** 127–136. ISSN: 1549-9596. doi:10.1021/acs.jcim.8b00510 (2019).
7. Darden, T., York, D. & Pedersen, L. Particle mesh Ewald: An N·log(N) method for Ewald sums in large systems. *J. Chem. Phys.* **98,** 10089–10092. ISSN: 0021-9606. doi:10.1063/1.464397 (1993).
8. Dauparas, J. *et al.* Robust deep learning-based protein sequence design using ProteinMPNN. *Science* **378,** 49–56. doi:10.1126/science.add2187 (2022).
9. Defresne, M., Barbe, S. & Schiex, T. *Scalable coupling of deep learning with logical reasoning* in *Proceedings of the Thirty-Second International Joint Conference on Artificial Intelligence, IJCAI-23* (2023), 3615–3623. doi:10.24963/ijcai.2023/402.
10. Dolinsky, T. J., Nielsen, J. E., McCammon, J. A. & Baker, N. A. PDB2PQR: an automated pipeline for the setup of Poisson-Boltzmann electrostatics calculations. *Nucleic Acids Res.* **32,** W665–W667. ISSN: 0305-1048. doi:10.1093/nar/gkh381 (2004).
11. Durante, V., Katsirelos, G. & Schiex, T. *Efficient low rank convex bounds for pairwise discrete Graphical Models* in *Proceedings of the 39th International Conference on Machine Learning* (eds Chaudhuri, K. *et al.*) **162** (PMLR, 2022), 5726–5741.
12. Ehrgott, M., Gandibleux, X. & Przybylski, A. in *Multiple Criteria Decision Analysis: State of the Art Surveys* (eds Greco, S., Ehrgott, M. & Figueira, J. R.) 817–850 (Springer, 2016). ISBN: 978-1-4939-3094-4. doi:10.1007/978-1-4939-3094-4_19.
13. Essmann, U. *et al.* A smooth particle mesh Ewald method. *J. Chem. Phys.* **103,** 8577–8593. ISSN: 0021-9606. doi:10.1063/1.470117 (1995).
14. Feig, M. *et al.* Performance comparison of generalized born and Poisson methods in the calculation of electrostatic solvation energies for protein structures. *J. Comput. Chem.* **25,** 265–284. ISSN: 1096-987X. doi:10.1002/jcc.10378 (2004).
15. Gohlke, H. & Case, D. A. Converging free energy estimates: MM-PB(GB)SA studies on the protein–protein complex Ras–Raf. de. *J. Comput. Chem.* **25,** 238–250. ISSN: 1096-987X. doi:10.1002/jcc.10379 (2004).
16. Hallen, M. A. & Donald, B. R. Protein design by provable algorithms. *Commun. ACM* **62,** 76–84. doi:10.1145/3338124 (2019).
17. Hou, T., Wang, J., Li, Y. & Wang, W. Assessing the performance of the MM/PBSA and MM/GBSA methods. 1. The accuracy of binding free energy calculations based on molecular dynamics simulations. *J. Chem. Inf. Model.* **51,** 69–82. ISSN: 1549-9596. doi:10.1021/ci100275a (2011).
18. Hurley, B. *et al.* Multi-language evaluation of exact solvers in graphical model discrete optimization. *Constraints* **21,** 413–434. ISSN: 1572-9354. doi:10.1007/s10601-016-9245-y (2016).
19. Jurrus, E. *et al.* Improvements to the APBS biomolecular solvation software suite. *Protein Sci.* **27,** 112–128. doi:10.1002/pro.3280 (2018).

20. Kollman, P. A. *et al.* Calculating structures and free energies of complex molecules: Combining molecular mechanics and continuum models. *Acc. Chem. Res.* **33,** 889–897. ISSN: 0001-4842. doi:10.1021/ar000033j (2000).
21. Li, G.-W., Burkhardt, D., Gross, C. & Weissman, J. S. Quantifying absolute protein synthesis rates reveals principles underlying allocation of cellular resources. *Cell* **157,** 624–635. ISSN: 0092-8674. doi:10.1016/j.cell.2014.02.033 (2014).
22. Li, T. *et al.* A synthetic nanobody targeting RBD protects hamsters from SARS-CoV-2 infection. *Nat. Commun.* **12,** 4635. ISSN: 2041-1723. doi:10.1038/s41467-021-24905-z (2021).
23. Liu, H., Dai, Z., So, D. R. & Le, Q. V. *Pay Attention to MLPs* in *Advances in Neural Information Processing Systems 34: Annual Conference on Neural Information Processing Systems 2021, NeurIPS 2021, December 6-14, 2021, virtual* (eds Ranzato, M., Beygelzimer, A., Dauphin, Y. N., Liang, P. & Vaughan, J. W.) (2021), 9204–9215. doi:10.48550/arXiv.2105.08050. https://proceedings.neurips.cc/paper/2021/hash/4cc05b35c2f937c5bd9e7d41d3686fff-Abstract.html.
24. Machado, M. R. & Pantano, S. Split the charge difference in two! A rule of thumb for adding proper amounts of ions in MD simulations. *J. Chem. Theory Comput.* **16,** 1367–1372. doi:10.1021/acs.jctc.9b00953 (2020).
25. Maier, J. A. *et al.* ff14SB: Improving the accuracy of protein side chain and backbone parameters from ff99SB. *J. Chem. Theory Comput.* **11,** 3696–3713. doi:10.1021/acs.jctc.5b00255 (2015).
26. Mannar, D. *et al.* Altered receptor binding, antibody evasion and retention of T cell recognition by the SARS-CoV-2 XBB.1.5 spike protein. *Nat. Commun.* **15,** 1854. ISSN: 2041-1723. doi:10.1038/s41467-024-46104-2 (2024).
27. Miller, B. R. I. *et al.* MMPBSA.py: An efficient program for end-state free energy calculations. *J. Chem. Theory Comput.* **8,** 3314–3321. doi:10.1021/ct300418h (2012).
28. Onufriev, A., Bashford, D. & Case, D. A. Exploring protein native states and large-scale conformational changes with a modified generalized born model. *Proteins:Struct., Funct., Bioinf.* **55,** 383–394. doi:https://doi.org/10.1002/prot.20033 (2004).
29. Ouali, A. *et al.* Variable neighborhood search for graphical model energy minimization. *Artif. Intell.* **278,** 103194. ISSN: 0004-3702. doi:10.1016/j.artint.2019.103194 (2020).
30. Pavlovicz, R. E., Park, H. & DiMaio, F. Efficient consideration of coordinated water molecules improves computational protein-protein and protein-ligand docking discrimination. *PLoS Comput. Biol.* **16,** e1008103. ISSN: 1553-7358. doi:10.1371/journal.pcbi.1008103 (2020).
31. Pierce, N. A. & Winfree, E. Protein design is NP-hard. *Protein Eng., Des. Sel.* **15,** 779–782. ISSN: 1741-0126. doi:10.1093/protein/15.10.779 (2002).
32. Roe, D. R. & Cheatham, T. E. I. PTRAJ and CPPTRAJ: Software for processing and analysis of molecular dynamics trajectory data. *J. Chem. Theory Comput.* **9,** 3084–3095. doi:10.1021/ct400341p (2013).
33. Ryckaert, J.-P., Ciccotti, G. & Berendsen, H. J. Numerical integration of the cartesian equations of motion of a system with constraints: molecular dynamics of n-alkanes. *J. Comput. Phys.* **23,** 327–341. ISSN: 0021-9991. doi:10.1016/0021-9991(77)90098-5 (1977).
34. Steinegger, M. & Söding, J. MMseqs2 enables sensitive protein sequence searching for the analysis of massive data sets. *Nat. Biotechnol.* **35,** 1026–1028. ISSN: 1546-1696. doi:10.1038/nbt.3988 (2017).
35. Stockmeyer, L. J. The polynomial-time hierarchy. *Theoretical Computer Science* **3,** 1–22 (1976).
36. Tyka, M. D. *et al.* Alternate states of proteins revealed by detailed energy landscape mapping. *J. Mol. Biol.* **405,** 607–618. ISSN: 0022-2836. doi:10.1016/j.jmb.2010.11.008 (2011).
37. Vucinic, J., Simoncini, D., Ruffini, M., Barbe, S. & Schiex, T. Positive multistate protein design. *Bioinformatics* **36,** 122–130. ISSN: 1367-4803. doi:10.1093/bioinformatics/btz497 (2020).
38. Wrapp, D. *et al.* Structural basis for potent neutralization of Betacoronaviruses by single-domain camelid antibodies. *Cell* **181,** 1004–1015.e15. ISSN: 0092-8674, 1097-4172. doi:10.1016/j.cell.2020.04.031 (2020).
